# Design and synthesis of biphenyl and biphenyl ether inhibitors of sulfatases†
†Electronic supplementary information (ESI) available. See DOI: 10.1039/c5sc03612g


**DOI:** 10.1039/c5sc03612g

**Published:** 2016-01-11

**Authors:** Tristan Reuillon, Sari F. Alhasan, Gary S. Beale, Annalisa Bertoli, Alfie Brennan, Celine Cano, Helen L. Reeves, David R. Newell, Bernard T. Golding, Duncan C. Miller, Roger J. Griffin

**Affiliations:** a Newcastle Cancer Centre , Northern Institute for Cancer Research , School of Chemistry , Newcastle University , Bedson Building , Newcastle Upon Tyne , NE1 7RU , UK . Email: bernard.golding@newcastle.ac.uk ; Email: duncan.miller@ncl.ac.uk ; Fax: +44 (0)191 2226929 ; Tel: +44 (0)191 2226647

## Abstract

Inhibitors of sulfatase-2 are putative anticancer agents, but the discovery of potent small molecules targeting this enzyme has proved challenging. Based on molecular modelling, two series of sulfatase-2 inhibitors have been developed with biphenyl and biphenyl ether scaffolds judiciously substituted with sulfamate, carboxylate and other polar groups (*e.g.* amino). Inhibition of aryl sulfatase A and B was also determined. The biphenyl ether derivatives were less selective for sulfatase-2 over aryl sulfatase B than the biphenyl series. All biphenyl ether derivatives inhibited aryl sulfatase A, whereas only amino derivatives inhibited aryl sulfatase B significantly. In the biphenyl series few derivatives exhibited activity against aryl sulfatase B. The trichloroethylsulfamate group was identified as a new pharmacophore enabling potent inhibition of all of the sulfatases studied.

## Abstract 2

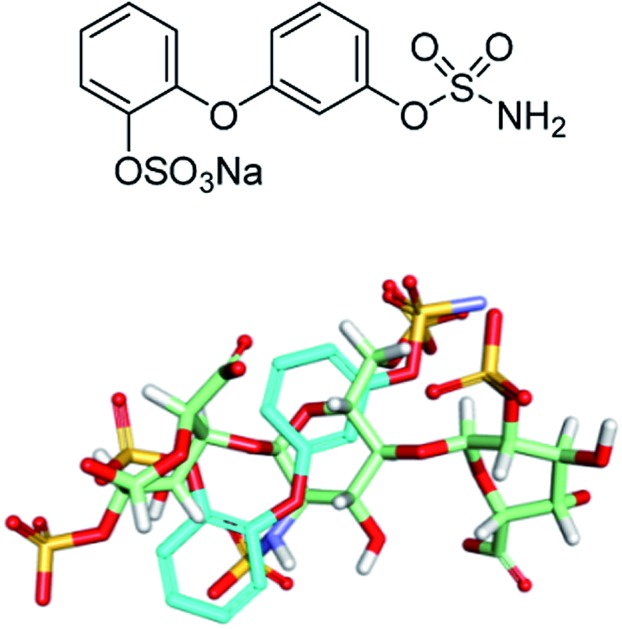
Two series of inhibitors of sulfatase 2, ARSA and ARSB were designed based on biphenyl and biphenyl ether scaffolds substituted with *e.g.* sulfamate and carboxylate groups.

## Introduction

Sulfatase-1 (Sulf-1) and sulfatase-2 (Sulf-2) catalyse the hydrolysis of the 6-*O*-sulfate group attached to glucosamine residues in heparan sulfate proteoglycans (HSPGs).^1^ The HSPG substrates consist of a protein core linked to heparan sulfate polysaccharide chains derived from repeating disaccharide units comprising a hexuronic acid (glucuronic acid or iduronic acid) and glucosamine. Removal of the 6-*O*-sulfate affects the mobilisation of heparan bound growth factors and cytokines,^2^ including fibroblast growth factor (FGF) and wingless-related integration site (wnt).^3^ The role of FGF in cell proliferation, invasion, migration and angiogenesis,^4^ and of wnt in cell growth and proliferation,^5^ implicate the sulfatases as potential targets for therapeutic intervention in certain diseases including pulmonary fibrosis^6^ and prostate,^7^ colorectal,^8^ ovarian, and breast cancers.^9^ Very few small molecule inhibitors of Sulf-1 and Sulf-2 have been reported.^10^,^11^ On re-synthesis, the purported activity against Sulf-2 of a group of monosaccharide sulfamate-based inhibitors could not be replicated.^12^ The nitrone ‘OKN-007’ is a suboptimal Sulf-2 inhibitor tool compound, having several proposed potential chemical and biological mechanisms of action in addition to Sulf-2 inhibition, including suppression of NO production, S-nitrosylation of critical proteins and inhibition of NF-κB activation.^13^ The difficulty in identifying small molecule inhibitors of Sulf-2 has led to the investigation of indirect methods to affect Sulf-2 through inhibition of the proteosomal machinery.^14^ However, in this paper we report new saccharide mimics that inhibit Sulf-2 directly. We have also identified the trichloroethylsulfamate group as a new pharmacophore for Sulf-2 inhibition. Among the compounds described are the first relatively potent small molecule inhibitors of Sulf-2 that will aid further biological studies and assist elucidation of the role of Sulf-2 in cancer.

## *De novo* inhibitor design

There is no crystal structure for Sulf-2 and a lack of either small molecule inhibitors or biological assay suitable for a high-throughput screening campaign. Given these constraints, the structure of heparan sulfate proteoglycans, which are the endogenous ligands of Sulf-2, was used as a template for the design of potential Sulf-2 inhibitors. With no solved structures of a HSPG, the principal structural motifs of HSPG reported to be substrates for Sulf-2 are the highly sulfated regions, which closely resemble heparin in structure.^15^ However, structures of heparin in solution have been solved by NMR (pdb code ; 1hpn).^16^ These were compared with structures of heparin bound to protein partners such as the NK1 growth factor (pdb code ; 1gmn), 3-*O*-sulfotransferase (pdb code ; 1t8u) and heparin lyase (pdb code ; 3ina). The bound form was found to resemble the solution α-helical form, with interactions to protein binding partners being dominated by contacts between the sulfate and carboxylate functionalities on the surface of the heparin helix. Negatively charged sulfate and carboxylate groups cover most of the accessible surface of the heparin polymer, and have been shown to regulate the binding of heparin to the enzyme 3-*O*-sulfotransferase.^16^,^17^ X-ray crystal structures of FGF-1 and FGF-2 in complex with heparin oligosaccharides also revealed the importance of the sulfate and carboxylate groups for binding.^18^ These studies demonstrated that interactions of the charged sulfate and carboxylate groups on the heparin oligosaccharide with basic amino acids on protein binding partners are the main contributors to tight and specific binding. Mimicking these ionic interactions was a key consideration for *de novo* design of potential non-saccharide based Sulf-2 inhibitors.

**Fig. 1:**
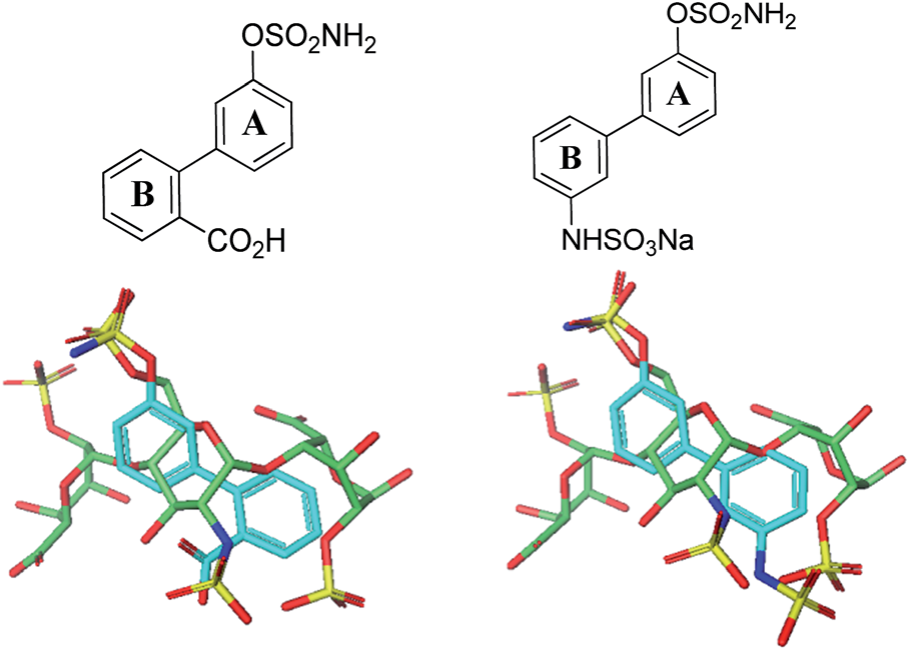
Representative overlays of biphenyl targets (cyan) with a heparin-derived trisaccharide template (green) created using the proprietary structural visualisation program MoViT (Pfizer).

**Fig. 2:**
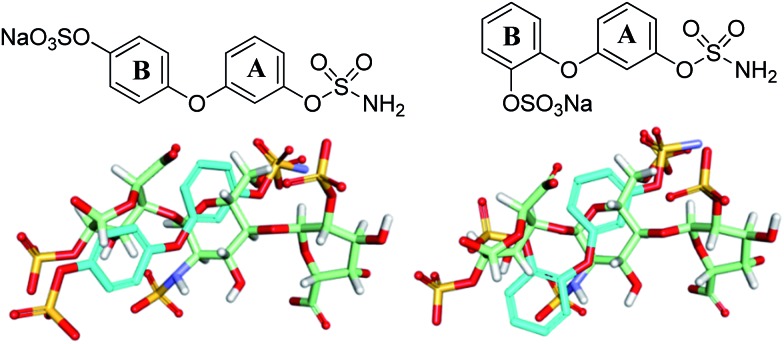
Representative overlays of biphenyl ethers (cyan) with a heparin-derived trisaccharide template (green).

Potential sulfamate inhibitors were designed with the aim of identifying hit compounds with improved Sulf-2 inhibition and physicochemical properties compared to the monosaccharides.^12^ The arylsulfamate group has been shown to be a privileged structure for the inhibition of sulfatases,^19^ although much of this work was focused on steroid sulfatase (STS). Potential Sulf-2 inhibitors were designed initially by overlaying a primary phenylsulfamate (A-ring) with the 6-*O* sulfate of glucosamine of a representative trisaccharide taken from the reducing end of the heparin solution structure (1hpn). Vectors where the phenyl ring could be substituted to allow incorporation of a second polar group that could overlay a surface polar group on the heparin helix were explored. Compounds from both biphenyl (Fig. 1) and biphenyl ether sulfamate (Fig. 2) series were energy minimised and found to enable such a superposition of polar functionality. A primary sulfamate group at the 3-position of the (A-ring) in the biphenyl series enabled substituents on the second phenyl ring to overlay the *N*-sulfate of glucosamine or the *O*-sulfate of iduronic acid in the template structure. In addition to these rationally designed derivatives, the biphenyl core was used to probe additional polar and ionic interactions through incorporation of polar functional groups at the *ortho*, *meta* and *para* positions of the B-ring. In the biphenyl ether sulfamate scaffold the oxygen linker allows different conformations with alternative positioning of groups on the B-ring relative to the A-ring.

## Synthesis of inhibitors

**Scheme 1:**
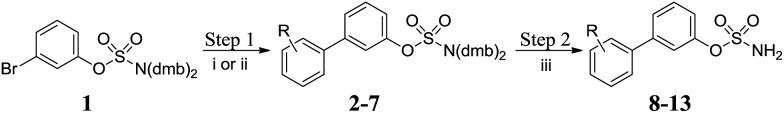
Synthesis of biphenyl sulfamates **8–13**. Reagents and conditions: (i) K_2_CO_3_, RC_6_H_4_B(OH)_2_, Pd(PPh_3_)_4_, MeCN, 120 °C, μW, 20 min; (ii) RC_6_H_4_B(pin), 2 M aq. Na_2_CO_3_, Pd(dppf)Cl_2_, dioxane, 80 °C, μW, 20 min; (iii) 10% TFA/DCM, RT, 2 h.

A number of highly efficient synthetic routes were implemented that should have wide applicability for sulfamate synthesis. Key to the synthesis of the desired templates was our previously reported sulfamate protecting group methodology using the *N*-2,4-dimethoxybenzyl (dmb) group,^20^ which was applied to the synthesis of libraries of biphenyl- and biaryl ether-sulfamates. Microwave-assisted Suzuki–Miyaura cross-coupling of **1** in the presence of potassium carbonate and Pd(PPh_3_)_4_ afforded **2** in 75% yield (method i, Scheme 1 and Table 1). Alternative Suzuki conditions (method ii, Scheme 1 and Table 1) were required for the preparation of **3–7**. All deprotections proceeded at room temperature in dilute TFA, resulting in high yields of the desired primary sulfamates **8–13**.

**Table 1:** Summary of yields for synthesis of biphenyl sulfamates

R	Step 1		Step 2
	Method	Yield	Yield
2-CO_2_Me	i	**2** 75%	**8** 88%
3-CO_2_H	ii	**3** 79%	**9** 82%
4-CO_2_H	ii	**4** 77%	**10** 90%
2-NH_2_	ii	**5** 82%	**11** 83%
3-NH_2_	ii	**6** 77%	**12** 82%
4-NH_2_	ii	**7** 72%	**13** 82%

**Scheme 2:**
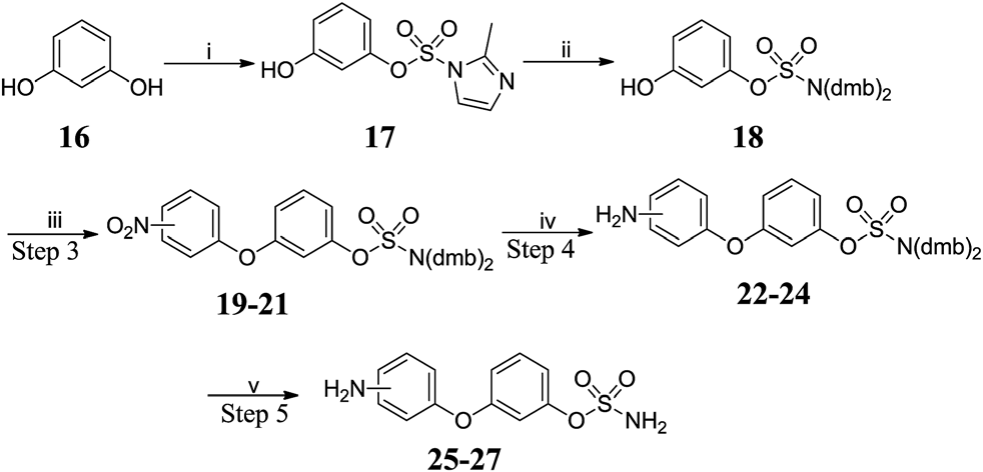
Synthesis of biaryl ether-sulfamates **25–27**. Reagents and conditions: (i) 1,1′-sulfonylbis(2-methyl-1*H*-imidazole), Cs_2_CO_3_, MeCN, 120 °C, μW, 15 min, 80%; (ii) (a) Me_3_O·BF_4_, DCM : THF (8 : 1), 0 °C to RT, 9 h; (b) bis(2,4-dimethoxy benzyl)amine, MeCN, 42 °C, 24 h, 60%; (iii) fluoronitrobenzene, K_2_CO_3_, DMF, 150 °C, μW, 20 min; (iv) H_2_, 10% Pd/C, MeOH : THF (3 : 1), RT, 24 h; (v) 10% TFA/DCM, RT, 2 h.

The preparation of amino-substituted biaryl ether-sulfamates **25–27** started from resorcinol **16** (Scheme 2 and Table 2). The use of high dilution (0.08 M) and low temperature prevented side-reactions during the methylation/imidazolium displacement step. S_N_Ar reaction of fluoronitrobenzenes with **18** gave biphenyl ether intermediates **19–21**, which were reduced *via* palladium-catalysed flow hydrogenation. Deprotection of **22–24** gave high yields of the desired primary sulfamates **25–27**.

**Table 2:** Summary of yields for synthesis of biaryl ether-sulfamates

Position	Step 3	Step 4	Step 5
2′-	**19** 80%	**22** 85%	**25** 90%
3′-	**20** 75%	**23** 87%	**26** 82%
4′-	**21** 85%	**24** 86%	**27** 65%

**Scheme 3:**
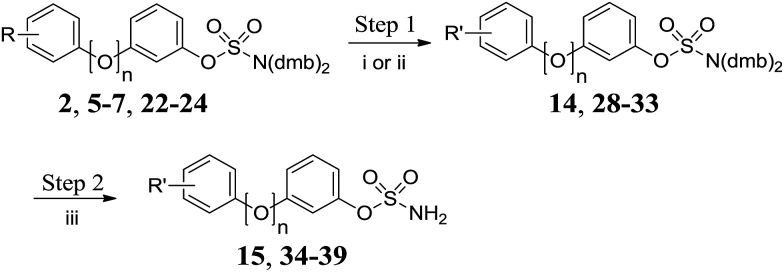
Functionalisation/deprotection of protected biphenyl sulfamates. Reagents and conditions: (i) LiOH, H_2_O/THF, 60 °C, 24 h, 80%; (ii) Ac_2_O, NEt_3_, DCM, RT, 24 h; (iii) 10% TFA/DCM, RT, 2 h.

Methyl ester **2** was hydrolysed under basic conditions and the resulting carboxylic acid **14** was deprotected to give **15** (Scheme 3 and Table 3). Acetylation of aniline derivatives **5–7** and **22–24** afforded acetamidobiphenyls **28–33**, which were deprotected to give **34–39**.

**Table 3:** Summary of yields for functionalisation/deprotection of protected biphenyl sulfamates

R	R′	*n*	Step 1		Step 2
			Method	Yield	Yield
2-CO_2_Me	2-CO_2_H	0	i	**14** 80%	**15** 89%
2-NH_2_	2-NHAc	0	ii	**28** 87%	**34** 86%
3-NH_2_	3-NHAc	0	ii	**29** 89%	**35** 91%
4-NH_2_	4-NHAc	0	ii	**30** 88%	**36** 84%
2-NH_2_	2-NHAc	1	ii	**31** 87%	**37** 85%
3-NH_2_	3-NHAc	1	ii	**32** 88%	**38** 94%
4-NH_2_	4-NHAc	1	ii	**33** 86%	**39** 92%

**Scheme 4:**
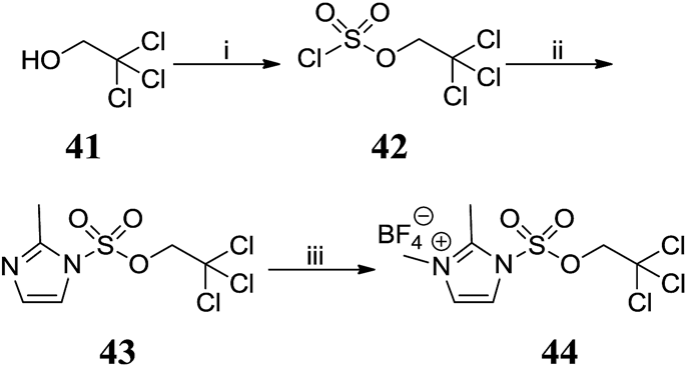
Synthesis of 2,3-dimethyl-1-((2,2,2-trichloroethoxy)-sulfonyl)-1*H*-imidazol-3-ium tetrafluoroborate **44**. Reagents and conditions: (i) SO_2_Cl_2_, pyridine, Et_2_O, –78 °C, 4 h, 83%; (ii) 2-methylimidazole, THF, 0 °C to RT, 16 h, 95%; (iii) Me_3_O·BF_4_, DCM, 0 °C to RT, 20 h, 86%.

Attempted preparation of the 2′-sulfamic acid derivative by reaction of aniline **5** with sulfur trioxide-pyridine complex was unsuccessful. Taylor *et al.* reported the 2,2,2-trichloroethyl (TCE) group as an effective protecting group for arylsulfate esters^21^ and this methodology was adapted to the synthesis of protected sulfamic acids. TCE chlorosulfate **42** was obtained by reacting sulfuryl chloride with one equivalent of 2,2,2-trichloroethanol **41** (Scheme 4). Reaction of **42** with 2-methylimidazole gave 2-methylimidazole-1-sulfonate **43**, which was methylated with Meerwein's salt to give **44**.

**Scheme 5:**
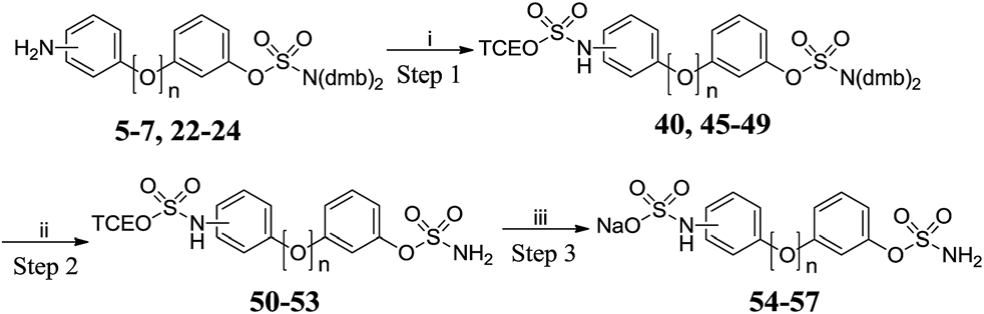
Synthesis of amino-sulfate derivatives **54–57**. Reagents and conditions: (i) **44**, MeCN, 120 °C, μW, 20 min; (ii) 10% TFA/DCM, RT, 2 h; (iii) (a) Zn powder, MeOH, acetate buffer pH 4.65, 60 °C, 2 h; (b) Dowex 50W8X2 Na^+^ form, H_2_O.

Reacting the appropriate anilines with 3 molar equivalents of **44** under microwave irradiation in acetonitrile at 120 °C for 20 min afforded the *meta*- (**45** and **48**) and *para*- (**46** and **49**) derivatives, but poor conversion was observed with *ortho*-anilines **5** and **22** (Scheme 5 and Table 4). The trichloroethyl group was stable under the acidic conditions used for sulfamate deprotection. Cleavage of the trichloroethyl group was achieved using zinc in a mixture of methanol and acetate buffer with no desulfamoylation. The resulting sulfamic acids were converted to sodium salts using ion exchange chromatography, affording targets **54–57** in high yields.

**Table 4:** Summary of yields for the synthesis of amino-sulfate derivatives

Position	*n*	Step 1	Step 2	Step 3
2′-	0	**40** 0%	—	—
3′-	0	**45** 75%	**50** 86%	**54** 84%
4′-	0	**46** 82%	**51** 91%	**55** 80%
2′-	1	**47** 15%	—	—
3′-	1	**48** 78%	**52** 86%	**56** 75%
4′-	1	**49** 90%	**53** 89%	**57** 70%

**Scheme 6:**
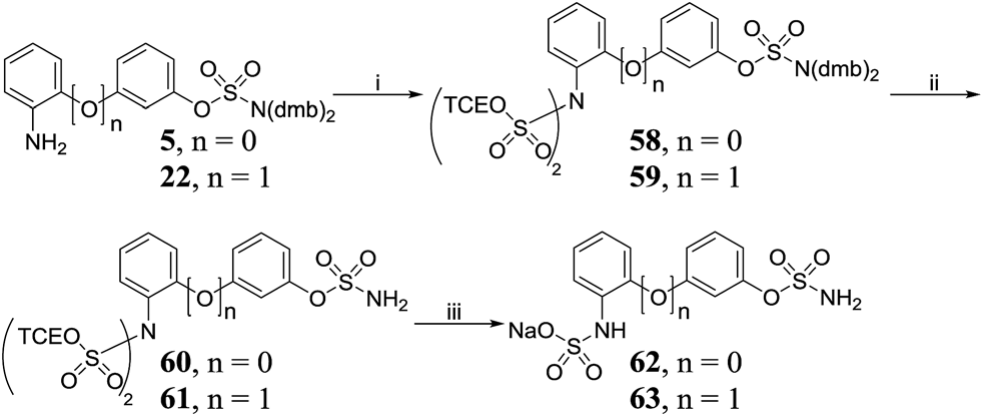
Synthesis of amino-sulfate derivatives **62**, **63**. Reagents and conditions: (i) **44**, NEt_3_, DMAP, THF, 0 °C to RT, 24 h, *n* = 0 70%, *n* = 1 55%; (ii) 10% TFA/DCM, RT, 2 h, *n* = 0 90%, *n* = 1 86%; (iii) Zn powder, MeOH, acetate buffer pH 4.65, AcOH, RT, 24 h; Dowex 50W8X2 Na^+^ form, H_2_O, *n* = 0 50%, *n* = 1 33%.

Protection of 2-aminobiphenyls **5** and **22** with TCE chlorosulfate **44** produced bis-*N*,*N*-sulfated aminobiphenyls **58** and **59**, respectively (Scheme 6), which were deprotected to afford **60** and **61**. A one-pot deprotection/mono-sulfate hydrolysis of **60** and **61** led to the isolation of **62** and **63**.

**Scheme 7:**
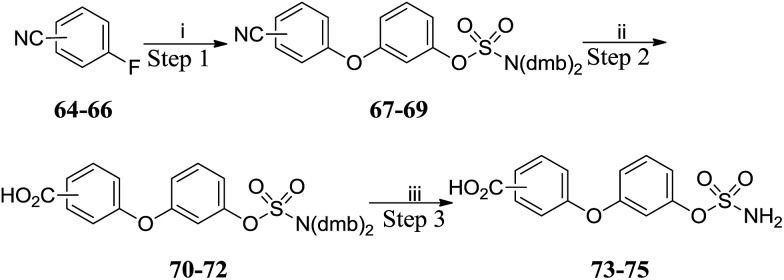
Synthesis of benzoic acid derivatives **73–75**. Reagents and conditions: (i) **18**, K_2_CO_3_, DMF, 150 °C, μW, 20 min; (ii) 2 M aq. NaOH, dioxane, 130 °C, μW, 2 h; (iii) 10% TFA/DCM, RT, 2 h.

S_N_Ar reactions with fluorobenzonitriles **64–66** gave cyanobiphenyl ethers **67–69** which were hydrolysed to the corresponding benzoic acids **70–72** (Scheme 7 and Table 5). Acidic deprotection of the sulfamate moiety afforded targets **73–75**. Thus, sets of substituted biphenyl and biphenyl ether sulfamates were prepared. Compounds **76–82** (Table 6) bearing a single substituent on the aromatic core were also prepared by analogous routes (see ESI data†).

**Table 5:** Summary of yields for synthesis of benzoic acid derivatives

Position	Step 1	Step 2	Step 3
2′-	**67** 81%	**70** 45%	**73** 90%
3′-	**68** 61%	**71** 80%	**74** 94%
4′-	**69** 72%	**72** 65%	**75** 92%

**Table 6:** Monosubstituted phenyl and biphenyl derivatives

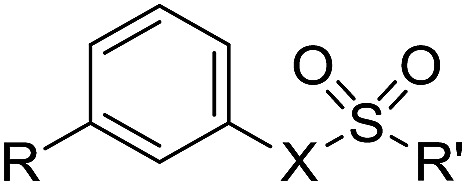			
Cmpd	R	X	R′
**76**	Ph	O	NH_2_
**77**	Ph	O	NMe_2_
**78**	OPh	O	NH_2_
**79**	Ph	NH	OCH_2_CCl_3_
**80**	OPh	NH	OCH_2_CCl_3_
**81**	Ph	O	OCH_2_CCl_3_
**82**	Ph	NH	OCH_2_CF_3_
**83**	H	NH	OCH_2_CCl_3_

## Biological evaluation

Compounds were assayed for their ability to inhibit the Sulf-2-catalysed desulfation of 4-methylumbelliferyl sulfate (4-MUS) to the fluorescent 4-methylumbelliferone (MU). To determine their sulfatase selectivity, inhibition of aryl sulfatases A (ARSA) and B (ARSB) was also assessed. To date no suitable benchmark inhibitors of these sulfatase have been reported. In the biphenyl series (Table 7A), only the trichloroethylsulfamates (**50** and **51**) exhibited high Sulf-2 inhibition giving almost complete inhibition of sulfatase activity at 1 mM concentration. The position and nature of the substituent on the B ring had a pronounced effect on the sulfatase inhibition. In the majority of cases the presence of a substituent on the B-ring was detrimental to Sulf-2 inhibition when compared with unsubstituted biphenyl sulfamate **76**. Aminosulfates **54** and **62** and carboxylic acid **15** retained potency. Surprisingly, the synthetic trichloroethylsulfamate intermediates **50** and **51** exhibited potent inhibition. Compounds **8**, **10**, **15**, **50** and **51** were also relatively potent ARSA inhibitors. The tertiary dimethylsulfamate **77** exhibited no Sulf-2 inhibition (0% inh @ 1 mM). Similar SAR was observed with the 3-phenoxyphenyl template (Table 7B) with the trichloroethylsulfamates **52** and **53** again proving to be superior inhibitors against all sulfatases tested. Aminosulfates **56** and **63** were the best Sulf-2 inhibitors lacking a trichloroethylsulfamate group. In summary, the design strategy of introducing polar groups to mimic polar groups of the endogenous substrate did not give the anticipated improvement in Sulf-2 inhibition, whereas the presence of a relatively large, lipophilic trichloroethylsulfamate group on the B-ring in both templates provided superior inhibitors.

**Table 7:** Sulfatase inhibition data for biphenylsulfamates. Data are means of two or more determinations. n.d. = not determined

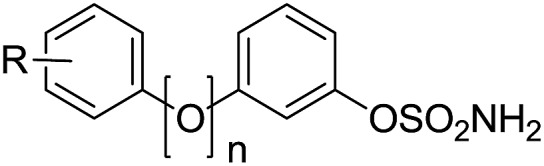				
Cpd	R	Sulf-2% inh @ 1 mM	ARSA% inh @ 1 mM	ARSB% inh @ 1 mM
**A: *n* = 0**				
**76**	H	38	14	65
**8**	2′-CO_2_Me	20	86	0
**15**	2′-CO_2_H	31	98	0
**9**	3′-CO_2_H	12	59	0
**10**	4′-CO_2_H	22	85	0
**11**	2′-NH_2_	21	0	0
**12**	3′-NH_2_	20	0	0
**13**	4′-NH_2_	6	61	0
**34**	2′-NHAc	4	33	0
**35**	3′-NHAc	8	52	33
**36**	4′-NHAc	3	0	0
**62**	2′-NHSO_3_Na	37	26	0
**54**	3′-NHSO_3_Na	31	0	0
**55**	4′-NHSO_3_Na	4	0	0
**60**	2′-N(SO_3_CH_2_CCl_3_)_2_	10	63	0
**50**	3′-NHSO_3_CH_2_CCl_3_	99	92	67
**51**	4′-NHSO_3_CH_2_CCl_3_	96	99	68

**B: *n* = 1**				
**78**	H	32	n.d.	n.d.
**73**	2′-CO_2_H	10	44	0
**74**	3′-CO_2_H	30	49	0
**75**	4′-CO_2_H	23	33	0
**25**	2′-NH_2_	28	75	17
**26**	3′-NH_2_	31	75	0
**27**	4′-NH_2_	14	75	0
**37**	2′-NHAc	11	29	48
**38**	3′-NHAc	10	57	0
**39**	4′-NHAc	14	45	0
**63**	2′-NHSO_3_Na	56	54	93
**56**	3′-NHSO_3_Na	58	95	85
**57**	4′-NHSO_3_Na	13	34	77
**61**	2′-N(SO_3_CH_2_CCl_3_)_2_	18	82	40
**52**	3′-NHSO_3_CH_2_CCl_3_	100	100	96
**53**	4′-NHSO_3_CH_2_CCl_3_	98	96	86

To understand the origin of sulfatase inhibition by compounds **50–53**, compounds **79** and **80**, lacking a primary sulfamate, were prepared and found to be also potent Sulf-2 inhibitors (Table 8), indicating that the primary sulfamate group is not essential for sulfatase inhibition with these templates. Compound **79** exhibited some selectivity over ARSA and ARSB, whereas compound **80** was a good inhibitor for all of the sulfatases. Compound **83**, the simple phenyl trichloroethylsulfamate derivative did not retain Sulf-2 inhibitory activity, suggesting that the trichloroethylsulfamate group is not sufficient *per se* to impart sulfatase inhibition and is not conferring pan-sulfatase activity through a non-specific mechanism of action. Compound **83** did, however, possess inhibitory activity against both ARSA and ARSB. Trichloroethylsulfate **81** and trifluoroethylsulfamate **82** exhibited no Sulf-2 inhibition, but retained activity against ARSA and ARSB. Thus, it appears that the trichloroethylsulfamate group is a new motif for Sulf-2 inhibition, and that, in combination with lipophilic groups, inhibitors of Sulf-2 lacking a primary sulfamate moiety can be developed.

**Table 8:** Sulfatase inhibition data for analogues lacking a primary sulfamate group. Data are means of two or more determinations

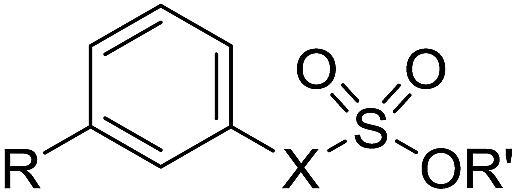						
Cpd	R	X	R′	Sulf-2% inh @ 1 mM	ARSA% inh @ 1 mM	ARSB% inh @ 1 mM
**83**	H	NH	CH_2_CCl_3_	3	63	68
**79**	Ph	NH	CH_2_CCl_3_	100	0	61
**80**	OPh	NH	CH_2_CCl_3_	99	74	99
**81**	Ph	O	CH_2_CCl_3_	1	70	85
**82**	Ph	NH	CH_2_CF_3_	5	78	92

Sulf-2 IC_50_ values were determined for the most potent inhibitors (Table 9). Compound **50** had the highest Sulf-2 inhibitory activity with an IC_50_ value of 167 μM. The ARSA and ARSB inhibitory activity of all compounds tested proved to be higher than their Sulf-2 inhibitory activity, providing the first reported inhibitors of ARSA and ARSB with IC_50_ < 1 mM. Following screening at 1 mM, ARSA inhibition by **80** was evaluated over the concentration range 0.025–1 mM. Concentration-dependent inhibition was not observed and hence an IC_50_ value could not be determined. Aryl sulfamate-based inhibitors of steroid sulfatase have demonstrated irreversible inhibition profiles.^15^ Further studies will be required to determine if the aryl sulfamates such as **50**, and trichloroethylsulfamates such as **80** behave as irreversible sulfatase inhibitors.

**Table 9:** Sulfatase IC_50_ data for inhibitors **50–51**, **53**, **79–80**. IC_50_ values were calculated using non-linear regression analysis for 7 different inhibitor concentrations each performed in duplicate to give the mean ± SEM

Compound	Sulf-2 IC_50_ (μM)	ARSA IC_50_ (μM)	ARSB IC_50_ (μM)
**50**	167 ± 5	55 ± 11	130 ± 6
**51**	566 ± 28	91 ± 4	101 ± 8
**53**	390 ± 11	108 ± 2	116 ± 10
**79**	254 ± 3	No inhibition	30 ± 11
**80**	298 ± 3	nd	34 ± 9

## Conclusions

Two new series of sulfatase inhibitors have been developed based on biphenyl and biphenyl ether templates, with highly effective synthetic procedures providing access to a variety of sulfamates and aminosulfates. The trichloroethylsulfamates **50–53**, **79** and **80** exhibited favourable Sulf-2 inhibition. The trichloroethylsulfamate group has therefore been identified as a new pharmacophore for sulfatase inhibition. Further studies will be required to determine the origin of sulfatase inhibition by aryl trichloroethylsulfamates. The most potent Sulf-2 inhibitors lacking a trichloroethylsulfamate group were biphenyl ether aminosulfates **56** and **63**. The diphenyl ether derivatives also proved to be moderate to good inhibitors of ARSA, whereas significant inhibition of ARSB was only observed with substituted amino derivatives. This trend was not retained in the biphenyl series with few derivatives exhibiting activity against ARSB. However, the first inhibitors of ARSA and ARSB with IC_50_ < 1 mM are reported. Additional SAR studies are underway to improve Sulf-2 potency and to delineate the factors affecting selectivity.

## Author contributions

DCM, BTG and RJG designed the inhibitors; BTG, DCM and DRN wrote the manuscript; TR, AB (Bertoli) and AB (Brennan) performed the chemical synthesis supervised by CC, BTG, RJG and DCM; SFA and GSB performed the biological assays supervised by DRN and HLR.

## Supplementary Material

Supplementary information

## References

[cit1] Morimoto-Tomita M., Uchimura K., Werb Z., Hemmerich S., Rosen S. D. (2002). J. Biol. Chem..

[cit2] Uchimura K., Morimoto-Tomita M., Bistrup A., Li J., Lyon M., Gallagher J., Werb Z., Rosen S. D. (2006). BMC Biochem..

[cit3] Nawroth R., van Zante A., Cervantes S., McManus M., Hebrok M., Rosen S. D. (2007). PLoS One.

[cit4] Turner N., Grose R. (2010). Nat. Rev. Cancer.

[cit5] Klaus A., Birchmeier W. (2008). Nat. Rev. Cancer.

[cit6] Yue X., Lu J., Auduong L., Sides M. D., Lasky J. A. (2013). Glycobiology.

[cit7] Vicente C. M., Lima M. A., Nader H. B., Toma L. (2015). J. Exp. Clin. Cancer Res..

[cit8] Vicente C. M., Lima M. A., Yates E. A., Nader H. B., Toma L. (2015). Mol. Cancer Res..

[cit9] Khurana A., Beleford D., He X., Chien J., Shridhar V. (2013). Am. J. Cancer Res..

[cit10] Schelwies M., Brinson D., Otsuki S., Hong Y.-H., Lotz M. K., Wong C.-H., Hanson S. R. (2010). ChemBioChem.

[cit11] Zheng X., Gai X., Han S., Moser C. D., Hu C., Shire A. M., Floyd R. A., Roberts L. R. (2013). Genes, Chromosomes Cancer.

[cit12] Miller D. C., Carbain B., Beale G. S., Alhasan S. F., Reeves H. L., Baisch U., Newell D. R., Golding B. T., Griffin R. J. (2015). Org. Biomol. Chem..

[cit13] Floyd R. A., Chandru H. K., He T., Towner R. (2011). Anti-Cancer Agents Med. Chem..

[cit14] Khurana A., Jung-beom D., He X., Kim S., Busby R. C., Lorenzon L., Shridhar V. (2013). Clin. Exp. Metastasis.

[cit15] Saad O. M., Ebel H., Uchimura K., Rosen S. D., Bertozzi C. R., Leary J. A. (2005). Glycobiology.

[cit16] Mulloy B., Forster M. J. (2000). Glycobiology.

[cit17] Moon R. T., Kohn A. D., Ferrari G. V. D., Kaykas A. (2004). Nat. Rev. Genet..

[cit18] Capila I., Linhardt R. J. (2002). Angew. Chem., Int. Ed..

[cit19] Thomas M. P., Potter B. V. L. (2015). J. Med. Chem..

[cit20] Reuillon T., Bertoli A., Griffin R. J., Miller D. C., Golding B. T. (2012). Org. Biomol. Chem..

[cit21] Liu Y., Lien I. F. F., Ruttgaizer S., Dove P., Taylor S. D. (2004). Org. Lett..

