# Supplementary material for: Design and synthesis of biphenyl and biphenyl ether inhibitors of sulfatases
Source: Chem Sci. 2016 Jan 11;7(4):2821–6. doi: 10.1039/c5sc03612g (PMC5477036; doi:10.1039/c5sc03612g)
Supplement: Supplementary file 1 [file SC-007-C5SC03612G-s001.pdf]

Electronic Supplementary Material (ESI) for Chemical Science.
This journal is © The Royal Society of Chemistry 2016

Supplementary Information

for

Design and Synthesis of Biphenyl and Biphenyl Ether Inhibitors of Sulfatases

Tristan Reuillon, Sari F. Alhasan, Gary S. Beale, Annalisa Bertoli, Alfie Brennan, Celine Cano, Helen L. Reeves, David R Newell, Bernard T. Golding,* Duncan C. Miller,* and Roger J. Griffin✝

Table of contents

Sulf-2 assay protocol 5

ARSA and ARSB assay protocols 5

General synthetic procedures 10

Synthetic procedures 12

3-Bromophenyl bis(2,4-dimethoxybenzyl)sulfamate, (1) 12
Methyl 3'-((*N*,*N*-bis(2,4-dimethoxybenzyl)sulfamoyl)oxy)-[1,1'-biphenyl]-2-carboxylate, (2) 13
3'-((*N*,*N*-Bis(2,4-dimethoxybenzyl)sulfamoyl)oxy)-[1,1'-biphenyl]-3-carboxylic acid, (3) 14
3'-((*N*,*N*-Bis(2,4-dimethoxybenzyl)sulfamoyl)oxy)-[1,1'-biphenyl]-4-carboxylic acid, (4) 15
2'-Amino-[1,1'-biphenyl]-3-yl bis(2,4-dimethoxybenzyl)sulfamate, (5) 16
3'-Amino-[1,1'-biphenyl]-3-yl bis(2,4-dimethoxybenzyl)sulfamate, (6) 16
4'-Amino-[1,1'-biphenyl]-3-yl bis(2,4-dimethoxybenzyl)sulfamate, (7) 17
Methyl 3'-(sulfamoyloxy)-[1,1'-biphenyl]-2-carboxylate, (8) 18
3'-(Sulfamoyloxy)-[1,1'-biphenyl]-3-carboxylic acid, (9) 19
3'-(Sulfamoyloxy)-[1,1'-biphenyl]-4-carboxylic acid, (10) 19
2'-Amino-[1,1'-biphenyl]-3-yl sulfamate, (11) 20
3'-Amino-[1,1'-biphenyl]-3-yl sulfamate, (12) 20
4'-Amino-[1,1'-biphenyl]-3-yl sulfamate, (13) 21
3'-((*N*,*N*-Bis(2,4-dimethoxybenzyl)sulfamoyl)oxy)-[1,1'-biphenyl]-2-carboxylic acid, (14) 22
3'-(Sulfamoyloxy)-[1,1'-biphenyl]-2-carboxylic acid, (15) 23
3-Hydroxyphenyl 2-methyl-1*H*-imidazole-1-sulfonate, (17) 23
3-Hydroxyphenyl bis(2,4-dimethoxybenzyl)sulfamate, (18) 24
3-(2-Nitrophenoxy)phenyl bis(2,4-dimethoxybenzyl)sulfamate, (19) 25
3-(3-Nitrophenoxy)phenyl bis(2,4-dimethoxybenzyl)sulfamate, (20) 26
3-(4-Nitrophenoxy)phenyl bis(2,4-dimethoxybenzyl)sulfamate, (21) 26
3-(2-Aminophenoxy)phenyl bis(2,4-dimethoxybenzyl)sulfamate, (22) 27
3-(3-Aminophenoxy)phenyl bis(2,4-dimethoxybenzyl)sulfamate, (23) 28
3-(4-Aminophenoxy)phenyl bis(2,4-dimethoxybenzyl)sulfamate, (24) 29
3-(2-Aminophenoxy)phenyl sulfamate, (25) 29
3-(3-Aminophenoxy)phenyl sulfamate, (26) 30
3-(4-Aminophenoxy)phenyl sulfamate, (27) 31
2'-Acetamido-[1,1'-biphenyl]-3-yl bis(2,4-dimethoxybenzyl)sulfamate, (28) 31
3'-Acetamido-[1,1'-biphenyl]-3-yl bis(2,4-dimethoxybenzyl)sulfamate, (29) 32
4'-Acetamido-[1,1'-biphenyl]-3-yl bis(2,4-dimethoxybenzyl)sulfamate, (30) 33
3-(2-Acetamidophenoxy)phenyl bis(2,4-dimethoxybenzyl)sulfamate, (31) 34

3-(3-Acetamidophenoxy)phenyl bis(2,4-dimethoxybenzyl)sulfamate, (32)............35
3-(4-Acetamidophenoxy)phenyl bis(2,4-dimethoxybenzyl)sulfamate, (33)............35
2'-Acetamido-[1,1'-biphenyl]-3-yl sulfamate, (34) 36
3'-Acetamido-[1,1'-biphenyl]-3-yl sulfamate, (35) 37
4'-Acetamido-[1,1'-biphenyl]-3-yl sulfamate, (36) 37
3-(2-Acetamidophenoxy)phenyl sulfamate, (37) 38
3-(3-Acetamidophenoxy)phenyl sulfamate, (38) 39
3-(4-Acetamidophenoxy)phenyl sulfamate, (39) 39
2,2,2-Trichlroethyl chlorosulfate, (41) 40
2,2,2-Trichloroethyl 2-methyl-1*H*-imidazole-1-sulfonate, (42)..............................40
2,3-Dimethyl-1-((2,2,2-trichloroethoxy)sulfonyl)-1*H*-imidazol-3-ium tetrafluoroborate, (44) 41
3'-(((2,2,2-Trichloroethoxy)sulfonyl)amino)-[1,1'-biphenyl]-3-yl bis(2,4-dimethoxybenzyl)sulfamate, (45) 42
4'-(((2,2,2-Trichloroethoxy)sulfonyl)amino)-[1,1'-biphenyl]-3-yl bis(2,4-dimethoxy benzyl)sulfamate, (46)...42
3-(2-(((2,2,2-Trichloroethoxy)sulfonyl)amino)phenoxy)phenyl bis(2,4-dimethoxybenzyl) sulfamate, (47) 43
3-(3-(((2,2,2-Trichloroethoxy)sulfonyl)amino)phenoxy)phenyl bis(2,4-dimethoxybenzyl) sulfamate, (48) 44
3-(4-(((2,2,2-Trichloroethoxy)sulfonyl)amino)phenoxy)phenyl bis(2,4-dimethoxybenzyl) sulfamate, (49) 45
2,2,2-Trichloroethyl (3'-(sulfamoyloxy)-[1,1'-biphenyl]-3-yl)sulfamate, (50)........46
2,2,2-Trichloroethyl (3'-(sulfamoyloxy)-[1,1'-biphenyl]-4-yl)sulfamate, (51)........46
2,2,2-Trichloroethyl (3-(3-(sulfamoyloxy)phenoxy)phenyl)sulfamate, (52)...........47
2,2,2-Trichloroethyl (4-(3-(sulfamoyloxy)phenoxy)phenyl)sulfamate, (53)...........48
Sodium (3'-(sulfamoyloxy)-[1,1'-biphenyl]-3-yl)sulfamate, (54)............................48
Sodium (3'-(sulfamoyloxy)-[1,1'-biphenyl]-4-yl)sulfamate, (55)............................49
Sodium (3-(3-(sulfamoyloxy)phenoxy)phenyl)sulfamate, (56)...............................49
Sodium (4-(3-(sulfamoyloxy)phenoxy)phenyl)sulfamate, (57)...............................50
2'-(bis((2,2,2-Trichloroethoxy)sulfonyl)amino)-[1,1'-biphenyl]-3-yl bis(2,4-dimethoxy benzyl)sulfamate, (58) 51
O,*O*-bis(2,2,2-Trichloroethyl) (2-(3-((*N*,*N*-bis(2,4-dimethoxybenzyl)sulfamoyl)oxy)phenoxy)phenyl)iminodisulfate, (59).................52
2,2,2-Trichloroethyl (3'-(sulfamoyloxy)-[1,1'-biphenyl]-2-yl)((2,2,2-trichloroethoxy) sulfonyl)sulfamate, (60) 53
O,*O*-bis(2,2,2-Trichloroethyl) (2-(3-(sulfamoyloxy)phenoxy)phenyl) iminodisulfate, (61) 53
Sodium (3'-(sulfamoyloxy)-[1,1'-biphenyl]-2-yl)sulfamate, (62)............................54
Sodium (2-(3-(sulfamoyloxy)phenoxy)phenyl)sulfamate, (63)...............................55
3-(2-Cyanophenoxy)phenyl bis(2,4-dimethoxybenzyl)sulfamate, (67)...................56
3-(3-Cyanophenoxy)phenyl bis(2,4-dimethoxybenzyl)sulfamate, (68)...................57
3-(4-Cyanophenoxy)phenyl bis(2,4-dimethoxybenzyl)sulfamate, (69)...................57
2-(3-((*N*,*N*-bis(2,4-Dimethoxybenzyl)sulfamoyl)oxy)phenoxy)benzoic acid, (70).58

3-(3-((*N*,*N*-bis(2,4-Dimethoxybenzyl)sulfamoyl)oxy)phenoxy)benzoic acid, (71).59
4-(3-((*N*,*N*-bis(2,4-Dimethoxybenzyl)sulfamoyl)oxy)phenoxy)benzoic acid, (72).60
2-(3-(Sulfamoyloxy)phenoxy)benzoic acid, (73)..61
3-(3-(Sulfamoyloxy)phenoxy)benzoic acid, (74)..62
4-(3-(Sulfamoyloxy)phenoxy)benzoic acid, (75)..62
3-Phenylphenyl sulfamate (76) 63
[1,1'-Biphenyl]-3-yl dimethylsulfamate (77)..63
3-Phenoxyphenyl sulfamate, (78) 64
2,2,2-Trichloroethyl (3-phenoxyphenyl)sulfamate (80) 65
[1,1'-biphenyl]-3-yl (2,2,2-trichloroethyl) sulfate, (81) 66
2,2,2-Trifluoroethyl [1,1'-biphenyl]-3-ylsulfamate (82) 66
2,2,2-Trichloroethyl phenylsulfamate (83) 67
1,1'-sulfonylbis(2-methyl-1*H*-imidazole), (S2)...67
3-Bromophenyl 2-methyl-1*H*-imidazole-1-sulfonate, (S4) 68
1-((3-Bromophenoxy)sulfonyl)-2,3-dimethyl-1*H*-imidazol-3-ium tetrafluoroborate, (S5)...69
2,2,2-Trifluoroethyl chlorosulfate (S6) 69
2,2,2-Trifluoroethyl 2-methyl-1*H*-imidazole-1-sulfonate (S7)...............................70
2,3-Dimethyl-1-((2,2,2-trifluoroethoxy)sulfonyl)-1*H*-imidazol-3-ium tetrafluoroborate (S8) 70
Sulfamoyl chloride, (S9) 71

1 – Biological assay protocols

Sulf-2 assay protocol

Compounds were screened using 4-MUS as a substrate for Sulf-2 according to a protocol described by Morimoto-Tomita *et al.*[1] Briefly 293T cells were transiently transfected with pcDNA3.1/Myc-His(-)-HSulf-2 DNA (Addgene) and TransIT-LT1 Transfection Reagent (Mirus) using a transfection mixture at the ratio 1:3 (μg DNA: μL transfection reagent) in Opti-MEM I reduced serum medium (Gibco). Conditioned medium containing Sulf-2 was collected after 3 days and bound to HIS-Select Nickel affinity gel (Sigma) overnight at 4 °C. Beads were washed three times with washing buffer containing 50 mM HEPES (pH 7.5), 300 mM NaCl, 0.05% Tween 20, followed by washing once with washing buffer containing no tween. Beads were suspended in 50 mM Hepes (pH 7.5) and used in inhibition assays. 20 μL of bead slurry was incubated with 1 mM compound (in DMSO) plus 10X reaction buffer (500 mM HEPES pH 7.5, 100 mM $CaCl_2$) for 1 h at 37 °C. The reaction was started by the addition of 20 μL of 20 mM 4-MUS (final concentration of 8 mM) and incubated at 37 °C for 1 h. The reaction was stopped with 100 μL 1 M Tris buffer (pH 10.4) and read at 460 nm following excitation at 355 nm in FLUOstar Omega plate reader (BMG Labtech) using Omega data analysis software.

ARSA and ARSB assay protocols

Compounds were screened in a 96-well black plate (Sterilin) using 4-MUS as a substrate, using 50 μL reaction mixture containing 40 ng of the commercially available enzymes (ARSA or ARSB from R & D Systems), 50 mM HEPES (pH = 4.5), 10 mM $CaCl_2$, 1 mM test compound (dissolved in DMSO; final concentration of DMSO in reaction = 2%), and H_2O (45 μL). The assay mixture was incubated for 1 h at 37 °C, followed by addition of 5 μL of 4-MUS (Km = 1.6 mM for ARSA and 612 μM for ARSB), and incubation for a further 1 h at 37 °C. The reaction was stopped with 100 μL of 1 M Tris (pH = 10.5) and read at 460 nm following excitation at 355 nm in FLUOstar Omega plate reader (BMG Labtech) using Omega data analysis software.

2- Supplemental synthetic schemes and tables.

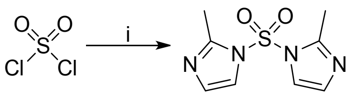

S1 **S2**

Scheme S1: *Reagents and conditions:* (i) 2-methylimidazole, DCM, 0 °C to RT, 24 h, 73%.

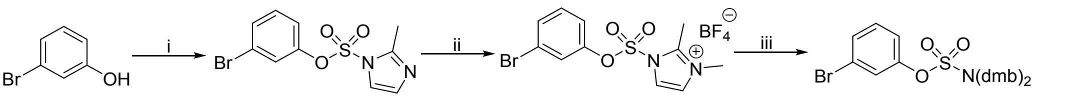

S3 **S4** **S5** **1**

Scheme S2: *Reagents and conditions:* (i) **S2**, Cs_2CO_3, MeCN, 120 °C, μW, 15 min, 76%; (ii) $Me_3O·BF_4$, DCM, 0 °C to RT, 8 h, 85%; (iii) bis(2,4-dimethoxybenzyl)amine, MeCN, RT, 16 h, 71%.

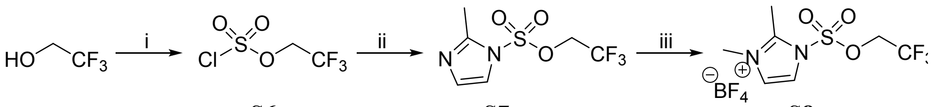

S6 **S7** **S8**

Scheme S3: *Reagents and conditions:* (i) SO_2Cl_2, pyridine, Et_2O, -78 °C, 4 h, 47%; (ii) 2-methylimidazole, THF, 0 °C to RT, 16 h, 77%; (iii) $Me_3O.BF_4$, DCM, 0 °C to RT, 20 h, 76%.

Table S1: *Reagents and conditions:* (i) $ClSO_2NH_2$ (**S9**) (≈ 1.8 M in MeCN), DMA, 0 °C to RT, 20 h; (ii) Cs_2CO_3, $ClSO_2NMe_2$, MeCN, RT, 65%; (iii) DMAP, NEt_3, **42**, THF (17 + 8.5 mL), 0 °C to RT, 24 h, 96%.

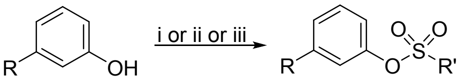

76, 77, 78, 81

Cmpd	R	R'	Method	Yield
**76**	Ph	NH_2	i	43%
**77**	Ph	NMe_2	ii	65%
**78**	OPh	NH_2	i	93%
**81**	Ph	OCH_2CCl_3	iii	96%

Table S2: *Reagents and conditions:* (i) **44**, THF, 67 °C, 30 h; (ii) **S8**, MeCN, 50 °C, 48 h

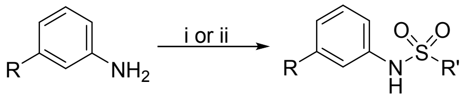

79, 80, 82, 83

Cmpd	R	R'	Method	Yield
**79**	Ph	OCH_2CCl_3	i	85%
**80**	OPh	OCH_2CCl_3	i	24%
**82**	Ph	OCH_2CF_3	ii	4%
**83**	H	OCH_2CCl_3	i	70%

3- Summary of Generic Analytical and Chromatographic Conditions

All commercial reagents were purchased from Sigma-Aldrich Chemical Company, Alfa Aesar, Apollo Scientific or Tokyo Chemical Industry UK Ltd. The chemicals were of the highest available purity. Unless otherwise stated, chemicals were used as supplied without further purification. Anhydrous solvents were obtained from AcroSeal™ or Aldrich SureSeal™ bottles and were stored under nitrogen. Petrol refers to the fraction with a boiling point between 40 and 60 °C.

Thin layer chromatography utilised to monitor reaction progress was conducted on plates pre-coated with silica gel Merck $60F_{254}$ or Merck NH_2F_{254S}. The eluent was as stated (where this consisted of more than one solvent, the ratio is stated as volume:volume) and visualisation was either by short wave (254 nm) ultraviolet light, or by treatment with the visualisation reagent stated followed by heating. 'Flash' medium pressure liquid chromatography (MPLC) was carried out either on a Biotage SP4 automated purification system or a Varian 971-FP automated purification system, using pre-packed Varian or Grace silica or amino-bonded silica cartridges.

All reactions carried out in a microwave were performed in a Biotage Initiator with Sixty robot.

Melting points were determined using a VWR Stuart SMP40 apparatus and are uncorrected.

^{1}H, ^{13}C and ^{19}F nuclear magnetic resonance (NMR) spectra were obtained as either $CDCl_3$, CD_3OD or DMSO-d_6 solutions and recorded at 500 MHz, 126 MHz and 471 MHz, respectively, on a Bruker Avance III 500 spectrometer. Where ^{13}C NMR data are not quoted, insufficient material was available or problems obtaining high resolution spectra were encountered. Chemical shifts are quoted in parts per million (δ) referenced to the appropriate deuterated solvent employed. Multiplicities are indicated by s (singlet), d (doublet), t (triplet), q (quartet), quin (quintet), m (multiplet), br (broad) or combinations thereof. Coupling constant values are given in Hz. Homonuclear and heteronuclear two dimensional NMR experiments were used where appropriate to facilitate assignment of chemical shifts.

LC-MS was carried out on a Waters Acquity UPLC system with PDA and ELSD employing positive or negative electrospray modes as appropriate to the individual

compound. Where LRMS data is not quoted, the mass was not recognised for that compound. High resolution mass spectrometry was performed by the EPSRC UK National Mass Spectrometry Facility, University of Wales Swansea, Singleton Park, Swansea, SA2 8PP.

FTIR spectra were recorded on either a Bio-Rad FTS 3000MX diamond ATR or an Agilent Cary 630 FTIR as a neat sample.

UV spectra were obtained using a U-2001 Hitachi Spectrophotometer with the sample dissolved in ethanol.

Data were compared with literature data for compounds which had been previously reported.

4- Experimental procedures

General synthetic procedures

General procedure A: To 3-bromophenyl bis(2,4-dimethoxybenzyl)sulfamate (**1**) in dimethoxyethane (7 mL/mmol of 3-bromophenyl sulfamate) was added a 2 M aq. solution of sodium bicarbonate (2 mol equiv.). The resulting solution was sparged with nitrogen for 15 min. The appropriate phenylboronic acid (1.2 mol equiv.) and [1,1′-bis(diphenylphosphino)ferrocene]dichloropalladium(II) complex with DCM (0.05 mol equiv.) were then added. The resulting mixture was heated at 80 °C for 20 min under microwave irradiation. Upon completion, the reaction mixture was diluted with water and EtOAc (10 mL, respectively), filtered through Celite and extracted with EtOAc (3 × 25 mL). The pooled organic extracts were washed with water and brine (30 mL, respectively), dried over $MgSO_4$ and concentrated *in vacuo*. The crude product was purified by column chromatography.

General procedure B: The appropriate 2,4-dimethoxybenzyl protected sulfamate (1 mol equiv.) was solubilised in a 10% TFA/DCM mixture (10 mL/mmol of sulfamate). The resulting solution was stirred at RT for 2 h. Upon completion, the solvent was removed *in vacuo*. The crude residue was dissolved in EtOAc (20 mL), washed with 10% aq. $NaHCO_3$ (20 mL) and extracted with EtOAc (3 × 25 mL). The combined organic extracts were washed with water (30 mL) and brine (30 mL), dried over $MgSO_4$ and concentrated *in vacuo*. The crude product was purified by column chromatography.

General procedure C: To 3-hydroxyphenyl bis(2,4-dimethoxybenzyl)sulfamate (**18**) (1 mol equiv.) and potassium carbonate (1.2 mol equiv.) in *N,N*-dimethylformamide was added the appropriate fluorobenzene (2 mol equiv.). The resulting mixture was heated at 180 °C for 30 min under microwave irradiation. After cooling, the mixture was concentrated *in vacuo*. The resulting residue was dissolved in saturated aq. $NaHCO_3$ (20 mL) and the mixture extracted with EtOAc (3 × 20 mL). The pooled organic extracts were washed with water (20 mL) and brine (20 mL), dried over $MgSO_4$ and concentrated *in vacuo*. The crude product was purified by column chromatography.

General procedure D: The appropriate nitrobenzene in MeOH (15 mL/mmol of nitrobenzene) and THF (5 mL/mmol of nitrobenzene) was subjected to palladium-catalysed hydrogenation using an H-Cube® reactor and a 10% Pd/C CatCart under a full pressure of hydrogen. The reaction was conducted at RT for 8 h. Upon completion, the solvents were removed *in vacuo* and the crude product was purified by column chromatography if required.

General procedure E: To the appropriate aniline (1 mol equiv.) in DCM (10 mL/mmol of aniline) were added triethylamine (2 mol equiv.) and acetic anhydride (1.2 mol equiv.). The resulting solution was stirred at RT overnight. Upon completion, the reaction mixture was diluted with DCM (10 mL), washed with saturated aq. $NaHCO_3$ (20 mL) and extracted with DCM (3 × 20 mL). The combined organic extracts were washed with water (30 mL) and brine (30 mL), dried over $MgSO_4$ and concentrated *in vacuo*. The crude product was purified by column chromatography.

General procedure F: To the appropriate aniline (1 mol equiv.) in THF (10 mL/mmol of aniline) was added 2,3-dimethyl-1-((2,2,2-trichloroethoxy)sulfonyl)-1*H*-imidazol-3-ium tetrafluoroborate (**44**) (3 mol equiv.). The resulting reaction mixture was heated at 67 °C for 30 h. Upon completion, the solvent was removed *in vacuo* to yield a crude product. The crude product was purified by column chromatography.

General procedure G: To the appropriate 2,2,2-trichloroethylsulfamoyloxyphenyl in MeOH (10 mL/mmol of phenyl) were added acetate buffer pH 4.65 (10 mL/mmol of protected aminophenyl) and zinc (10 mol equiv.). The resulting reaction mixture was heated at 60 °C for 2 h. Upon completion, the heterogeneous mixture was filtered through Celite and washed with MeOH (10 mL). The filtrate was concentrated *in vacuo* to give a crude oil, which was dissolved in water (10 mL) and washed with ether (10 mL). The crude product was converted to the sodium salt by ion-exchange chromatography (DOWEX 50WX2 - Na^+ form). The crude product was then subjected to column chromatography.

General procedure H: To the appropriate aniline (1 mol equiv.) in THF (10 mL/mmol of aniline), cooled in an ice bath, were added 4-(dimethylamino)pyridine (2.2 mol equiv.) and triethylamine (4 mol equiv.). The resulting solution was stirred at 0 °C for

10 min, and then a solution 2,2,2-trichloro ethyl chlorosulfate (**42**) (4 mol equiv.) in THF (5 mL/mmol of aniline) was added dropwise over 15 min. The resulting solution was stirred in an ice bath for 1 h and allowed to warm to RT. The reaction mixture was left stirring for an additional 24 h. Upon completion, the mixture was diluted with EtOAc (20 mL), quenched by the cautious addition of saturated aq. $NaHCO_3$ (20 mL) and extracted with EtOAc (3 × 20 mL). The pooled organic extracts were washed with water and brine (40 mL, respectively), dried over $MgSO_4$ and concentrated *in vacuo*. The crude product was subjected to column chromatography.

Synthetic procedures

3-Bromophenyl bis(2,4-dimethoxybenzyl)sulfamate, (1)

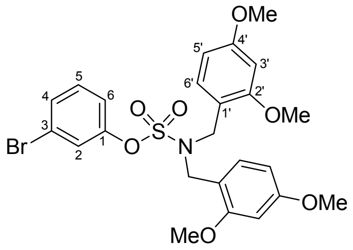

To 1-((3-bromophenoxy)sulfonyl)-2,3-dimethyl-1*H*-imidazol-3-ium tetrafluoroborate (**S5**) (3.0 g, 7.18 mmol) in acetonitrile (30 mL) was added a solution of bis(2,4-dimethoxybenzyl)amine (2.28 g, 7.18 mmol) in acetonitrile (25 mL). The reaction mixture was heated at 82 °C for 24 h. Upon completion, the solvent was removed *in vacuo* to yield a crude product. The crude product was purified by column chromatography (silica gel, petrol:EtOAc, 1:0 → 8:2) to yield the *title compound* as a clear oil (2.81 g, 71%); $R_f = 0.35$ (petrol:EtOAc, 8:2; $KMnO_4$); λ_{max} (EtOH)/nm 276.5; IR (neat) ν_{max}/cm^{-1} 1612, 1584, 1508, 1466, 1372, 1208, 1157; ^{1}H NMR (500 MHz, $CDCl_3$) δ 3.75 (6H, s, 2 × ArOC*H*$_3$), 3.81 (6H, s, 2 × ArOC*H*$_3$), 4.45 (4H, s, 2 × ArC*H*$_2$), 6.41 (2H, d, *J* = 2.4 Hz, H-3’), 6.45 (2H, dd, *J* = 8.4, 2.4 Hz, H-5’), 7.04 (1H, dd, *J* = 2.1, 2.1 Hz, H-2), 7.09 (1H, ddd, *J* = 8.4, 2.3 and 1.1 Hz, H-6), 7.16 (1H, dd, *J* = 8.1, 8.1 Hz, H-5), 7.26 (2H, d, *J* = 8.4 Hz, H-6’), 7.36 – 7.31 (1H, m, H-4); ^{13}C NMR (126 MHz, $CDCl_3$) δ 47.3 (Ar*C*H$_2$), 55.3 (ArO*C*H$_3$), 55.5 (ArO*C*H$_3$), 98.4 (C-3’), 104.2 (C-5’), 116.6 (C-1’), 120.8 (C-6), 122.4 (C-3), 125.5 (C-2), 129.6 (C-4), 130.6 (C-5), 131.2 (C-6’), 150.9 (C-1), 158.6 (C-2’ or C-4’), 160.8 (C-2’ or C 4’); HRMS (ESI) calcd for $C_{24}H_{27}BrNO_7S$ $[M(^{79}Br)+H]^+$ 552.0686, found 552.0675.

Methyl 3'-((*N*,*N*-bis(2,4-dimethoxybenzyl)sulfamoyl)oxy)-[1,1'-biphenyl]-2-carboxylate, (2)

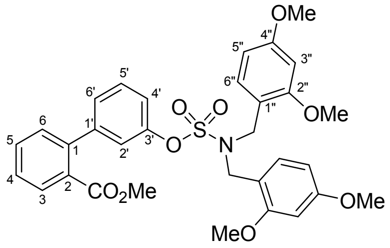

A solution of 3-bromophenyl bis(2,4-dimethoxybenzyl)sulfamate (**1**) (750 mg, 1.36 mmol) in acetonitrile (15 mL) was sparged with nitrogen for 15 min. To this solution, potassium carbonate (563 mg, 4.07 mmol), 2-methoxycarbonylphenyl boronic acid (293 mg, 1.63 mmol) and tetrakis(triphenylphosphine)palladium(0) (157 mg, 0.14 mmol) were added. The resulting mixture was heated at 120 °C for 20 min under microwave irradiation. Upon completion, the solvent was removed *in vacuo*. The crude residue was dissolved in a mixture of EtOAc and water (20 mL, respectively) and extracted with EtOAc (3 × 25 mL). The pooled organic extracts were washed with water (30 mL) and brine (30 mL), dried over $MgSO_4$ and concentrated *in vacuo*. The crude product was purified by column chromatography (silica gel, petrol:EtOAc, 1:0 → 7:3) to yield the *title compound* as a yellow oil (580 mg, 75%); R_f = 0.32 (petrol:EtOAc, 7:3); λ_{max} (EtOH)/nm 277.5; IR (neat) ν_{max}/cm^{-1} 1724, 1611, 1587, 1507, 1454, 1370, 1290, 1264, 1208, 1156; ^{1}H NMR (500 MHz, $CDCl_3$) δ 3.61 (3H, s, $ArCO_2CH_3$), 3.68 (6H, s, 2 × $ArOCH_3$), 3.77 (6H, s, 2 × $ArOCH_3$), 4.45 (4H, s, 2 × $ArCH_2$), 6.34 (2H, d, *J* = 2.4 Hz, H-3”), 6.43 (2H, dd, *J* = 8.4, 2.4 Hz, H-5”), 6.92 (1H, dd, *J* = 2.0, 2.0 Hz, H-2’), 7.18 – 7.12 (2H, m, H-4’ and H-6’), 7.31 – 7.27 (3H, m, H-3 and H-6”), 7.33 (1H, dd, *J* = 7.9, 7.9 Hz, H-5’), 7.44 (1H, ddd, *J* = 7.6, 7.6 and 1.3 Hz, H-4 or H-5), 7.53 (1H, ddd, *J* = 7.5, 7.5 and 1.4 Hz, H-4 or H-5), 7.85 (1H, dd, *J* = 7.7, 1.4 Hz, H-6); ^{13}C NMR (126 MHz, $CDCl_3$) δ 47.1 ($ArCH_2$), 52.2 ($ArCO_2CH_3$), 55.2 ($ArOCH_3$), 55.5 ($ArOCH_3$), 98.3 (C-3”), 104.1 (C-5”), 116.8 (C-1”), 120.9 (C-4’ or C-6’), 122.2 (C-2’), 126.5 (C-4’ or C-6’), 127.7 (C-4 or C-5), 129.1 (C-5’), 130.1 (C-6), 130.8 (C-3), 130.9 (C-2), 131.1 (C-6”), 131.4 (C-4 or C-5), 141.4 (C-1 or C-1’), 143.1 (C-1 or C-1’), 150.2 (C-3’), 158.6 (C-2” or C-4”), 160.7 (C-2” or C-4”), 168.8 ($ArCO_2CH_3$); HRMS (ESI) calcd for $C_{32}H_{37}N_2O_9S$ $[M+H]^+$ 625.2214, found 625.2211.

3'-((*N*,*N*-Bis(2,4-dimethoxybenzyl)sulfamoyl)oxy)-[1,1'-biphenyl]-3-carboxylic acid, (3)

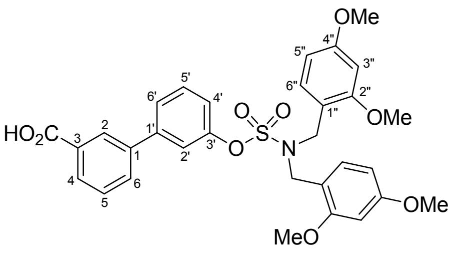

Compound **3** was synthesised according to general procedure A, using the following reagents: 3-bromophenyl bis(2,4-dimethoxybenzyl)sulfamate (**1**) (750 mg, 1.36 mmol), dimethoxyethane (9.5 mL), 2 M aq. solution of sodium bicarbonate (1.36 mL, 2.72 mmol), 3-carboxyphenylboronic acid pinacol ester (404 mg, 1.63 mmol) and [1,1′-bis(diphenylphosphino)ferrocene]dichloropalladium(II) complex with DCM (55 mg, 0.07 mmol). The crude product was purified by column chromatography (silica gel, petrol:EtOAc:AcOH, 1:0:0 → 50:49.5:0.5) to yield the *title compound* as a pale orange oil (637 mg, 79%); R_f = 0.32 (petrol:EtOAc:AcOH, 50:49.5:0.5); No λ_{max} (EtOH)/nm; IR (neat) ν_{max}/cm^{-1} 2976, 1701, 1611, 1588, 1507, 1371, 1291, 1208, 1154, 1036, 827; ^{1}H NMR (500 MHz, $CDCl_3$) δ 3.71 (6H, s, 2 × ArOC*H*$_3$), 3.76 (6H, s, 2 × ArOC*H*$_3$), 4.48 (4H, s, 2 × ArC*H*$_2$), 6.37 (2H, d, *J* = 2.4 Hz, H-3”), 6.44 (2H, dd, *J* = 8.4, 2.4 Hz, H-5”), 7.22 – 7.14 (2H, m, H-4’ and H-2’), 7.29 (2H, d, *J* = 8.3 Hz, H-6”), 7.40 (1H, d, *J* = 8.0 Hz, H-5’), 7.52 – 7.45 (1H, m, H-6’), 7.55 (1H, dd, *J* = 7.7, 7.7 Hz, H-5), 7.73 (1H, ddd, *J* = 7.8, 1.4 and 1.4 Hz, H-6), 8.11 (1H, ddd, *J* = 7.8, 1.4 and 1.4 Hz, H-4), 8.27 (1H, dd, *J* = 1.4 Hz, H-2); ^{13}C NMR (126 MHz, $CDCl_3$) δ 47.1 (Ar*C*H$_2$), 55.2 (ArO*C*H$_3$), 55.5 (ArO*C*H$_3$), 98.3 (C-3”), 104.1 (C-5”), 116.7 (C-1”), 120.8 (C-2’), 121.4 (C-4’), 125.2 (C-6’), 128.9 (C-2), 129.2 (C-5), 129.5 (C-4), 130.1 (C-5’), 131.2 (C-6”), 132.6 (C-6), 140.5 (C-1 or C-1’), 141.6 (C-1 or C-1’), 151.0 (C-3’), 158.6 (C-2” or C-4”), 160.7 (C-2” or C-4”), 177.2 (Ar*C*O$_2$H); LRMS (ES$^-$) *m/z* 592.3 [M-H]$^-$; HRMS (ESI) calcd for $C_{31}H_{30}NO_9S$ [M-H]$^-$ 592.1647, found 592.1644.

3'-((*N*,*N*-Bis(2,4-dimethoxybenzyl)sulfamoyl)oxy)-[1,1'-biphenyl]-4-carboxylic acid, (4)

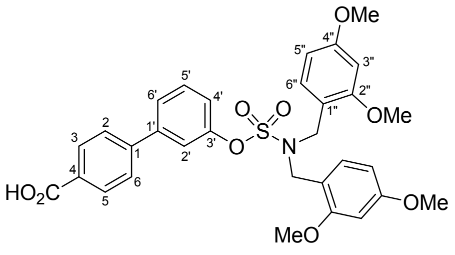

Compound **4** was synthesised according to general procedure A, using the following reagents: 3-bromophenyl bis(2,4-dimethoxybenzyl)sulfamate (**1**) (750 mg, 1.36 mmol), dimethoxyethane (9.5 mL), 2 M aq. solution of sodium bicarbonate (1.36 mL, 2.72 mmol), 4-carboxyphenylboronic acid pinacol ester (404 mg, 1.63 mmol) and [1,1′-bis(diphenylphosphino)ferrocene]dichloropalladium(II) complex with DCM (55 mg, 0.07 mmol). The crude product was purified by column chromatography (silica gel, petrol:EtOAc:AcOH, 1:0:0 → 50:49.5:0.5) to yield the *title compound* as a pale orange oil (620 mg, 77%); R_f = 0.28 (petrol:EtOAc:AcOH, 50:49.5:0.5); λ_{max} (EtOH)/nm 270.5; IR (neat) ν_{max}/cm^{-1} 2945, 1686, 1610, 1586, 1507, 1377, 1292, 1210, 1153, 1037, 890; ^{1}H NMR (500 MHz, $CDCl_3$) δ 3.71 (6H, s, 2 × ArOC*H*$_3$), 3.78 (6H, s, 2 × ArOC*H*$_3$), 4.48 (4H, s, 2 × ArC*H*$_2$), 6.39 (2H, d, *J* = 2.4 Hz, H-3”), 6.45 (2H, dd, *J* = 8.4, 2.4 Hz, H-5”), 7.15 (1H, dd, *J* = 2.0, 2.0 Hz, H-2’), 7.20 (1H, ddd, *J* = 8.2, 2.3 and 1.0 Hz, H-4’), 7.30 (2H, d, *J* = 8.4 Hz, H-6”), 7.41 (1H, dd, *J* = 7.9, 7.9 Hz, H-5’), 7.50 – 7.46 (1H, m, H-6’), 7.59 (1H, d, *J* = 8.5 Hz, H-2, 6), 8.17 (2H, d, *J* = 8.5 Hz, H-3, 5); ^{13}C NMR (126 MHz, $CDCl_3$) δ 47.1 (Ar*C*H$_2$), 55.2 (ArO*C*H$_3$), 55.5 (ArO*C*H$_3$), 98.4 (C-3”), 104.1 (C-5”), 116.7 (C-1”), 120.9 (C-2’), 121.9 (C-4’), 125.3 (C-6’), 127.3 (C-2, 6), 128.6 (C-4), 130.1 (C-5’), 130.8 (C-3, 5), 131.2 (C-6”), 141.5 (C-1 or C-1’), 145.2 (C-1 or C-1’), 151.0 (C-3), 158.6 (C-2” or C-4”), 160.8 (C-2” or C-4”), 177.4 (Ar*C*O$_2$H); LRMS (ES$^-$) *m/z* 592.4 [M-H]$^-$; HRMS (ESI) calcd for $C_{31}H_{30}NO_9S$ [M-H]$^-$ 592.1647, found 592.1644.

2'-Amino-[1,1'-biphenyl]-3-yl bis(2,4-dimethoxybenzyl)sulfamate, (5)

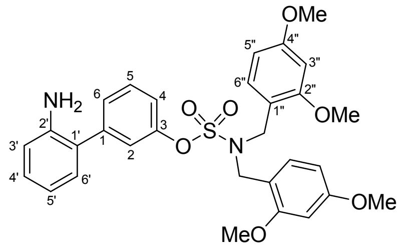

Compound **5** was synthesised according to general procedure A, using the following reagents: 3-bromophenyl bis(2,4-dimethoxybenzyl)sulfamate (**1**) (900 mg, 1.63 mmol), dimethoxyethane (11.4 mL), 2 M aq. solution of sodium bicarbonate (1.63 mL, 3.26 mmol), 2-aminophenylboronic acid pinacol ester (428 mg, 1.95 mmol) and [1,1′-bis(diphenylphosphino)ferrocene]dichloropalladium(II) complex with DCM (67 mg, 0.08 mmol). The crude product was purified by column chromatography (silica gel, petrol:EtOAc, 1:0 → 7:3) to yield the *title compound* as a brown oil (755 mg, 82%); R_f = 0.31 (petrol:EtOAc, 7:3; ninhydrin); λ_{max} (EtOH)/nm 225.5; IR (neat) ν_{max}/cm^{-1} 3442, 3360, 1610, 1582, 1503, 1458, 1350, 1289, 1208, 1154, 887; ^{1}H NMR (500 MHz, $CDCl_3$) δ 3.69 (6H, s, 2 × ArOC*H*$_3$), 3.73 (2H, s, ArN*H*$_2$), 3.77 (6H, s, 2 × ArOC*H*$_3$), 4.46 (4H, s, 2 × ArC*H*$_2$), 6.36 (2H, d, *J* = 2.4 Hz, H-3”), 6.43 (2H, dd, *J* = 8.4, 2.4 Hz, H-5”), 6.75 (1H, dd, *J* = 7.9, 1.1 Hz, H-3’), 6.81 (1H, ddd, *J* = 7.5, 7.5 and 1.2 Hz, H-5’), 7.08 – 7.04 (2H, m, H-2 and H-6’), 7.18 – 7.11 (2H, m, H-4 and H-4’), 7.28 (2H, d, *J* = 8.4 Hz, H-6”), 7.34 – 7.31 (1H, m, H-6), 7.38 (1H, dd, *J* = 7.7, 7.7 Hz, H-5); ^{13}C NMR (126 MHz, $CDCl_3$) δ 47.1 (Ar*C*H$_2$), 55.2 (ArO*C*H$_3$), 55.5 (ArO*C*H$_3$), 98.3 (C-3”), 104.1 (C-5”), 115.8 (C-3’), 116.7 (C-1”), 118.7 (C-5’), 120.8 (C-4), 122.8 (C-2), 126.3 (C-2’), 127.2 (C-6), 129.0 (C-4’), 130.0 (C-5), 130.5 (C-6’), 131.1 (C-6”), 141.2 (C-1 or C-1’), 143.6 (C-1 or C-1’), 150.8 (C-3), 158.6 (C-2” or C-4”), 160.7 (C-2” or C-4”); LRMS (ES^{+}) *m/z* 565.4 [M+H]$^{+}$; HRMS (ESI) calcd for $C_{30}H_{33}N_2O_7S$ [M+H]$^{+}$ 565.2003, found 565.1992.

3'-Amino-[1,1'-biphenyl]-3-yl bis(2,4-dimethoxybenzyl)sulfamate, (6)

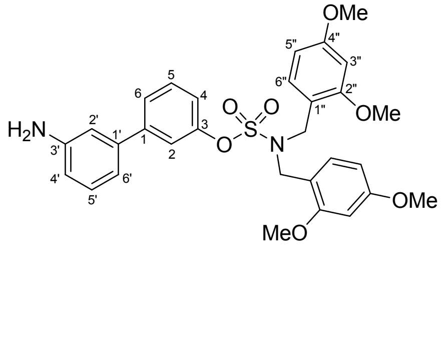

Compound **6** was synthesised according to general procedure A, using the following reagents: 3-bromophenyl bis(2,4-dimethoxybenzyl)sulfamate (**1**) (900 mg, 1.63 mmol), dimethoxyethane (11.4 mL), 2 M aq. solution of sodium bicarbonate (1.63 mL, 3.26 mmol), 3-aminophenylboronic acid pinacol ester (428 mg, 1.95 mmol) and [1,1′-bis(diphenylphosphino)ferrocene]dichloropalladium(II) complex with DCM (67 mg, 0.08 mmol). The crude product was purified by column chromatography (silica gel, petrol:EtOAc, 1:0 → 6:4) to yield the *title compound* as a brown oil (709 mg, 77%); R_f = 0.30 (petrol:EtOAc, 6:4; ninhydrin); λ_{max} (EtOH)/nm 221.5; IR (neat) ν_{max}/cm^{-1} 3465, 3379, 1610, 1588, 1507, 1464, 1367, 1291, 1207, 1156, 1132, 1034, 845; ^{1}H NMR (500 MHz, $CDCl_3$) δ 3.68 (6H, s, 2 × ArOC*H*$_3$), 3.79 (6H, s, 2 × ArOC*H*$_3$), 4.45 (4H, s, 2 × ArC*H*$_2$), 6.39 (2H, d, *J* = 2.4 Hz, H-3”), 6.45 (2H, dd, *J* = 8.4, 2.4 Hz, H-5”), 6.69 (1H, ddd, *J* = 7.9, 2.3 and 1.0 Hz, H-4’), 6.77 (1H, dd, *J* = 2.0, 2.0 Hz, H-2’), 6.90 (1H, ddd, *J* = 7.8, 1.2 and 1.2 Hz, H-6’), 7.03 (1H, dd, *J* = 2.1, 2.1 Hz, H-2), 7.13 (1H, ddd, *J* = 8.0, 2.3 and 1.1 Hz, H-4), 7.21 (1H, dd, *J* = 7.8, 7.8 Hz, H-5’), 7.30 (2H, d, *J* = 8.3 Hz, H-6”), 7.33 (1H, dd, *J* = 7.9, 7.9 Hz, H-5), 7.44 – 7.40 (1H, m, H-6); ^{13}C NMR (126 MHz, $CDCl_3$) δ 47.1 (Ar*C*H$_2$), 55.2 (ArO*C*H$_3$), 55.5 (ArO*C*H$_3$), 98.4 (C-3”), 104.1 (C-5”), 113.9 (C-2’), 114.6 (C-4’), 116.9 (C-1”), 117.6 (C-6’), 120.6 (C-2 or C-4), 120.8 (C-2 or C-4), 125.1 (C-6), 129.7 (C-5 or C-5’), 129.8 (C-5 or C-5’), 131.2 (C-6”), 141.1 (C-1 or C-1’), 143.0 (C-1 or C-1’), 147.0 (C-3’), 150.8 (C-3), 158.6 (C-2” or C-4”), 160.7 (C-2” or C-4”); LRMS (ES$^+$) *m/z* 565.5 [M+H]$^+$; HRMS (ESI) calcd for $C_{30}H_{33}N_2O_7S$ [M+H]$^+$ 565.2003, found 565.2000.

4'-Amino-[1,1'-biphenyl]-3-yl bis(2,4-dimethoxybenzyl)sulfamate, (7)

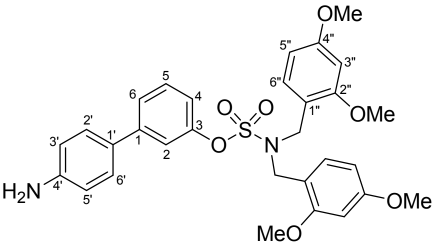

Compound **7** was synthesised according to general procedure A, using the following reagents: 3-bromophenyl bis(2,4-dimethoxybenzyl)sulfamate (**1**) (500 mg, 0.90 mmol), dimethoxyethane (6.3 mL), 2 M aq. solution of sodium bicarbonate (0.91 mL, 1.81 mmol), 4-aminobenzeneboronic acid pinacol ester (238 mg, 1.09 mmol) and [1,1′-bis(diphenylphosphino)ferrocene]dichloropalladium(II) complex with DCM (37 mg,

0.04 mmol). The crude product was purified by column chromatography (silica gel, petrol:EtOAc, 1:0 → 1:1) to yield the *title compound* as a brown oil (368 mg, 72%); R_f = 0.33 (petrol:EtOAc, 1:1; ninhydrin); λ_{max} (EtOH)/nm 283.0; IR (neat) ν_{max}/cm^{-1} 3468, 3380, 1610, 1587, 1507, 1364, 1290, 1207, 1156, 1130, 1034, 884; ^{1}H NMR (500 MHz, $CDCl_3$) δ 3.70 (6H, s, 2 × ArOCH_3), 3.75 (2H, s, ArNH_2), 3.79 (6H, s, 2 × ArOCH_3), 4.48 (4H, s, 2 × ArCH_2), 6.38 (2H, d, *J* = 2.3 Hz, H-3"), 6.44 (2H, dd, *J* = 8.4, 2.4 Hz, H-5"), 6.74 (2H, d, *J* = 8.5 Hz, H-3', 5'), 7.05 (1H, ddd, *J* = 8.3, 2.5 and 1.0 Hz, H-4), 7.14 (1H, dd, *J* = 2.1, 2.1 Hz, H-2), 7.29 (2H, d, *J* = 8.4 Hz, H-6"), 7.31 (1H, dd, *J* = 7.7, 7.7 Hz, H-5), 7.33 (2H, d, *J* = 8.5 Hz, H-2', 6'), 7.41 – 7.36 (1H, m, H-6); ^{13}C NMR (126 MHz, $CDCl_3$) δ 47.1 (ArCH$_2$), 55.2 (ArOCH$_3$), 55.5 (ArOCH$_3$), 98.3 (C-3"), 104.1 (C-5"), 115.4 (C-3', 5'), 116.8 (C-1"), 119.7 (C-2 or C-4), 119.8 (C-2 or C-4), 124.3 (C-6), 128.2 (C-2', 6'), 129.7 (C-5), 130.2 (C-4'), 131.2 (C-6"), 142.9 (C-1 or C-1'), 146.4 (C-1 or C-1'), 150.9 (C-3), 158.6 (C-2" or C-4"), 160.7 (C-2" or C-4"); LRMS (ES^+) *m/z* 565.4 $[M+H]^+$; HRMS (ESI) calcd for $C_{30}H_{33}N_2O_7S$ $[M+H]^+$ 565.2003, found 565.1998.

Methyl 3'-(sulfamoyloxy)-[1,1'-biphenyl]-2-carboxylate, (8)

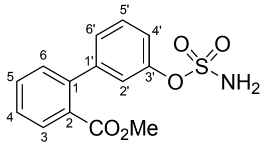

Compound **8** was synthesised according to general procedure B, using the following reagents: methyl 3'-((*N*,*N*-bis(2,4-dimethoxybenzyl)sulfamoyl)oxy)-[1,1'-biphenyl]-2-carboxylate (**2**) (250 mg, 0.41 mmol), DCM (3.7 mL) and TFA (0.4 mL). The crude product was purified by column chromatography (silica gel, petrol:EtOAc, 1:0 → 6:4) to yield the *title compound* as a pale yellow oil (112 mg, 88%); R_f = 0.34 (petrol:EtOAc, 6:4); λ_{max} (EtOH)/nm 280.0; IR (neat) ν_{max}/cm^{-1} 3364, 3263, 1708, 1568, 1375, 1292, 1264, 1190, 1147, 1093; ^{1}H NMR (500 MHz, DMSO-d_6) δ 3.61 (s, 3H, ArCO$_2$CH_3), 7.19 (dd, *J* = 2.0, 2.0 Hz, 1H, H-2'), 7.31 – 7.24 (m, 2H, H-4' and H-6'), 7.47 (dd, *J* = 7.7, 1.2 Hz, 1H, H-3), 7.51 – 7.49 (m, 1H, H-5'), 7.55 – 7.51 (m, 1H, H-4 or H-5), 7.66 (ddd, *J* = 7.6, 7.6, 1.4 Hz, 1H, H-4 or H-5), 7.77 (dd, *J* = 7.7, 1.4 Hz, 1H, H-6), 8.05 (s, 2H, ArOSO$_2$NH_2); ^{13}C NMR (126 MHz, DMSO-d_6) δ 52.1 (ArCO$_2$$CH_3$), 121.0 (C-4' or C-6'), 121.8 (C-2'), 126.3 (C-4' or C-6'), 127.9 (C-4 or C-5), 129.4 (C-6), 129.6 (C-5'), 130.6 (C-3), 130.7 (C-2), 131.6 (C-4 or C-5), 139.9 (C-1 or C-1'), 142.0 (C-1 or C-

1’), 149.9 (C-3’), 168.2 (Ar*C*O_2CH_3); LRMS (ES^-) *m/z* 306.1 $[M-H]^-$; HRMS (ESI) calcd for $C_{14}H_{12}NO_5S$ $[M-H]^-$ 306.0442, found 306.0442.

3'-(Sulfamoyloxy)-[1,1'-biphenyl]-3-carboxylic acid, (9)

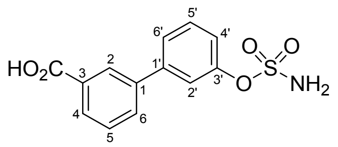

Compound **9** was synthesised according to general procedure B, using the following reagents: 3'-((*N*,*N*-bis(2,4-dimethoxybenzyl)sulfamoyl)oxy)-[1,1'-biphenyl]-3-carboxylic acid (**3**) (250 mg, 0.42 mmol), DCM (3.8 mL) and TFA (0.5 mL). The crude product was purified by column chromatography (silica gel, petrol:EtOAc:AcOH, 1:0:0 → 50:49.5:0.5) to yield the *title compound* as a pale pink solid (102 mg, 82%); R_f = 0.29 (petrol:EtOAc:AcOH, 50:49.5:0.5); m.p. 168.0-170.0 °C; λ_{max} (EtOH)/nm 250.0; IR (neat) ν_{max}/cm^{-1} 3368, 3274, 1677, 1606, 1577, 1459, 1362, 1312, 1148, 932; ^1H NMR (500 MHz, DMSO-d_6) δ 7.37 – 7.28 (1H, m, H-4’), 7.58 (1H, dd, *J* = 7.8, 7.8 Hz, H-5 or H-5’), 7.60 (1H, dd, *J* = 2.0, 2.0 Hz, H-2’), 7.63 (1H, dd, *J* = 7.7, 7.7 Hz, H-5 or H-5’), 7.71 – 7.66 (1H, m, H-6’), 7.95 (1H, ddd, *J* = 7.8, 1.4 and 1.4 Hz, H-4 or H-6), 7.98 (1H, ddd, *J* = 7.6, 1.3 and 1.3 Hz, H-4 or H-6), 8.06 (2H, s, $ArOSO_2NH_2$), 8.21 (1H, dd, *J* = 1.8, 1.8 Hz, H-2), 13.13 (1H, s, $ArCO_2H$); ^{13}C NMR (126 MHz, DMSO-d_6) δ 120.6 (C-2’), 121.8 (C-4’), 125.0 (C-6’), 127.4 (C-2), 128.8 (C-4 or C-6), 129.5 (C-5 or C-5’), 130.5 (C-5 or C-5’), 131.3 (C-4 or C-6), 131.6 (C-3), 139.4 (C-1 or C-1’), 141.0 (C-1 or C-1’), 150.8 (C-3’), 167.1 (Ar*C*O_2H); LRMS (ES^-) *m/z* 292.1 $[M-H]^-$; HRMS (ESI) calcd for $C_{13}H_{10}NO_5S$ $[M-H]^-$ 292.0285, found 292.0286.

3'-(Sulfamoyloxy)-[1,1'-biphenyl]-4-carboxylic acid, (10)

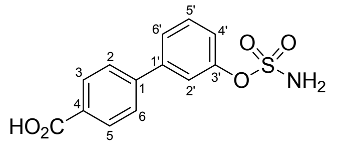

Compound **10** was synthesised according to general procedure B, using the following reagents: 3'-((*N*,*N*-bis(2,4-dimethoxybenzyl)sulfamoyl)oxy)-[1,1'-biphenyl]-4-carboxylic acid (**4**) (250 mg, 0.42 mmol), DCM (3.8 mL) and TFA (0.5 mL). The crude product was purified by column chromatography (silica gel, petrol:EtOAc:AcOH, 1:0:0 → 50:49.5:0.5) to yield the *title compound* as an off-white solid (112 mg, 90%); R_f =

0.26 (petrol:EtOAc:AcOH, 50:49.5:0.5); m.p. 190.0-192.0 °C; λ_{max} (EtOH)/nm 266.0; IR (neat) ν_{max}/cm^{-1} 3393, 3300, 1680, 1608, 1378, 1368, 1301, 1201, 1150, 912; ^{1}H NMR (500 MHz, DMSO-d_6) δ 7.34 (1H, ddd, *J* = 8.2, 2.3 and 0.9 Hz, H-4'), 7.59 (1H, dd, *J* = 7.9, 7.9 Hz, H-5'), 7.62 (1H, dd, *J* = 2.0, 2.0 Hz, H-2'), 7.71 (ddd, *J* = 7.7, 1.3 and 1.3 Hz, 1H, H-6'), 7.82 (2H, d, *J* = 8.3 Hz, H-2, 6), 8.05 (2H, d, *J* = 8.3 Hz, H-3, 5), 8.06 (2H, s, $ArOSO_2NH_2$), 12.98 (brs, 1H, $ArCO_2H$); ^{13}C NMR (126 MHz, DMSO-d_6) δ 120.7 (C-2'), 122.1 (C-4'), 125.2 (C-6'), 127.0 (C-2, 6), 130.0 (C-3, 5), 130.1 (C-4), 130.5 (C-5'), 140.8 (C-1 or C-1'), 143.1 (C-1 or C-1'), 150.7 (C-3'), 167.0 ($ArCO_2H$); LRMS (ES$^-$) *m/z* 292.1 [M-H]$^-$; HRMS (ESI) calcd for $C_{13}H_{10}NO_5S$ [M-H]$^-$ 292.0285, found 292.0277.

2'-Amino-[1,1'-biphenyl]-3-yl sulfamate, (11)

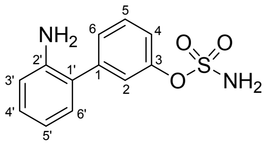

Compound **11** was synthesised according to general procedure B, using the following reagents: 2'-amino-[1,1'-biphenyl]-3-yl bis(2,4-dimethoxybenzyl)sulfamate (**5**) (330 mg, 0.58 mmol), DCM (5.3 mL) and TFA (0.6 mL). The crude product was purified by column chromatography (silica gel, petrol:EtOAc, 1:0 → 6:4) to yield the *title compound* as a brown solid (128 mg, 83%); R_f = 0.29 (petrol:EtOAc, 6:4; ninhydrin); m.p. 101.5-103.5 °C; λ_{max} (EtOH)/nm 225.0; IR (neat) ν_{max}/cm^{-1} 3358, 1611, 1474, 1357, 1186, 1147, 895; ^{1}H NMR (500 MHz, DMSO-d_6) δ 4.89 (2H, s, $ArNH_2$), 6.64 (1H, ddd, *J* = 7.4, 7.4 and 1.2 Hz, H-5'), 6.76 (1H, dd, *J* = 8.0, 1.2 Hz, H-3'), 7.01 (1H, dd, *J* = 7.6, 1.6 Hz, H-6'), 7.07 (1H, ddd, *J* = 8.4, 7.3 and 1.6 Hz, H-4'), 7.23 (1H, ddd, *J* = 8.2, 2.3 and 1.3 Hz, H-4), 7.40 – 7.33 (2H, m, H-2 and H-6), 7.53 (1H, dd, *J* = 8.2, 8.2 Hz, H-5), 7.98 (2H, s, $ArOSO_2NH_2$); ^{13}C NMR (126 MHz, DMSO-d_6) δ 115.3 (C-3'), 116.7 (C-5'), 120.8 (C-4), 122.4 (C-2), 124.3 (C-2'), 127.0 (C-6), 128.6 (C-4'), 130.0 (C-5 or C-6'), 130.1 (C-5 or C-6'), 141.4 (C-1 or C-1'), 145.1 (C-1 or C-1'), 150.2 (C-3); LRMS (ES$^-$) *m/z* 263.2 [M-H]$^-$; HRMS (ESI) calcd for $C_{12}H_{11}N_2O_3S$ [M-H]$^-$ 263.0496, found 263.0497.

3'-Amino-[1,1'-biphenyl]-3-yl sulfamate, (12)

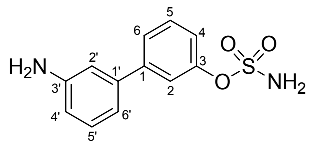

Compound **12** was synthesised according to general procedure B, using the following reagents: 3'-amino-[1,1'-biphenyl]-3-yl bis(2,4-dimethoxybenzyl)sulfamate (**6**) (150 mg, 0.27 mmol), DCM (2.4 mL) and TFA (0.3 mL). The crude product was purified by column chromatography (silica gel, petrol:EtOAc, 1:0 → 1:1) to yield the *title compound* as a brown solid (42 mg, 82%); R_f = 0.33 (petrol:EtOAc, 1:1; ninhydrin); m.p. 106.5-108.5 °C; λ_{max} (EtOH)/nm 235.5; IR (neat) ν_{max}/cm^{-1} 3413, 3340, 3292, 3040, 1605, 1576, 1473, 1362, 1230, 1146, 1086, 947; ^{1}H NMR (500 MHz, DMSO-d_6) δ 5.21 (2H, s, ArNH_2), 6.59 (1H, ddd, J = 8.0, 2.3 and 1.0 Hz, H-4’), 6.81 – 6.76 (1H, m, H-6’), 6.84 (1H, dd, J = 2.0, 2.0 Hz, H-2’), 7.12 (1H, dd, J = 7.8, 7.8 Hz, H-5’), 7.28 – 7.19 (1H, m, H-4), 7.47 – 7.39 (1H, m, H-2), 7.54 – 7.47 (2H, m, H-5 and H-6), 8.02 (2H, s, ArOSO$_2$NH_2); ^{13}C NMR (126 MHz, DMSO-d_6) δ 112.0 (C-2’), 113.6 (C-4’), 114.3 (C-6), 120.1 (C-2), 120.8 (C-4), 124.6 (C-6), 129.5 (C-5’), 130.0 (C-5), 139.7 (C-1 or C-1’), 142.9 (C-1 or C-1’), 149.2 (C-3 or C-3’), 150.5 (C-3 or C-3’); LRMS (ES$^-$) m/z 263.2 [M-H]$^-$; HRMS (ESI) calcd for $C_{12}H_{11}N_2O_3S$ [M-H]$^-$ 263.0496, found 263.0498.

4'-Amino-[1,1'-biphenyl]-3-yl sulfamate, (13)

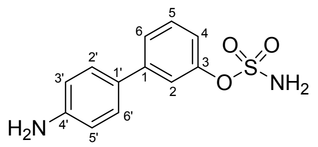

Compound **13** was synthesised according to general procedure C, using the following reagents: 4'-amino-[1,1'-biphenyl]-3-yl bis(2,4-dimethoxybenzyl)sulfamate (**7**) (110 mg, 0.19 mmol), DCM (1.7 mL) and TFA (0.2 mL). The crude product was purified by column chromatography (silica gel, petrol:EtOAc, 1:0 → 4:6) to yield the *title compound* as a pale brown solid (42 mg, 82%); R_f = 0.29 (petrol:EtOAc, 4:6; ninhydrin); m.p. 144.5-146.5 °C; λ_{max} (EtOH)/nm 287.5; IR (neat) ν_{max}/cm^{-1} 3417, 3342, 2901, 1604, 1573, 1525, 1480, 1363, 1149, 910; ^{1}H NMR (500 MHz, DMSO-d_6) δ 5.32 (2H, s, ArNH_2), 6.65 (2H, d, J = 8.4 Hz, H-3’, 5’), 7.11 (1H, ddd, J = 8.4, 2.3 and 1.0 Hz, H-4), 7.37 (2H, d, J = 8.5 Hz, H-2’, 6’), 7.41 (1H, dd, J = 2.0, 2.0 Hz, H-2), 7.43 (1H, dd, J = 7.9, 7.9 Hz, H-5), 7.51 – 7.46 (1H, m, H-6), 7.98 (2H, s,

$ArOSO_2NH_2$); ^{13}C NMR (126 MHz, DMSO-d_6) δ 114.2 (C-3', 5'), 118.9 (C-2), 119.2 (C-4), 123.4 (C-6), 126.1 (C_q Ar), 127.3 (C-2', 6'), 129.9 (C-5), 142.6 (C_q Ar), 148.9 (C_q Ar), 150.7 (C_q Ar); LRMS (ES^-) *m/z* 263.2 $[M-H]^-$; HRMS (ESI) calcd for $C_{12}H_{11}N_2O_3S$ $[M-H]^-$ 263.0496, found 263.0497.

3'-((*N*,*N*-Bis(2,4-dimethoxybenzyl)sulfamoyl)oxy)-[1,1'-biphenyl]-2-carboxylic acid, (14)

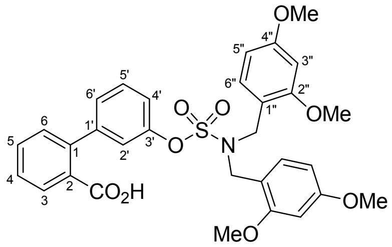

To methyl 3'-((*N*,*N*-bis(2,4-dimethoxybenzyl)sulfamoyl)oxy)-[1,1'-biphenyl]-2-carboxylate (**2**) (300 mg, 0.49 mmol) in THF (5 mL) was added a 2M aq. solution of lithium hydroxide (2.47 mL, 4.94 mmol). The resulting mixture was heated at 60 °C for 18 h. Upon completion, the mixture was acidified to pH 3 using a 4 M aq. solution of HCl. The reaction was then diluted with water (20 mL) and extracted with EtOAc (3 × 25 mL). The pooled organic extracts were washed with water (30 mL) and brine (30 mL), dried over $MgSO_4$ and concentrated *in vacuo*. The crude product was purified by column chromatography (silica gel, petrol:EtOAc:AcOH, 1:0:0 → 50:49.5:0.5) to yield the *title compound* as a yellow oil (235 mg, 80%); R_f = 0.28 (petrol:EtOAc:AcOH, 50:49.5:0.5); λ_{max} (EtOH)/nm 275.5; IR (neat) ν_{max}/cm^{-1} 2945, 1693, 1610, 1586, 1506, 1458, 1353, 1292, 1209, 1177, 1154, 1037, 890; ^1H NMR (500 MHz, $CDCl_3$) δ 3.68 (6H, s, 2 × ArOCH_3), 3.73 (6H, s, 2 × ArOCH_3), 4.41 (4H, s, 2 × ArCH_2), 6.33 (2H, d, *J* = 2.4 Hz, H-3"), 6.41 (2H, dd, *J* = 8.3, 2.4 Hz, H-5"), 6.79 (1H, dd, *J* = 2.0, 2.0 Hz, H-2'), 7.13 (1H, ddd, *J* = 8.1, 2.3 and 1.0 Hz, H-4' or H-6'), 7.19 – 7.15 (1H, m, H-4' or H-6'), 7.27 (3H, d, *J* = 8.5 Hz, H-3 and H-6"), 7.32 (1H, dd, *J* = 7.9, 7.9 Hz, H-5'), 7.44 (1H, ddd, *J* = 7.6, 7.6 and 1.3 Hz, H-4 or H-5), 7.56 (1H, ddd, *J* = 7.5, 7.5 and 1.5 Hz, H-4 or H-5), 7.94 (1H, dd, *J* = 7.8, 1.5 Hz, H-6); ^{13}C NMR (126 MHz, $CDCl_3$) δ 47.2 (ArCH_2), 55.2 (ArOCH_3), 55.5 (ArOCH_3), 98.4 (C-3"), 104.3 (C-5"), 116.8 (C-1"), 121.2 (C-4' or C-6'), 122.3 (C-2'), 126.7 (C-4' or C-6'), 127.7 (C-4 or C-5), 129.2 (C-5'), 129.3 (C-2), 130.9 (C-3), 131.1 (C-6"), 131.2 (C-6), 132.1 (C-4 or C-5), 142.3 (C-1 or C-1'), 143.0 (C-1 or C-1'), 150.1 (C-3'), 158.4 (C-2" or C-4"), 160.7 (C-2" or

C-4”), 171.2 (Ar*C*O$_2$H); LRMS (ES$^-$) *m/z* 592.3 [M-H]$^-$; HRMS (ESI) calcd for $C_{31}H_{30}NO_9S$ [M-H]$^-$ 592.1647, found 592.1643.

3'-(Sulfamoyloxy)-[1,1'-biphenyl]-2-carboxylic acid, (15)

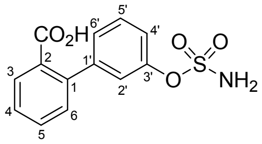

Compound **15** was synthesised according to general procedure B, using the following reagents: 3'-((*N*,*N*-bis(2,4-dimethoxybenzyl)sulfamoyl)oxy)-[1,1'-biphenyl]-2-carboxylic acid (**14**) (200 mg, 0.34 mmol), DCM (3.1 mL) and TFA (0.4 mL). The crude product was purified by column chromatography (silica gel, petrol:EtOAc:AcOH, 1:0:0 → 50:49.5:0.5) to yield the *title compound* as a pale pink solid (88 mg, 89%); R_f = 0.26 (petrol:EtOAc:AcOH, 50:49.5:0.5); m.p. 162.0-164.0 °C; λ_{max} (EtOH)/nm 280.0; IR (neat) ν_{max}/cm^{-1} 3370, 3269, 1697, 1477, 1346, 1311, 1200, 1180, 1143, 906; ^{1}H NMR (500 MHz, DMSO-*d*$_6$) δ 7.25 (1H, dd, *J* = 2.0, 2.0 Hz, H-2’), 7.34 – 7.26 (2H, m, H-4’ and H-6’), 7.43 (1H, dd, *J* = 7.8, 1.2 Hz, H-3), 7.54 – 7.46 (2H, m, 2 × Ar*H*), 7.61 (1H, ddd, *J* = 7.6, 7.6 and 1.4 Hz, H-4), 7.77 (1H, dd, *J* = 7.8, 1.4 Hz, H-6), 8.04 (2H, s, ArOSO$_2$N*H*$_2$), 12.83 (1H, brs, ArCO$_2$*H*); ^{13}C NMR (126 MHz, DMSO-*d*$_6$) δ 120.9 (C-4’ or C-6’), 122.0 (C-2’), 126.6 (C-4’ or C-6’), 127.8 (CH Ar), 129.3 (CH Ar), 129.4 (CH Ar), 130.6 (CH Ar), 131.1 (C-3), 132.1 (C-2), 139.7 (C-1 or C-1’), 142.4 (C-1 or C-1’), 149.9 (C-3’), 169.3 (Ar*C*O$_2$H); LRMS (ES$^-$) *m/z* 292.2 [M-H]$^-$; HRMS (ESI) calcd for $C_{13}H_{10}NO_5S$ [M-H]$^-$ 292.0285, found 292.0286.

3-Hydroxyphenyl 2-methyl-1*H*-imidazole-1-sulfonate, (17)

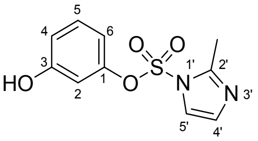

To 1,1'-sulfonylbis(2-methyl-1*H*-imidazole) (**S2**) (2.0 g, 8.84 mmol) and caesium carbonate (2.88 g, 8.84 mmol) in acetonitrile was added resorcinol (**16**) (4.87 g, 44.2 mmol). The resulting mixture was heated at 120 °C for 15 min under microwave irradiation. After cooling, the mixture was concentrated *in vacuo*. The resulting residue was dissolved in a saturated aq. NH_4Cl (20 mL) and the mixture extracted with EtOAc (3 × 20 mL). The pooled organic extracts were washed with water (2 × 20 mL) and

brine (20 mL), dried over $MgSO_4$ and concentrated *in vacuo*. The crude yellow solid was purified by column chromatography (silica gel, petrol:EtOAc, 1:0 → 6:4) to yield the *title compound* as a pale yellow solid (1.80 g, 80%); R_f = 0.30 (petrol:EtOAc, 6:4; $KMnO_4$); m.p. 122.0-124.0 °C; λ_{max} (EtOH)/nm 274.0; IR (neat) ν_{max}/cm^{-1} 1618, 1585, 1556, 1463, 1427, 1381, 1204, 1181, 1154, 1114, 949; ^{1}H NMR (500 MHz, $CDCl_3$) δ 2.48 (3H, s, CH_3), 6.16 (1H, dd, *J* = 2.3, 2.3 Hz, H-2), 6.67 (1H, ddd, *J* = 8.2, 2.4 and 0.9 Hz, H-4 or H-6), 6.89 – 6.82 (2H, m, H-4 or H-6 and H-4' or H-5'), 7.21 (1H, d, *J* = 1.8 Hz, H-4' or H-5'), 7.26 (1H, d, *J* = 8.3 Hz, H-5), 9.43 (1H, s, ArO*H*); ^{13}C NMR (126 MHz, $CDCl_3$) δ 14.6 (CH_3), 107.8 (C-2), 113.2 (C-4 or C-6), 116.6 (C-4 or C-6), 120.9 (C-4' or C-5'), 126.7 (C-4' or C-5'), 131.2 (C-5), 147.4 (C-1 or C-2'), 149.6 (C-1 or C-2'), 158.6 (C-3); LRMS (ES$^+$) *m/z* 255.2 [M+H]$^+$; HRMS (ESI) calcd for $C_{10}H_{11}N_2O_4S$ [M+H]$^+$ 255.0434, found 255.0439.

3-Hydroxyphenyl bis(2,4-dimethoxybenzyl)sulfamate, (18)

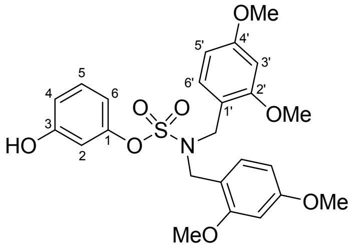

To 3-hydroxyphenyl 2-methyl-1*H*-imidazole-1-sulfonate (**17**) (1.8 g, 7.08 mmol) in a mixture of DCM (80 mL) and THF (10 mL), cooled at 0 °C, was added trimethyloxonium tetrafluoroborate (1.05 g, 7.08 mmol). The resulting solution was stirred at 0 °C for 3 h and allowed to warm to RT. After 6 h, the reaction was diluted with acetonitrile (25 mL) and bis(2,4-dimethoxybenzyl)amine (2.25 g, 7.08 mmol) was added. The resulting reaction mixture was heated at 42 °C for 24 h. Upon completion, the solvent was removed *in vacuo* to yield a crude product. The crude product was purified by column chromatography (silica gel, petrol:EtOAc, 1:0 → 6:4) to yield the *title compound* as a clear oil (2.08 g, 60%); R_f = 0.28 (petrol:EtOAc, 6:4; $KMnO_4$); λ_{max} (EtOH)/nm 276.5; IR (neat) ν_{max}/cm^{-1} 3449, 1611, 1588, 1507, 1456, 1362, 1291, 1207, 1157, 1115, 1034; ^{1}H NMR (500 MHz, $CDCl_3$) δ 3.71 (6H, s, 2 × ArOC*H*$_3$), 3.79 (6H, s, 2 × ArOC*H*$_3$), 4.45 (4H, s, 2 × ArC*H*$_2$), 5.40 (1H, s, ArO*H*), 6.38 (2H, d, *J* = 2.4 Hz, H-3'), 6.43 (2H, dd, *J* = 8.4, 2.4 Hz, H-5'), 6.55 (1H, dd, *J* = 2.3, 2.3 Hz, H-2'), 6.71 – 6.64 (2H, m, H-4 and H-6), 7.13 (1H, dd, *J* = 8.2, 8.2 Hz, H-5), 7.26 (2H, d, *J* = 8.4 Hz,

H-6'); ^{13}C NMR (126 MHz, $CDCl_3$) δ 47.1 (Ar*C*H_2), 55.2 (ArO*C*H_3), 55.5 (ArO*C*H_3), 98.3 (C-3'), 104.1 (C-5'), 109.5 (C-2), 113.7 (C-4 or C-6), 114.0 (C-4 or C-6), 116.7 (C-1'), 130.2 (C-5), 131.1 (C-6'), 151.3 (C-1), 156.6 (C-3), 158.6 (C-2' or C-4'), 160.7 (C-2' or C-4'); LRMS (ES^-) *m/z* 488.4 $[M-H]^-$; HRMS (ESI) calcd for $C_{24}H_{26}NO_8S$ $[M-H]^-$ 488.1385, found 488.1374.

3-(2-Nitrophenoxy)phenyl bis(2,4-dimethoxybenzyl)sulfamate, (19)

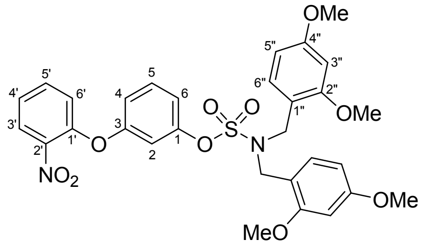

Compound **19** was synthesised according to general procedure C, using the following reagents: 3-hydroxyphenyl bis(2,4-dimethoxybenzyl)sulfamate (**18**) (1.4 g, 2.86 mmol), potassium carbonate (474 mg, 3.43 mmol), 1-fluoro-2-nitrobenzene (603 µL, 807 mg, 5.72 mmol) and *N*,*N*-dimethylformamide (20 mL). The crude brown oil was purified by column chromatography (silica gel, petrol:EtOAc, 1:0 → 3:1) to yield the *title compound* as a pale orange oil (1.40 g, 80%); R_f = 0.28 (petrol:EtOAc, 3:1); λ_{max} (EtOH)/nm 276.0; IR (neat) ν_{max}/cm^{-1} 1611, 1588, 1527, 1507, 1476, 1350, 1251, 1207, 1157, 1112, 1033; ^{1}H NMR (500 MHz, $CDCl_3$) δ 3.72 (6H, s, 2 × ArOC*H_3*), 3.78 (6H, s, 2 × ArOC*H_3*), 4.43 (4H, s, 2 × ArC*H_2*), 6.37 (2H, d, *J* = 2.3 Hz, H-3"), 6.41 (2H, dd, *J* = 8.3, 2.4 Hz, H-5"), 6.66 (1H, dd, *J* = 2.3, 2.3 Hz, H-2), 6.90 (1H, dd, *J* = 8.0, 2.0 Hz, H-6'), 7.02 – 6.95 (2H, m, H-4 or H-6 and H-4'), 7.25 – 7.21 (3H, m, H-4 or H-6 and H-6"), 7.30 (1H, dd, *J* = 8.3, 8.3 Hz, H-5), 7.54 – 7.49 (1H, m, H-5'), 7.97 (1H, dd, *J* = 8.2, 1.6 Hz, H-3'); ^{13}C NMR (126 MHz, $CDCl_3$) δ 47.2 (Ar*C*H_2), 55.2 (ArO*C*H_3), 55.5 (ArO*C*H_3), 98.3 (C-3"), 104.1 (C-5"), 113.3 (C-2), 116.5 (C-1"), 117.1 (C-6'), 118.2 (C-4 or C-6 or C-4'), 120.8 (C-4 or C-6 or C-4'), 123.8 (C-4 or C-6), 126.0 (C-3'), 130.6 (C-5), 131.1 (C-6"), 134.4 (C-5'), 141.5 (C-2'), 150.2 (C-1 or C-1'), 151.5 (C-1 or C-1'), 156.4 (C-3), 158.6 (C-2" or C-4"), 160.8 (C-2" or C-4"); HRMS (ESI) calcd for $C_{30}H_{34}N_3O_{10}S$ $[M+NH_4]^+$ 628.1959, found 628.1957.

3-(3-Nitrophenoxy)phenyl bis(2,4-dimethoxybenzyl)sulfamate, (20)

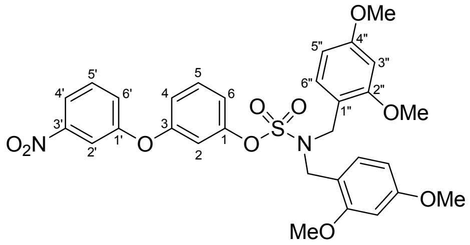

Compound **20** was synthesised according to general procedure C, using the following reagents: 3-hydroxyphenyl bis(2,4-dimethoxybenzyl)sulfamate (**18**) (1.4 g, 2.86 mmol), potassium carbonate (474 mg, 3.43 mmol), 1-fluoro-3-nitrobenzene (609 μL, 807 mg, 5.72 mmol) and *N*,*N*-dimethylformamide (20 mL). The crude brown oil was purified by column chromatography (silica gel, petrol:EtOAc, 1:0 → 3:1) to yield the *title compound* as a brown oil (1.31 g, 75%); R_f = 0.30 (petrol:EtOAc, 3:1); λ_{max} (EtOH)/nm 273.5; IR (neat) ν_{max}/cm^{-1} 1611, 1588, 1529, 1507, 1479, 1349, 1248, 1208, 1157, 1113, 1034; ^{1}H NMR (500 MHz, $CDCl_3$) δ 3.72 (6H, s, 2 × ArOCH_3), 3.78 (6H, s, 2 × ArOCH_3), 4.45 (4H, s, 2 × ArCH_2), 6.37 (2H, d, *J* = 2.4 Hz, H-3”), 6.41 (2H, dd, *J* = 8.4, 2.4 Hz, H-5”), 6.73 (1H, dd, *J* = 2.3, 2.3 Hz, H-2), 6.91 (1H, ddd, *J* = 8.3, 2.3 and 0.9 Hz, H-4 or H-6), 6.97 (1H, ddd, *J* = 8.2, 2.2 and 0.9 Hz, H-4 or H-6), 7.25 (2H, d, *J* = 8.4 Hz, H-6”), 7.30 (1H, ddd, *J* = 8.2, 2.4 and 0.9 Hz, H-6’), 7.33 (1H, dd, *J* = 8.2, 8.2 Hz, H-5), 7.50 (1H, dd, *J* = 8.2, 8.2 Hz, H-5’), 7.80 (1H, dd, *J* = 2.3, 2.3 Hz, H-2’), 7.97 (1H, ddd, *J* = 8.2, 2.1 and 0.9 Hz, H-4’); ^{13}C NMR (126 MHz, $CDCl_3$) δ 47.1 (Ar*C*H_2), 55.2 (ArO*C*H_3), 55.5 (ArO*C*H_3), 98.3 (C-3”), 104.1 (C-5”), 113.4 (C-2’), 113.7 (C-2), 116.5 (C-1”), 117.5 (C-4 or C-6), 118.3 (C-4 or C-6 or C-4’), 118.3 (C-4 or C-6 or C-4’), 124.4 (C-6’), 130.6 (C-5’), 130.8 (C-5), 131.1 (C-6”), 149.4 (C-3’), 151. 7 (C-1), 156.4 (C-3 or C-1’), 157.9 (C-3 or C-1’), 158.6 (C-2” or C-4”), 160.8 (C-2” or C-4”); HRMS (ESI) calcd for $C_{30}H_{34}N_3O_{10}S$ $[M+NH_4]^+$ 628.1959, found 628.1956.

3-(4-Nitrophenoxy)phenyl bis(2,4-dimethoxybenzyl)sulfamate, (21)

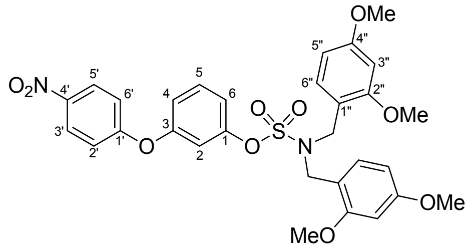

Compound **21** was synthesised according to general procedure C, using the following reagents: 3-hydroxyphenyl bis(2,4-dimethoxybenzyl)sulfamate (**18**) (1.2 g, 2.45 mmol), potassium carbonate (407 mg, 2.94 mmol), 1-fluoro-4-nitrobenzene (692 mg, 4.90 mmol) and *N,N*-dimethylformamide (20 mL). The crude brown oil was purified by column chromatography (silica gel, petrol:EtOAc, 1:0 → 8:2) to yield the *title compound* as a yellow oil (1.28 g, 85%); R_f = 0.28 (petrol:EtOAc, 8:2); λ_{max} (EtOH)/nm 282.5; IR (neat) ν_{max}/cm^{-1} 1611, 1585, 1507, 1478, 1371, 1342, 1250, 1207, 1157, 1111, 1033; ^{1}H NMR (500 MHz, $CDCl_3$) δ 3.72 (6H, s, 2 × ArOCH_3), 3.77 (6H, s, 2 × ArOCH_3), 4.44 (4H, s, 2 × ArCH_2), 6.37 (2H, d, *J* = 2.4 Hz, H-3”), 6.41 (2H, dd, *J* = 8.4, 2.4 Hz, H-5”), 6.73 (1H, dd, *J* = 2.3, 2.3 Hz, H-2), 6.96 (1H, ddd, *J* = 8.2, 2.3 and 0.8 Hz, H-4 or H-6), 7.00 (2H, d, *J* = 9.3 Hz, H-2’, 6’), 7.03 (1H, ddd, *J* = 8.3, 2.2 and 0.9 Hz, H-4 or H-6), 7.24 (2H, d, *J* = 8.4 Hz, H-6”), 7.36 (1H, dd, *J* = 8.2, 8.2 Hz, H-5), 8.21 (2H, d, *J* = 9.3 Hz, H-3’, 5’); ^{13}C NMR (126 MHz, $CDCl_3$) δ 47.1 (Ar*C*H_2), 55.3 (ArO*C*H_3), 55.5 (ArO*C*H_3), 98.3 (C-3”), 104.1 (C-5”), 114.5 (C-2), 116.4 (C-1”), 117.5 (C-2’, 6’), 118.4 (C-4 or C-6), 119.1 (C-4 or C-6), 126.1 (C-3’, 5’), 130.9 (C-5), 131.1 (C-6”), 143.1 (C-4’), 151.7 (C-1), 155.4 (C-3), 158.6 (C-2” or C-4”), 160.8 (C-2” or C-4”), 162.7 (C-1’); HRMS (ESI) calcd for $C_{30}H_{31}N_2O_{10}S$ $[M+H]^+$ 611.1691, found 611.1694.

3-(2-Aminophenoxy)phenyl bis(2,4-dimethoxybenzyl)sulfamate, (22)

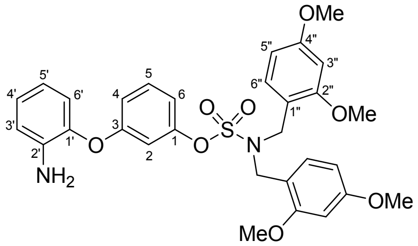

Compound **22** was synthesised according to general procedure D, using the following reagents: 3-(2-nitrophenoxy)phenyl bis(2,4-dimethoxybenzyl)sulfamate (**19**) (1.20 g, 1.96 mmol), MeOH (29.4 mL) and THF (9.8 mL). The crude brown oil was purified by column chromatography (silica gel, petrol:EtOAc, 1:0 → 7:3) to yield the *title compound* as a yellow oil (970 mg, 85%); R_f = 0.32 (petrol:EtOAc, 7:3); λ_{max} (EtOH)/nm 277.8; IR (neat) ν_{max}/cm^{-1} 3467, 3378, 1611, 1587, 1505, 1479, 1369, 1243, 1207, 1156, 1111, 1033; ^{1}H NMR (500 MHz, $CDCl_3$) δ 3.71 (6H, s, 2 × ArOCH_3), 3.79 (6H, s, 2 × ArOCH_3), 4.44 (4H, s, 2 × ArCH_2), 6.37 (2H, d, *J* = 2.3 Hz, H-3”), 6.41

(2H, dd, J = 8.3, 2.4 Hz, H-5”), 6.75 – 6.70 (2H, m, 2 × ArH), 6.89 – 6.79 (4H, m, 4 × ArH), 7.00 (1H, ddd, J = 7.7, 7.7 and 1.4 Hz, H-4’), 7.22 (1H, dd, J = 8.3, 8.3 Hz, H-5), 7.24 (2H, d, J = 8.4 Hz, H-6”); ^{13}C NMR (126 MHz, $CDCl_3$) δ 47.0 (ArCH_2), 55.2 (ArOCH_3), 55.5 (ArOCH_3), 98.3 (C-3”), 104.1 (C-5”), 111.3 (C-2), 114.9 (CH Ar), 116.2 (CH Ar), 116.6 (C-1”), 116.8 (CH Ar), 119.0 (CH Ar), 120.5 (CH Ar), 125.4 (C-4’), 130.2 (C-5), 131.0 (C-6”), 138.8 (C-1’ or C-2’), 142.6 (C-1’ or C-2’), 151.5 (C-1), 158.4 (C-3), 158.5 (C-2” or C-4”), 160.7 (C-2” or C-4”); LRMS (ES$^+$) *m/z* 581.4 $[M+H]^+$; HRMS (ESI) calcd for $C_{30}H_{33}N_2O_8S$ $[M+H]^+$ 581.1952, found 581.1947.

3-(3-Aminophenoxy)phenyl bis(2,4-dimethoxybenzyl)sulfamate, (23)

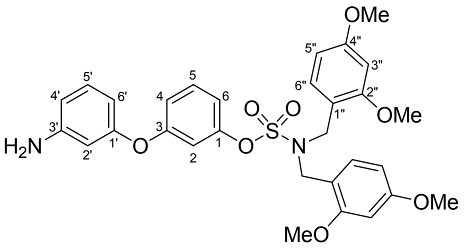

Compound **23** was synthesised according to general procedure D, using the following reagents: 3-(3-nitrophenoxy)phenyl bis(2,4-dimethoxybenzyl)sulfamate (**20**) (1.20 g, 1.96 mmol), MeOH (29.4 mL) and THF (9.8 mL). The crude brown oil was purified by column chromatography (silica gel, petrol:EtOAc, 1:0 → 65:35) to yield the *title compound* as a brown oil (994 mg, 87%); R_f = 0.29 (petrol:EtOAc, 65:35); λ_{max} (EtOH)/nm 260.2; IR (neat) ν_{max}/cm^{-1} 3467, 3380, 1610, 1586, 1507, 1478, 1464, 1367, 1207, 1148, 1110, 1033; ^{1}H NMR (500 MHz, $CDCl_3$) δ 3.71 (6H, s, 2 × ArOCH_3), 3.78 (6H, s, 2 × ArOCH_3), 4.44 (4H, s, 2 × ArCH_2), 6.32 (1H, dd, J = 2.3, 2.3 Hz, H-2’), 6.37 (2H, d, J = 2.5 Hz, H-3”), 6.40 – 6.37 (1H, m, H-4’ or H-6’), 6.41 (2H, dd, J = 8.4, 2.4 Hz, H-5”), 6.44 (1H, ddd, J = 8.1, 2.2 and 0.9 Hz, H-4’ or H-6’), 6.72 (1H, dd, J = 2.3, 2.3 Hz, H-2), 6.91 – 6.84 (2H, m, H-4 and H-6), 7.10 (1H, dd, J = 8.0, 8.0 Hz, H-5’), 7.26 – 7.21 (3H, m, H-5 and H-6”); ^{13}C NMR (126 MHz, $CDCl_3$) δ 47.1 (ArCH_2), 55.2 (ArOCH_3), 55.5 (ArOCH_3), 98.3 (C-3”), 104.1 (C-5”), 105.8 (C-2’), 109.2 (C-4’ or C-6’), 110.7 (C-4’ or C-6’), 113.0 (C-2), 116.6 (C-4 or C-6), 116.7 (C-4 or C-6), 116.9 (C-1”), 130.1 (C-5’), 130.6 (C-5), 131.1 (C-6”), 148.1 (C-3’), 151.4 (C-1), 157.9 (C-3 or C-1’), 158.1 (C-3 or C-1’), 158.5 (C-2” or C-4”), 160.7 (C-2” or C-4”); LRMS (ES$^+$) *m/z* 581.5 $[M+H]^+$; HRMS (ESI) calcd for $C_{30}H_{33}N_2O_8S$ $[M+H]^+$ 581.1952, found 581.1947.

3-(4-Aminophenoxy)phenyl bis(2,4-dimethoxybenzyl)sulfamate, (24)

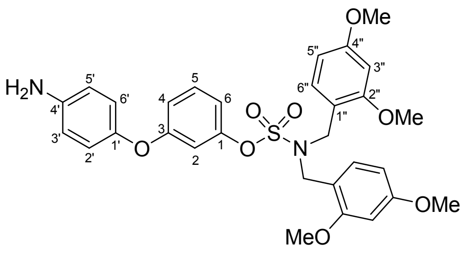

Compound **24** was synthesised according to general procedure D, using the following reagents: 3-(4-nitrophenoxy)phenyl bis(2,4-dimethoxybenzyl)sulfamate (**21**) (1.20 g, 1.96 mmol), MeOH (29.4 mL) and THF (9.8 mL). The crude brown oil was purified by column chromatography (silica gel, petrol:EtOAc, 1:0 → 55:45) to yield the *title compound* as a brown oil (982 mg, 86%); R_f = 0.31 (petrol:EtOAc, 55:45); λ_{max} (EtOH)/nm 264.5; IR (neat) ν_{max}/cm^{-1} 3453, 3378, 1611, 1588, 1506, 1478, 1366, 1247, 1205, 1157, 1111, 1033; ^{1}H NMR (500 MHz, $CDCl_3$) δ 3.71 (6H, s, 2 × ArOCH_3), 3.79 (6H, s, 2 × ArOCH_3), 4.43 (4H, s, 2 × ArCH_2), 6.37 (2H, d, *J* = 2.4 Hz, H-3”), 6.41 (2H, dd, *J* = 8.3, 2.4 Hz, H-5”), 6.71 – 6.66 (3H, m, H-2 and H-2’, 6’), 6.77 (1H, ddd, *J* = 8.3, 2.4 and 0.7 Hz, H-4 or H-6), 6.80 (1H, ddd, *J* = 8.2, 2.2 and 0.8 Hz, H-4 or H-6), 6.85 (2H, d, *J* = 8.7 Hz, H-3’, 5’), 7.19 (1H, dd, *J* = 8.2, 8.2 Hz, H-5), 7.23 (2H, d, *J* = 8.4 Hz, H-6”); ^{13}C NMR (126 MHz, $CDCl_3$) δ 47.0 (Ar*C*H_2), 55.2 (ArO*C*H_3), 55.5 (ArO*C*H_3), 98.3 (C-3”), 104.1 (C-5”), 111.3 (C-2), 115.1 (C-4 or C-6), 115.6 (C-4 or C-6), 116.6 (C-2’, 6’), 116.7 (C-1”), 121.3 (C-3’, 5’), 130.0 (C-5), 131.0 (C-6”), 142.8 (C-4’), 148.3 (C-1’), 151.4 (C-1), 158.5 (C-2” or C-4”), 159. 8 (C-3), 160.7 (C-2” or C-4”); LRMS (ES$^+$) *m/z* 581.5 [M+H]$^+$; HRMS (ESI) calcd for $C_{30}H_{33}N_2O_8S$ [M+H]$^+$ 581.1952, found 581.1945.

3-(2-Aminophenoxy)phenyl sulfamate, (25)

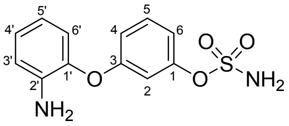

Compound **25** was synthesised according to general procedure B, using the following reagents: 3-(2-aminophenoxy)phenyl bis(2,4-dimethoxybenzyl)sulfamate (**22**) (200 mg, 0.34 mmol), DCM (3.1 mL) and TFA (0.4 mL). The crude product was purified by column chromatography (silica gel, petrol:EtOAc, 1:0 → 7:3) to yield the *title compound* as a pale yellow solid (87 mg, 90%); R_f = 0.34 (petrol:EtOAc, 7:3); m.p.

91-5-93.5 °C; λ_{max} (EtOH)/nm 277.2; IR (neat) ν_{max}/cm^{-1} 3482, 3390, 3345, 3263, 1622, 1604, 1586, 1503, 1478, 1344, 1184, 1107; ^{1}H NMR (500 MHz, DMSO-d_6) δ 4.93 (2H, s, ArN*H*$_2$), 6.57 (1H, ddd, *J* = 7.7, 7.7 and 1.6 Hz, H-5'), 6.84 – 6.78 (3H, m, 3 × Ar*H*), 6.85 (1H, dd, *J* = 8.0, 1.5 Hz, H-3' or H-6'), 6.99 – 6.93 (2H, m, 2 × Ar*H*), 7.40 – 7.34 (1H, m, H-5), 8.02 (2H, s, ArOSO$_2$N*H*$_2$); ^{13}C NMR (126 MHz, DMSO-d_6) δ 110.4 (C-2), 114.2 (C-3' or C-6'), 115.7 (C-4 or C-6), 116.0 (C-4 or C-6), 116.4 (C-5'), 120.9 (C-3' or C-6'), 125.6 (C-4'), 130.4 (C-5), 140.6 (C-1' or C-2'), 140.7 (C-1' or C-2'), 151.0 (C-1), 158.5 (C-3); LRMS (ES$^-$) *m/z* 279.2 [M-H]$^-$; HRMS (ESI) calcd for $C_{12}H_{11}N_2O_4S$ [M-H]$^-$ 279.0445, found 279.0436.

3-(3-Aminophenoxy)phenyl sulfamate, (26)

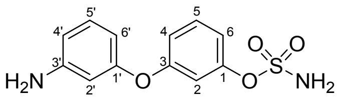

Compound **26** was synthesised according to general procedure B, using the following reagents: 3-(3-aminophenoxy)phenyl bis(2,4-dimethoxybenzyl)sulfamate (**23**) (200 mg, 0.34 mmol), DCM (3.1 mL) and TFA (0.4 mL). The crude product was purified by column chromatography (silica gel, petrol:EtOAc, 1:0 → 65:35) to yield the *title compound* as a pale brown solid (80 mg, 82%); R_f = 0.31 (petrol:EtOAc, 65:35); m.p. 79.5-81.5 °C; λ_{max} (EtOH)/nm 279.2; IR (neat) ν_{max}/cm^{-1} 3438, 3357, 3321, 1607, 1586, 1478, 1363, 1250, 1181, 1146, 1115; ^{1}H NMR (500 MHz, DMSO-d_6) δ 5.26 (2H, s, ArN*H*$_2$), 6.17 (1H, dd, *J* = 8.0, 2.3 Hz, H-4' or H-6'), 6.24 (1H, dd, *J* = 2.2, 2.2 Hz, H-2'), 6.37 (1H, dd, *J* = 8.1, 1.9 Hz, H-4' or H-6'), 6.87 (1H, dd, *J* = 2.3, 2.3 Hz, H-2), 6.93 (1H, dd, *J* = 8.3, 2.3 Hz, H-4 or H-6), 7.06 – 6.97 (2H, m, H-4 or H-6 and H-5'), 7.42 (1H, dd, *J* = 8.2, 8.2 Hz, H-5'), 8.02 (2H, s, ArOSO$_2$N*H*$_2$); ^{13}C NMR (126 MHz, DMSO-d_6) δ 104.2 (C-2'), 106.1 (C-4' or C-6'), 109.9 (C-4' or C-6'), 112.0 (C-2), 116.1 (C-4 or C-6), 116.4 (C-4 or C-6), 130.2 (C-5'), 130.5 (C-5), 150.6 (C-1 or C-3'), 151.0 (C-1 or C-3'), 156.7 (C-3 or C-1'), 158.0 (C-3 or C-1'); LRMS (ES$^-$) *m/z* 279.2 [M-H]$^-$; HRMS (ESI) calcd for $C_{12}H_{11}N_2O_4S$ [M-H]$^-$ 279.0445, found 279.0438.

3-(4-Aminophenoxy)phenyl sulfamate, (27)

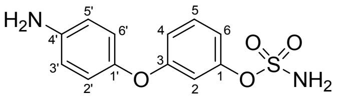

Compound **27** was synthesised according to general procedure B, using the following reagents: 3-(4-aminophenoxy)phenyl bis(2,4-dimethoxybenzyl)sulfamate (**24**) (200 mg, 0.34 mmol), DCM (3.1 mL) and TFA (0.4 mL). The crude product was purified by column chromatography (silica gel, petrol:EtOAc, 1:0 → 6:4) to yield the *title compound* as a brown solid (63 mg, 65%); R_f = 0.30 (petrol:EtOAc, 6:4); m.p. 133.5-135.5 °C; λ_{max} (EtOH)/nm 242.6; IR (neat) ν_{max}/cm^{-1} 3410, 3327, 3296, 1600, 1507, 1475, 1366, 1249, 1173, 1158, 1109; ^{1}H NMR (500 MHz, DMSO-d_6) δ 5.04 (2H, s, ArNH_2), 6.60 (2H, d, J = 8.8 Hz, H-2', 6'), 6.74 (1H, dd, J = 2.3, 2.3 Hz, H-2), 6.85 – 6.76 (3H, m, H-4 or H-6 and H-3', 5'), 6.91 (1H, dd, J = 8.1, 2.3 Hz, H-4 or H-6), 7.36 (1H, dd, J = 8.2, 8.2 Hz, H-5), 7.99 (2H, s, ArOSO$_2$NH_2); ^{13}C NMR (126 MHz, DMSO-d_6) δ 110.2 (C-2), 114.3 (C-4 or C-6), 114.9 (C-3', 5'), 115.3 (C-4 or C-6), 121.1 (C-2', 6'), 130.4 (C-5), 144.7 (C-1' or C-4'), 145.9 (C-1' or C-4'), 151.0 (C-1), 159.9 (C-3); LRMS (ES$^-$) *m/z* 279.2 [M-H]$^-$; HRMS (ESI) calcd for $C_{12}H_{11}N_2O_4S$ [M-H]$^-$: 279.0445; found 279.0437.

2'-Acetamido-[1,1'-biphenyl]-3-yl bis(2,4-dimethoxybenzyl)sulfamate, (28)

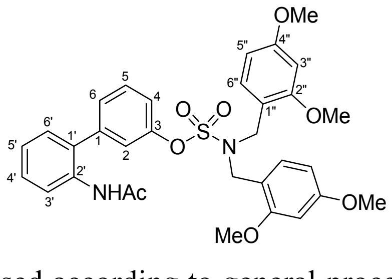

Compound **28** was synthesised according to general procedure E, using the following reagents: 2'-amino-[1,1'-biphenyl]-3-yl bis(2,4-dimethoxybenzyl)sulfamate (**5**) (180 mg, 0.32 mmol), triethylamine (65 mg, 89 μL, 0.64 mmol), acetic anhydride (39 mg, 36 μL, 0.38 mmol) and DCM (3.2 mL). The crude product was purified by column chromatography (silica gel, petrol:EtOAc, 1:0 → 1:1) to yield the *title compound* as a pale yellow oil (168 mg, 87%); R_f = 0.32 (petrol:EtOAc, 1:1); No λ_{max} (EtOH)/nm; IR (neat) ν_{max}/cm^{-1} 1680, 1611, 1586, 1507, 1368, 1290, 1208, 1156, 1132, 1032, 887; ^{1}H NMR (500 MHz, CDCl$_3$) δ 2.03 (3H, s, CH_3CO), 3.71 (6H, s, 2 × ArOCH_3), 3.76 (6H, s, 2 × ArOCH_3), 4.49 (4H, s, 2 × ArCH_2), 6.38 (2H, d, J = 2.4 Hz,

H-3”), 6.44 (2H, dd, J = 8.4, 2.4 Hz, H-5”), 7.00 (1H, dd, J = 2.0 Hz, H-2), 7.25 – 7.12 (4H, m, 4 × Ar*H*), 7.28 (2H, d, J = 8.4 Hz, H-6”), 7.38 (1H, ddd, J = 8.5, 7.0 and 2.0 Hz, H-4’ or H-5’), 7.44 (1H, dd, J = 7.9, 7.9 Hz, H-5), 8.28 (1H, d, J = 8.2 Hz, H-3’); ^{13}C NMR (126 MHz, $CDCl_3$) δ 24.7 (CH_3CO), 47.3 (ArCH_2), 55.3 (ArOCH_3), 55.5 (ArOCH_3), 98.4 (C-3”), 104.1 (C-5”), 116.6 (C-1”), 121.7 (CH Ar), 122.0 (C-3’), 123.3 (C-2), 124.4 (CH Ar), 127.5 (CH Ar), 128.9 (C-4’ or C-5’), 130.0 (CH Ar), 130.5 (C-5), 130.9 (C_q Ar), 131.1 (C-6”), 134.9 (C_q Ar), 139.8 (C_q Ar), 150.7 (C-3), 158.6 (C-2” or C-4”), 160.8 (C-2” or C-4”), 168.8 (COCH_3); LRMS (ES^-) *m/z* 605.3 $[M-H]^-$; HRMS (ESI) calcd for $C_{32}H_{33}N_2O_8S$ $[M-H]^-$ 605.1963, found 605.1965.

3'-Acetamido-[1,1'-biphenyl]-3-yl bis(2,4-dimethoxybenzyl)sulfamate, (29)

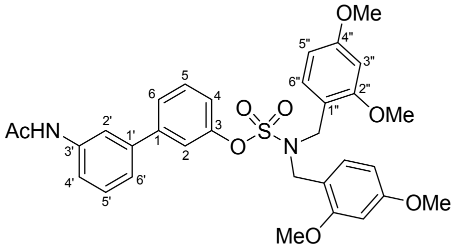

Compound **29** was synthesised according to general procedure E, using the following reagents: 3'-amino-[1,1'-biphenyl]-3-yl bis(2,4-dimethoxybenzyl)sulfamate (**6**) (180 mg, 0.32 mmol), triethylamine (65 mg, 89 µL, 0.64 mmol), acetic anhydride (39 mg, 36 µL, 0.38 mmol) and DCM (3.2 mL). The crude product was purified by column chromatography (silica gel, petrol:EtOAc, 1:0 → 4:6) to yield the *title compound* as a pale yellow oil (172 mg, 89%); R_f = 0.29 (petrol:EtOAc, 4:6); λ_{max} (EtOH)/nm 235.0; IR (neat) ν_{max}/cm^{-1} 1669, 1610, 1589, 1507, 1368, 1207, 1156, 1133, 1035; ^{1}H NMR (500 MHz, $CDCl_3$) δ 2.24 (3H, s, CH_3CO), 3.67 (6H, s, 2 × ArOCH_3), 3.81 (6H, s, 2 × ArOCH_3), 4.43 (4H, s, 2 × ArCH_2), 6.39 (2H, d, J = 2.4 Hz, H-3”), 6.47 (2H, dd, J = 8.4, 2.4 Hz, H-5”), 6.83 (1H, dd, J = 2.0, 2.0 Hz, H-2), 7.17 (1H, ddd, J = 8.2, 2.4 and 1.1 Hz, Ar*H*), 7.25 – 7.22 (2H, m, 2 × Ar*H*), 7.32 (2H, d, J = 8.4 Hz, H-6”), 7.35 (1H, dd, J = 7.9, 7.9 Hz, H-5 or H-5’), 7.39 (1H, dd, J = 7.8, 7.8 Hz, H-5 or H-5’), 7.45 – 7.41 (1H, m, H-6), 7.59 (1H, s, N*H*Ac), 7.81 (1H, ddd, J = 8.3, 1.4 and 1.4 Hz, H-4’); ^{13}C NMR (126 MHz, $CDCl_3$) δ 24.8 (CH_3CO), 47.2 (ArCH_2), 55.2 (ArOCH_3), 55.6 (ArOCH_3), 98.5 (C-3”), 104.2 (C-5”), 117.0 (C-1”), 118.3 (CH Ar), 119.5 (C-4’), 120.5 (C-2), 121.3 (CH Ar), 123.0 (CH Ar), 125.1 (C-6), 129.7 (C-5 or C-5’), 129.9 (C-5 or C-5’), 131.4 (C-6”), 138.6 (C_q Ar), 140.6 (C_q Ar),

142.1 (C_q Ar), 150.9 (C-3), 158.7 (C-2” or C-4”), 160.6 (C-2” or C-4”), 168.5 (*C*OCH$_3$); LRMS (ES$^-$) *m/z* 605.4 [M-H]$^-$; HRMS (ESI) calcd for $C_{32}H_{33}N_2O_8S$ [M-H]$^-$ 605.1963, found 605.1961.

4'-Acetamido-[1,1'-biphenyl]-3-yl bis(2,4-dimethoxybenzyl)sulfamate, (30)

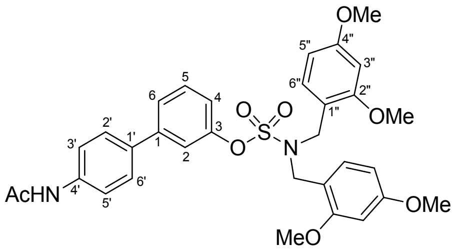

Compound **30** was synthesised according to general procedure E, using the following reagents: 4'-amino-[1,1'-biphenyl]-3-yl bis(2,4-dimethoxybenzyl)sulfamate (**7**) (150 mg, 0.27 mmol), triethylamine (54 mg, 74 µL, 0.53 mmol), acetic anhydride (33 mg, 30 µL, 0.32 mmol) and DCM (2.7 mL). The crude product was purified by column chromatography (silica gel, petrol:EtOAc, 1:0 $\rightarrow$ 2:8) to yield the *title compound* as an orange oil (142 mg, 88%); R_f = 0.34 (petrol:EtOAc, 2:8); λ_{max} (EtOH)/nm 277.0; IR (neat) ν_{max}/cm^{-1} 1666, 1599, 1532, 1506, 1379, 1207, 1155, 1125, 1038, 893; ^{1}H NMR (500 MHz, CDCl$_3$) δ 2.20 (3H, s, C*H*$_3$CO), 3.70 (6H, s, 2 × ArOC*H*$_3$), 3.78 (6H, s, 2 × ArOC*H*$_3$), 4.47 (4H, s, 2 × ArC*H*$_2$), 6.37 (2H, d, *J* = 2.4 Hz, H-3”), 6.43 (2H, dd, *J* = 8.3, 2.4 Hz, H-5”), 7.15 – 7.08 (2H, m, H-2 and H-4), 7.28 (2H, d, *J* = 8.4 Hz, H-6”), 7.32 (1H, s, N*H*Ac), 7.34 (1H, dd, *J* = 7.9, 7.9 Hz, H-5), 7.42 – 7.38 (1H, m, H-6), 7.44 (2H, d, *J* = 8.5 Hz, H-2’, 6’), 7.57 (2H, d, *J* = 8.5 Hz, H-3’, 5’); ^{13}C NMR (126 MHz, CDCl$_3$) δ 24.8 (*C*H$_3$CO), 47.1 (Ar*C*H$_2$), 55.2 (ArO*C*H$_3$), 55.5 (ArO*C*H$_3$), 98.3 (C-3”), 104.1 (C-5”), 116.7 (C-1”), 120.1 (C-2’, 6’), 120.3 (C-2 or C-4), 120.6 (C-2 or C-4), 124.8 (C-6), 127.8 (C-3’, 5’), 129.9 (C-5), 131.2 (C-6”), 135.8 (C_q Ar), 137.8 (C_q Ar), 142.2 (C_q Ar), 150.9 (C-3), 158.6 (C-2” or C-4”), 160.7 (C-2” or C-4”), 168.4 (*C*OCH$_3$); LRMS (ES$^-$) *m/z* 605.4 [M-H]$^-$; HRMS (ESI) calcd for $C_{32}H_{33}N_2O_8S$ [M-H]$^-$ 605.1963, found 605.1961.

3-(2-Acetamidophenoxy)phenyl bis(2,4-dimethoxybenzyl)sulfamate, (31)

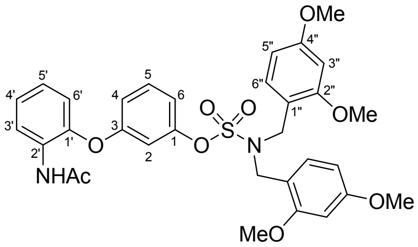

Compound **31** was synthesised according to general procedure E, using the following reagents: 3-(2-aminophenoxy)phenyl bis(2,4-dimethoxybenzyl)sulfamate (**22**) (250 mg, 0.43 mmol), triethylamine (87 mg, 120 μL, 0.86 mmol), acetic anhydride (53 mg, 49 μL, 0.52 mmol) and DCM (4.3 mL). The crude product was purified by column chromatography (silica gel, petrol:EtOAc, 1:0 → 6:4) to yield the *title compound* as a cleqr oil (233 mg, 87%); $R_f = 0.30$ (petrol:EtOAc, 6:4); λ_{max} (EtOH)/nm 277.2; IR (neat) ν_{max}/cm^{-1} 1683, 1609, 1589, 1507, 1476, 1440, 1368, 1252, 1207, 1157, 1115, 1034; ^1H NMR (500 MHz, $CDCl_3$) δ 2.19 (3H, s, CH_3CO), 3.71 (6H, s, 2 × ArOCH_3), 3.78 (6H, s, 2 × ArOCH_3), 4.46 (4H, s, 2 × ArCH_2), 6.38 (2H, d, *J* = 2.4 Hz, H-3”), 6.41 (2H, dd, *J* = 8.4, 2.4 Hz, H-5”), 6.75 (1H, dd, *J* = 2.3, 2.3 Hz, H-2), 6.82 (1H, dd, *J* = 8.1, 1.5 Hz, H-6’), 6.85 (1H, ddd, *J* = 8.3, 2.5 and 0.9 Hz, H-4 or H-6), 6.93 (1H, ddd, *J* = 8.2, 2.3 and 1.0 Hz, H-4 or H-6), 7.00 (1H, ddd, *J* = 7.8, 7.8 and 1.7 Hz, H-4’), 7.13 (1H, ddd, *J* = 7.7, 7.7 and 1.4 Hz, H-5’), 7.25 (2H, d, *J* = 8.4 Hz, H-6”), 7.28 (1H, dd, *J* = 8.3, 8.3 Hz, H-5), 7.69 (1H, s, ArN*H*Ac), 8.44 (1H, dd, *J* = 8.2, 1.7 Hz, H-3’); ^{13}C NMR (126 MHz, $CDCl_3$) δ 25.0 (CH_3CO), 47.1 (ArCH_2), 55.2 (ArOCH_3), 55.5 (ArOCH_3), 98.3 (C-3”), 104.1 (C-5”), 113.1 (C-2), 116.5 (C-4 or C-6), 116.6 (C-1”), 117.6 (C-4 or C-6), 117.7 (C-6’), 121.2 (C-3’), 124.2 (C-4 or C-6), 124.5 (C-4’), 129.9 (C-2’), 130.5 (C-5), 131.1 (C-6”), 145.2 (C-1’), 151.6 (C-1), 157.1 (C-3), 158.6 (C-2” or C-4”), 160.8 (C-2” or C-4”), 168.5 ($COCH_3$); LRMS (ES^-) *m/z* 621.4 $[M-H]^-$; HRMS (ESI) calcd for $C_{32}H_{33}N_2O_9S$ $[M-H]^-$ 621.1912, found 621.1893.

3-(3-Acetamidophenoxy)phenyl bis(2,4-dimethoxybenzyl)sulfamate, (32)

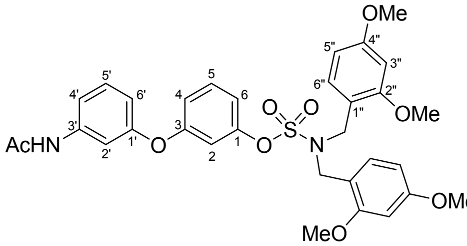

Compound **32** was synthesised according to general procedure E, using the following reagents: 3-(4-aminophenoxy)phenyl bis(2,4-dimethoxybenzyl)sulfamate (**23**) (250 mg, 0.43 mmol), triethylamine (87 mg, 120 μL, 0.86 mmol), acetic anhydride (53 mg, 49 μL, 0.52 mmol) and DCM (4.3 mL). The crude product was purified by column chromatography (silica gel, petrol:EtOAc, 1:0 → 45:55) to yield the *title compound* as a clear oil (236 mg, 88%); $R_f = 0.27$ (petrol:EtOAc, 45:55); λ_{max} (EtOH)/nm 278.7; IR (neat) ν_{max}/cm^{-1} 1670, 1589, 1541, 1507, 1478, 1368, 1257, 1207, 1157, 1111, 1033; ^{1}H NMR (500 MHz, $CDCl_3$) δ 2.11 (3H, s, CH_3CO), 3.70 (6H, s, 2 × ArOCH_3), 3.78 (6H, s, 2 × ArOCH_3), 4.44 (4H, s, 2 × ArCH_2), 6.37 (2H, d, *J* = 2.4 Hz, H-3"), 6.41 (2H, dd, *J* = 8.4, 2.4 Hz, H-5"), 6.70 (1H, dd, *J* = 2.3, 2.3 Hz, H-2), 6.75 – 6.71 (1H, m, H-6'), 6.86 (1H, ddd, *J* = 8.2, 2.4 and 0.9 Hz, H-4 or H-6), 6.89 (1H, ddd, *J* = 8.3, 2.3 and 0.9 Hz, H-4 or H-6), 7.10 (1H, dd, *J* = 2.3, 2.3 Hz, H-2'), 7.30 – 7.21 (4H, m, H-5 and H-5' and H-6"), 7.32 (1H, s, ArN*H*Ac), 7.38 – 7.34 (1H, m, H-4'); ^{13}C NMR (126 MHz, $CDCl_3$) δ 24.7 (*C*H_3CO), 47.1 (Ar*C*H_2), 55.3 (ArO*C*H_3), 55.5 (ArO*C*H_3), 98.3 (C-3"), 104.1 (C-5"), 110.4 (C-2'), 113.0 (C-2), 114.7 (C-6'), 115.1 (C-4'), 116.6 (C-1"), 117.0 (C-4 or C-6), 117.0 (C-4 or C-6), 130.3 (C-5 or C-5'), 130.3 (C-5 or C-5'), 131.0 (C-6"), 139.5 (C-3'), 151.4 (C-1), 157.3 (C-3 or C-1'), 157.8 (C-3 or C-1'), 158.5 (C-2" or C-4"), 160.7 (C-2" or C-4"), 168.4 (*C*OCH_3); LRMS (ES$^-$) *m/z* 621.4 [M-H]$^-$; HRMS (ESI) calcd for $C_{32}H_{33}N_2O_9S$ [M-H]$^-$ 621.1912, found 621.1894.

3-(4-Acetamidophenoxy)phenyl bis(2,4-dimethoxybenzyl)sulfamate, (33)

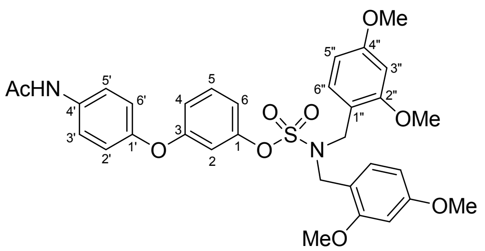

Compound **33** was synthesised according to general procedure E, using the following reagents: 3-(4-aminophenoxy)phenyl bis(2,4-dimethoxybenzyl)sulfamate (**24**) (250 mg, 0.43 mmol), triethylamine (87 mg, 120 μL, 0.86 mmol), acetic anhydride (53 mg, 49 μL, 0.52 mmol) and DCM (4.3 mL). The crude product was purified by column chromatography (silica gel, petrol:EtOAc, 1:0 → 35:65) to yield the *title compound* as a white solid (231 mg, 86%); R_f = 0.30 (petrol:EtOAc, 35:65); m.p. 49.5-51.5 °C; λ_{max} (EtOH)/nm 251.0; IR (neat) ν_{max}/cm^{-1} 1667, 1611, 1589, 1505, 1478, 1368, 1249, 1206, 1157, 1111, 1034; ^{1}H NMR (500 MHz, $CDCl_3$) δ 2.16 (3H, s, CH_3CO), 3.70 (6H, s, 2 × ArOCH_3), 3.78 (6H, s, 2 × ArOCH_3), 4.44 (4H, s, 2 × ArCH_2), 6.36 (2H, d, *J* = 2.3 Hz, H-3”), 6.41 (2H, dd, *J* = 8.4, 2.4 Hz, H-5”), 6.70 (1H, dd, *J* = 2.3, 2.3 Hz, H-2), 6.82 (1H, dd, *J* = 8.3, 2.3 Hz, H-4 or H-6), 6.86 (1H, dd, *J* = 8.2, 2.1 Hz, H-4 or H-6), 6.95 (2H, d, *J* = 8.9 Hz, H-2’, 6’), 7.25 – 7.20 (3H, m, H-5 and H-6”), 7.31 (1H, s, ArN*H*Ac), 7.46 (2H, d, *J* = 8.9 Hz, H-3’, 5’); ^{13}C NMR (126 MHz, $CDCl_3$) δ 24.6 (*C*H_3CO), 47.1 (Ar*C*H_2), 55.2 (ArO*C*H_3), 55.5 (ArO*C*H_3), 98.3 (C-3”), 104.1 (C-5”), 112.3 (C-2), 116.2 (C-4 or C-6), 116.5 (C-4 or C-6), 116.6 (C-1”), 120.0 (C-2’, 6’), 121.8 (C-3’, 5’), 130.2 (C-5), 131.0 (C-6”), 134.0 (C-4’), 151.4 (C-1 or C-1’), 152.8 (C-1 or C-1’), 158.5 (C-3), 158.5 (C-2” or C-”), 160.7 (C-2” or C-4”), 168.4 (*C*OCH_3); LRMS (ES$^-$) *m/z* 621.5 [M-H]$^-$; HRMS (ESI) calcd for $C_{32}H_{33}N_2O_9S$ [M-H]$^-$ 621.1912, found 621.1892.

2'-Acetamido-[1,1'-biphenyl]-3-yl sulfamate, (34)

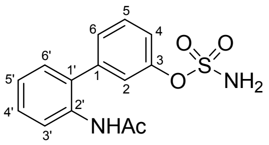

Compound **34** was synthesised according to general procedure B, using the following reagents: 2'-acetamido-[1,1'-biphenyl]-3-yl bis(2,4-dimethoxybenzyl)sulfamate (**28**) (130 mg, 0.21 mmol), DCM (1.8 mL) and TFA (0.2 mL). The crude product was purified by column chromatography (silica gel, petrol:EtOAc, 1:0 → 4:6) to yield the *title compound* as a white solid (57 mg, 86%); R_f = 0.30 (petrol:EtOAc, 4:6); m.p. 164.5-166.5 °C; No λ_{max} (EtOH)/nm; IR (neat) ν_{max}/cm^{-1} 3297, 3038, 1616, 1589, 1382, 1366, 1197, 1147, 892; ^{1}H NMR (500 MHz, DMSO-d_6) δ 1.90 (3H, s, CH_3CO), 7.44 – 7.17 (6H, m, 6 × Ar*H*), 7.65 – 7.44 (2H, m, 2 × Ar*H*), 8.00 (2H, s, ArOSO_2NH_2), 9.26 (1H, s, ArN*H*Ac); ^{13}C NMR (126 MHz, DMSO-d_6) δ 23.1 (*C*H_3CO), 121.1 (CH

Ar), 122.4 (CH Ar), 125.9 (CH Ar), 127.0 (CH Ar), 128.1 (CH Ar), 129.8 (CH Ar), 130.2 (CH Ar), 134.9 (C_q Ar), 135.1 (C_q Ar), 140.6 (C_q Ar), 150.0 (C-3), 168.8 (*C*OCH_3); LRMS (ES^+) *m/z* 307.2 $[M+H]^+$; HRMS (ESI) calcd for $C_{14}H_{15}N_2O_4S$ $[M+H]^+$ 307.0747, found 307.0750.

3'-Acetamido-[1,1'-biphenyl]-3-yl sulfamate, (35)

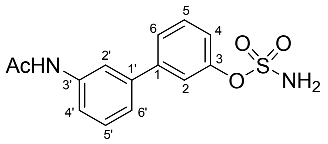

Compound **35** was synthesised according to general procedure B, using the following reagents: 3'-acetamido-[1,1'-biphenyl]-3-yl bis(2,4-dimethoxybenzyl)sulfamate (**29**) (130 mg, 0.21 mmol), DCM (1.8 mL) and TFA (0.2 mL). The crude product was purified by column chromatography (silica gel, petrol:EtOAc, 1:0 → 3:7) to yield the *title compound* as a white solid (60 mg, 91%); R_f = 0.30 (petrol:EtOAc, 3:7); m.p. 152.0-154.0 °C; λ_{max} (EtOH)/nm 242.0; IR (neat) ν_{max}/cm^{-1} 3356, 3219, 1653, 1554, 1364, 1186, 1147, 863; ^1H NMR (500 MHz, DMSO-d_6) δ 2.07 (3H, s, CH_3CO), 7.31 – 7.25 (1H, m, Ar*H*), 7.34 (1H, d, *J* = 7.7 Hz, Ar*H*), 7.41 (1H, dd, *J* = 7.8, 7.8 Hz, H-5), 7.51 – 7.46 (1H, m, H-2), 7.64 – 7.53 (3H, m, 3 × Ar*H*), 7.91 (1H, dd, *J* = 1.9, 1.9 Hz, H-2'), 8.05 (2H, s, ArOSO_2NH_2), 10.07 (1H, s, ArN*H*Ac); ^{13}C NMR (126 MHz, DMSO-d_6) δ 24.0 (*C*H_3CO), 117.3 (C-2'), 118.6 (CH Ar), 120.3 (C-2), 121.3 (CH Ar), 121.5 (CH Ar), 124.8 (CH Ar), 129.4 (C-5), 130.3 (CH Ar), 139.5 (C_q Ar), 140.0 (C_q Ar), 142.0 (C_q Ar), 150.7 (C-3), 168.5 (*C*OCH_3); LRMS (ES^+) *m/z* 307.2 $[M+H]^+$; HRMS (ESI) calcd for $C_{14}H_{15}N_2O_4S$ $[M+H]^+$ 307.0747, found 307.0753.

4'-Acetamido-[1,1'-biphenyl]-3-yl sulfamate, (36)

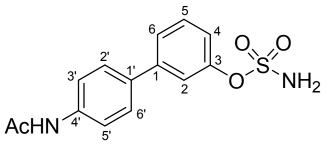

Compound **36** was synthesised according to general procedure B, using the following reagents: 4'-acetamido-[1,1'-biphenyl]-3-yl bis(2,4-dimethoxybenzyl)sulfamate (**30**) (120 mg, 0.20 mmol), DCM (1.8 mL) and TFA (0.2 mL). The crude product was purified by column chromatography (silica gel, petrol:EtOAc, 1:0 → 1:9) to yield the *title compound* as a pale orange solid (51 mg, 84%); R_f = 0.32 (petrol:EtOAc, 1:9); m.p.

175.5-177.5 °C; λ_{max} (EtOH)/nm 276.5; IR (neat) ν_{max}/cm^{-1} 3362, 3291, 2976, 1656, 1595, 1528, 1358, 1182, 1139, 910; ^{1}H NMR (500 MHz, DMSO-d_6) δ 2.07 (3H, s, CH_3CO), 7.23 (1H, dd, J = 7.8, 2.5 Hz, H-4), 7.56 – 7.47 (2H, m, H-2 and H-5), 7.65 – 7.58 (3H, m, H-6 and H-2', 6'), 7.69 (2H, d, J = 8.3 Hz, H-3', 5'), 8.02 (2H, s, ArOSO$_2$NH_2), 10.06 (1H, s, ArN*H*Ac); ^{13}C NMR (126 MHz, DMSO-d_6) δ 24.1 (*C*H$_3$CO), 119.3 (C-3', 5'), 119.9 (C-2), 120.7 (C-4), 124.4 (C-6), 127.1 (C-2', 6'), 130.2 (C-5), 133.4 (C-4'), 139.3 (C-1 or C-1'), 141.6 (C-1 or C-1'), 150.7 (C-3), 168.4 (*C*OCH$_3$); LRMS (ES$^+$) *m/z* 307.2 [M+H]$^+$; HRMS (ESI) calcd for $C_{14}H_{13}N_2O_4S$ [M-H]$^-$ 305.0602, found 305.0593.

3-(2-Acetamidophenoxy)phenyl sulfamate, (37)

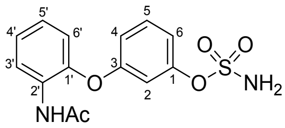

Compound **37** was synthesised according to general procedure B, using the following reagents: 3-(2-acetamidophenoxy)phenyl bis(2,4-dimethoxybenzyl)sulfamate (**31**) (200 mg, 0.32 mmol), DCM (2.9 mL) and TFA (0.3 mL). The crude product was purified by column chromatography (silica gel, petrol:EtOAc, 1:0 → 65:35) to yield the *title compound* as a white solid (88 mg, 85%); R_f = 0.29 (petrol:EtOAc, 65:35); m.p. 142.0-144.0 °C; No λ_{max} (EtOH)/nm; IR (neat) ν_{max}/cm^{-1} 3383, 3299, 1665, 1609, 1594, 1539, 1477, 1453, 1366, 1254, 1190, 1123; ^{1}H NMR (500 MHz, DMSO-d_6) δ 2.00 (3H, s, CH_3CO), 6.93 – 6.88 (2H, m, 2 × Ar*H*), 6.99 (1H, dd, J = 7.8, 1.8 Hz, H-6'), 7.06 – 7.02 (1H, m, Ar*H*), 7.19 – 7.08 (2H, m, 2 × Ar*H*), 7.43 (1H, dd, J = 8.5, 8.5 Hz, H-5), 7.94 (1H, d, J = 7.4 Hz, H-3'), 8.04 (2H, s, ArOSO$_2$NH_2), 9.48 (1H, s, ArN*H*Ac); ^{13}C NMR (126 MHz, DMSO-d_6) δ 23.5 (*C*H$_3$CO), 112.1 (CH Ar), 116.0 (CH Ar), 116.8 (CH Ar), 119.4 (C-6'), 124.2 (CH Ar), 124.3 (CH Ar), 125.0 (CH Ar), 130.1 (C-2'), 130.6 (C-5), 146.8 (C-1'), 151.0 (C-1), 157.6 (C-3), 168.7 (*C*OCH$_3$); LRMS (ES$^-$) *m/z* 321.2 [M-H]$^-$; HRMS (ESI) calcd for $C_{14}H_{13}N_2O_5S$ [M-H]$^-$ 321.0551, found 321.0539.

3-(3-Acetamidophenoxy)phenyl sulfamate, (38)

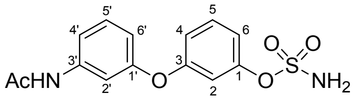

Compound **38** was synthesised according to general procedure B, using the following reagents: 3-(3-acetamidophenoxy)phenyl bis(2,4-dimethoxybenzyl)sulfamate (**32**) (200 mg, 0.32 mmol), DCM (2.9 mL) and TFA (0.3 mL). The crude product was purified by column chromatography (silica gel, petrol:EtOAc, 1:0 → 1:1) to yield the *title compound* as a white solid (98 mg, 94%); R_f = 0.28 (petrol:EtOAc, 1:1); m.p. 134.0-136.0 °C; λ_{max} (EtOH)/nm 275.0; IR (neat) ν_{max}/cm^{-1} 3361, 3290, 3215, 3085, 1651, 1600, 1549, 1478, 1436, 1379, 1264, 1182, 1144, 1114; ^{1}H NMR (500 MHz, DMSO-d_6) δ 2.02 (3H, s, CH_3CO), 6.76 – 6.71 (1H, m, H-6'), 6.91 (1H, dd, *J* = 2.3, 2.3 Hz, H-2), 6.96 (1H, ddd, *J* = 8.3, 2.4 and 0.9 Hz, H-4 or H-6), 7.05 (1H, ddd, *J* = 8.2, 2.2 and 0.9 Hz, H-4 or H-6), 7.35 – 7.30 (2H, m, H-5 and H-4'), 7.43 (1H, dd, *J* = 1.6, 1.6 Hz, H-2'), 7.45 (1H, dd, *J* = 8.3, 8.3 Hz, H-5'), 8.03 (2H, s, ArOSO$_2$NH_2), 10.04 (1H, s, ArN*H*Ac); ^{13}C NMR (126 MHz, DMSO-d_6) δ 24.0 (*C*H$_3$CO), 109.5 (C-2'), 112.3 (C-2), 113.5 (C-6'), 114.4 (C-4'), 116.3 (C-4 or C-6), 117.0 (C-4 or C-6), 130.2 (C-5 or C-5'), 130.8 (C-5 or C-5'), 141.0 (C-3'), 151.1 (C-1), 156.0 (C-1'), 157.5 (C-3), 168.5 (*C*OCH$_3$); LRMS (ES$^-$) *m/z* 643.3 [2M-H]$^-$; HRMS (ESI) calcd for $C_{14}H_{13}N_2O_5S$ [M-H]$^-$ 321.0551, found 321.0541.

3-(4-Acetamidophenoxy)phenyl sulfamate, (39)

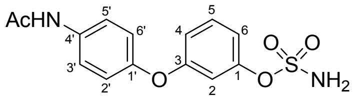

Compound **39** was synthesised according to general procedure B, using the following reagents: 3-(4-acetamidophenoxy)phenyl bis(2,4-dimethoxybenzyl)sulfamate (**33**) (200 mg, 0.32 mmol), DCM (2.9 mL) and TFA (0.3 mL). The crude product was purified by column chromatography (silica gel, petrol:EtOAc, 1:0 → 45:55) to yield the *title compound* as a white solid (96 mg, 92%); R_f = 0.29 (petrol:EtOAc, 45:55); m.p. 108.0-110.0 °C; λ_{max} (EtOH)/nm 250.8; IR (neat) ν_{max}/cm^{-1} 3309, 3178, 3076, 1635, 1596, 1526, 1506, 1477, 1371, 1250, 1178, 1161, 1117; ^{1}H NMR (500 MHz, DMSO-d_6) δ 2.04 (3H, s, CH_3CO), 6.84 (1H, dd, *J* = 2.3, 2.3 Hz, H-2), 6.91 (1H, ddd, *J* = 8.3, 2.4 and 0.9 Hz, H-4 or H-6), 7.00 (1H, ddd, *J* = 8.2, 2.2 and 0.9 Hz, H-4 or H-6), 7.04 (2H, d, *J* = 8.9 Hz, H-2', 6'), 7.42 (1H, dd, *J* = 8.2, 8.2 Hz, H-5), 7.62 (2H, d, *J* = 9.0 Hz,

H-3’, 5’), 8.01 (2H, s, ArOSO$_2$N*H*$_2$), 9.99 (1H, s, ArN*H*Ac); ^{13}C NMR (126 MHz, DMSO-*d*$_6$) δ 23.9 (*C*H$_3$CO), 111.4 (C-2), 115.5 (C-4 or C-6), 116.4 (C-4 or C-6), 119.9 (C-2’, 6’), 120.6 (C-3’, 5’), 130.7 (C-5), 135.9 (C-4’), 150.5 (C-1 or C-1’), 151.0 (C-1 or C-1’), 158.4 (C-3), 168.1 (*C*OCH$_3$); LRMS (ES$^-$) *m/z* 321.2 [M-H]$^-$; HRMS (ESI) calcd for $C_{14}H_{13}N_2O_5S$ [M-H]$^-$ 321.0551, found 321.0543.

2,2,2-Trichlroethyl chlorosulfate, (41)

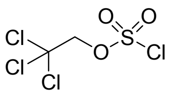

To sulfuryl chloride (6.76 mL, 83.5 mmol) in Et$_2$O (40 mL), cooled at -78 °C, was added dropwise over 35 min a solution of 2,2,2-trichloroethanol (8.00 mL, 12.5 g, 83.5 mmol) and pyridine (6.76 mL, 6.61 g, 83.5 mmol) in Et$_2$O (40 mL). The resulting mixture was stirred at -78 °C for 1 h and for an additional 2 h at RT. The mixture was then filtered to remove the pyridinium hydrochloride salt by-product and concentrated *in vacuo*. The crude liquid was purified by distillation under reduced pressure using a Kügelrohr distillation apparatus to yield the *title compound* as a clear liquid (17.3 g, 83%); IR (neat) ν_{max}/cm^{-1} 1414, 1189, 985, 870; ^{1}H NMR (500 MHz, CDCl$_3$) δ 4.92 (s, 2H, C*H*$_2$OSO$_3$Cl); ^{13}C NMR (126 MHz, CDCl$_3$) δ 81.3 (*C*H$_2$OSO$_3$Cl), 91.4 (CH$_2$*C*Cl$_3$); ^{1}H NMR, ^{13}C NMR and IR data were identical to literature data[2]

2,2,2-Trichloroethyl 2-methyl-1*H*-imidazole-1-sulfonate, (42)

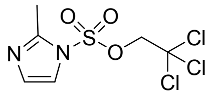

To a suspension of 2-methylimidazole (10.4 g, 126 mmol) in THF (60 mL), cooled at 0 °C, was added dropwise a solution of 2,2,2-trichloroethyl chlorosulfate (**41**) (4.68 mL, 8.7 g, 35.1 mmol) in THF (40 mL). The resulting solution was stirred at 0 °C for 1 h and allowed to warm to RT. After 16 h, the remaining 2-methylimidazole was filtered off and washed with THF (50 mL), and the filtrate was concentrated *in vacuo*. The crude residue was dissolved in EtOAc (80 mL), washed with water and brine (50 mL, respectively), dried over MgSO$_4$ and concentrated *in vacuo*. The crude product was purified by column chromatography (silica gel, petrol:EtOAc, 1:0 → 7:3) to yield the *title compound* as a white amorphous solid (9.75 g, 95%); R_f = 0.29 (petrol:EtOAc, 7:3; KMnO$_4$); m.p. 64.5-66.5 °C (lit. 53.0-55.0 °C)[3]; IR (neat) ν_{max}/cm^{-1} 3167, 1556, 1420,

1197, 1182, 1155, 980; ^{1}H NMR (500 MHz, $CDCl_3$) δ 2.68 (3H, s, CH_3), 4.64 (2H, s, CH_2CCl_3), 6.95 (1H, d, J = 1.8 Hz, H-4 or H-5), 7.32 (1H, d, J = 1.8 Hz, H-4 or H-5); ^{13}C NMR (126 MHz, $CDCl_3$) δ 15.1 (CH_3), 80.1 ($\mathit{C}H_2CCl_3$), 91.9 ($CH_2\mathit{C}Cl_3$), 120.3 (C-4 or C-5), 128.4 (C-4 or C-5), 146.6 (C-2); LRMS (ES^+) *m/z* 293.1 $[M(^{35}Cl^{35}Cl^{35}Cl)+H]^+$, 295.1 $[M(^{37}Cl^{35}Cl^{35}Cl)+H]^+$, 297.1 $[M(^{37}Cl^{37}Cl^{35}Cl)+H]^+$; ^{1}H NMR, ^{13}C NMR and LRMS data were identical to literature data.[3]

2,3-Dimethyl-1-((2,2,2-trichloroethoxy)sulfonyl)-1*H*-imidazol-3-ium tetrafluoroborate, (44)

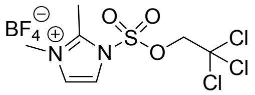

To 2,2,2-trichloroethyl 2-methyl-1*H*-imidazole-1-sulfonate (**43**) (9.8 g, 33.4 mmol) in DCM (100 mL), cooled at 0 °C, was added trimethyloxonium tetrafluoroborate (4.94 g, 33.4 mmol). The resulting solution was stirred at 0 °C for 1 h and allowed to warm to RT. After 20 h, the reaction was cooled in an ice bath. Cold petrol (120 mL) was added to the mixture, leading to the precipitation of **44**. The white precipitate was filtered off, washed with cold petrol (60 mL) and dry under high vacuum. The white fluffy solid (11.4 g, 86%) was used in the next step without further purification; m.p. 118.5-120.5 °C; IR (neat) ν_{max}/cm^{-1} 3150, 1601, 1531, 1438, 1230, 1208, 1162, 1050, 1027, 973; ^{1}H NMR (500 MHz, MeOD) δ 2.93 (3H, s, CH_3), 3.94 (3H, s, $NC\mathit{H}_3$), 5.34 (2H, s, CH_2CCl_3), 7.74 (1H, d, J = 2.5 Hz, H-4 or H-5), 8.08 (1H, d, J = 2.5 Hz, H-4 or H-5); ^{13}C NMR (126 MHz, MeOD) δ 11.8 (CH_3), 36.6 ($N\mathit{C}H_3$), 83.3 ($\mathit{C}H_2CCl_3$), 93.0 ($CH_2\mathit{C}Cl_3$), 122.1 (C-4 or C-5), 124.9 (C-4 or C-5), 150.0 (C-2); ^{19}F NMR (471 MHz, MeOD) δ -154.50 (BF_4^-); LRMS (ES^+) *m/z* 307.1 $[M(^{35}Cl^{35}Cl^{35}Cl)+H]^+$, 309.1 $[M(^{37}Cl^{35}Cl^{35}Cl)+H]^+$, 311.1 $[M(^{37}Cl^{37}Cl^{35}Cl)+H]^+$; ^{1}H NMR, ^{13}C NMR, ^{19}F NMR and LRMS data were identical to literature data.[3]

3'-(((2,2,2-Trichloroethoxy)sulfonyl)amino)-[1,1'-biphenyl]-3-yl bis(2,4-dimethoxybenzyl)sulfamate, (45)

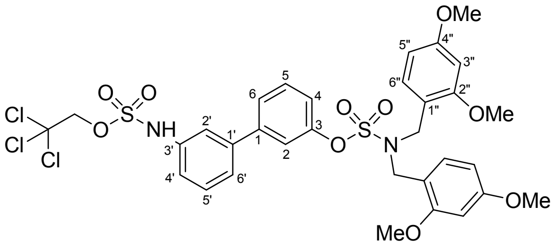

Compound **45** was synthesised according to general procedure F, using the following reagents: 3'-amino-[1,1'-biphenyl]-3-yl bis(2,4-dimethoxybenzyl)sulfamate (**6**) (550 mg, 0.97 mmol), 2,3-dimethyl-1-((2,2,2-trichloroethoxy)sulfonyl)-1*H*-imidazol-3-ium tetrafluoroborate (**44**) (1.16 g, 2.92 mmol) and THF (9.8 mL). The crude product was purified by column chromatography (silica gel, petrol:EtOAc, 1:0 $\rightarrow$ 65:35) to yield the *title compound* as a pale yellow oil (567 mg, 75%); R_f = 0.31 (petrol:EtOAc, 65:35); No λ_{max} (EtOH)/nm; IR (neat) ν_{max}/cm^{-1} 3264, 1610, 1589, 15074, 1360, 1208, 1176, 1157, 1037; ^{1}H NMR (500 MHz, $CDCl_3$) δ 3.66 (6H, s, 2 × ArOCH_3), 3.85 (6H, s, 2 × ArOCH_3), 4.42 (4H, s, 2 × ArCH_2), 4.70 (2H, s, CH_2CCl$_3$), 6.41 (2H, d, *J* = 2.4 Hz, H-3”), 6.51 (2H, dd, *J* = 8.4, 2.4 Hz, H-5”), 6.56 (1H, dd, *J* = 2.0, 2.0 Hz, H-2’), 7.01 (1H, dd, *J* = 2.0, 2.0 Hz, H-2), 7.26 (1H, ddd, *J* = 8.0, 2.2 and 1.1 Hz, Ar*H*), 7.35 (2H, d, *J* = 8.4 Hz, H-6”), 7.39 – 7.36 (2H, m, 2 × Ar*H*), 7.46 – 7.41 (3H, m, 3 × Ar*H*), 7.71 (1H, s, ArN*H*SO$_3$); ^{13}C NMR (126 MHz, $CDCl_3$) δ 47.3 (Ar*C*H$_2$), 55.3 (ArO*C*H$_3$), 55.8 (ArO*C*H$_3$), 79.1 (*C*H$_2$CCl$_3$), 93.2 (CH$_2$*C*Cl$_3$), 98.7 (C-3”), 104.4 (C-5”), 117.3 (C-1”), 119.7 (C-2), 119.9 (CH Ar), 120.4 (C-2’), 121.7 (CH Ar), 124.3 (CH Ar), 124.9 (CH Ar), 130.1 (CH Ar), 130.2 (CH Ar), 131.6 (C-6”), 136.2 (C-3’), 141.1 (C-1 or C-1’), 141.2 (C-1 or C-1’), 150.9 (C-3), 158.8 (C-2” or C-4”), 160.5 (C-2” or C-4”); HRMS (ESI) calcd for $C_{32}H_{37}Cl_3N_3O_{10}S_2$ $[M(^{35}Cl^{35}Cl^{35}Cl)+H]^+$ 792.0980, found 792.0989.

4'-(((2,2,2-Trichloroethoxy)sulfonyl)amino)-[1,1'-biphenyl]-3-yl bis(2,4-dimethoxy benzyl)sulfamate, (46)

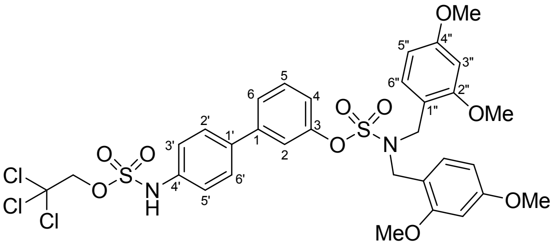

Compound **46** was synthesised according to general procedure F, using the following reagents: 4'-amino-[1,1'-biphenyl]-3-yl bis(2,4-dimethoxybenzyl)sulfamate (**7**) (550 mg, 0.97 mmol), 2,3-dimethyl-1-((2,2,2-trichloroethoxy)sulfonyl)-1*H*-imidazol-3-ium tetrafluoroborate (**44**) (1.16 g, 2.92 mmol) and THF (9.8 mL). The crude product was purified by column chromatography (silica gel, petrol:EtOAc, 1:0 → 6:4) to yield the *title compound* as a yellow oil (620 mg, 82%); R_f = 0.32 (petrol:EtOAc, 6:4); λ_{max} (EtOH)/nm 272.5; IR (neat) ν_{max}/cm^{-1} 1611, 1588, 1507, 1367, 1208, 1178, 1132, 1038; ^{1}H NMR (500 MHz, $CDCl_3$) δ 3.71 (6H, s, 2 × ArOCH_3), 3.78 (6H, s, 2 × ArOCH_3), 4.47 (4H, s, 2 × ArCH_2), 4.68 (2H, s, CH_2CCl$_3$), 6.38 (2H, d, *J* = 2.4 Hz, H-3”), 6.44 (2H, dd, *J* = 8.4, 2.4 Hz, H-5”), 7.14 – 7.10 (2H, m, H-2 and H-4), 7.28 (2H, d, *J* = 8.4 Hz, H-6”), 7.30 (2H, d, *J* = 8.6 Hz, H-3’, 5’), 7.38 – 7.34 (1H, m, H-5), 7.41 – 7.38 (1H, m, H-6), 7.48 (2H, d, *J* = 8.6 Hz, H-2’, 6’); ^{13}C NMR (126 MHz, $CDCl_3$) δ 47.1 (Ar*C*H$_2$), 55.3 (ArO*C*H$_3$), 55.5 (ArO*C*H$_3$), 79.2 (*C*H$_2$CCl$_3$), 93.1 (CH$_2$*C*Cl$_3$), 98.4 (C-3”), 104.1 (C-5”), 116.7 (C-1”), 120.5 (C-2 or C-4), 121.1 (C-2 or C-4), 121.6 (C-3’, 5’), 124.9 (C-6), 128.4 (C-2’, 6’), 130.1 (C-5), 131.2 (C-6”), 135.0 (C-4’), 137.7 (C-1 or C-1’), 141.5 (C-1 or C-1’), 151.0 (C-3), 158.6 (C-2” or C-4”), 160.8 (C-2” or C-4”); HRMS (ESI) calcd for $C_{32}H_{34}Cl_3N_2O_{10}S_2$ [M(^{35}Cl^{35}Cl^{35}Cl)+H]$^+$ 775.0715, found 775.0721.

3-(2-(((2,2,2-Trichloroethoxy)sulfonyl)amino)phenoxy)phenyl bis(2,4-dimethoxybenzyl) sulfamate, (47)

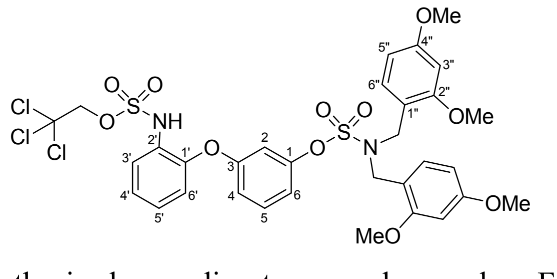

Compound **47** was synthesised according to general procedure F, using the following reagents: 3-(2-aminophenoxy)phenyl bis(2,4-dimethoxybenzyl)sulfamate (**22**) (50 mg, 0.09 mmol), 2,3-dimethyl-1-((2,2,2-trichloroethoxy)sulfonyl)-1*H*-imidazol-3-ium tetrafluoroborate (**44**) (102 mg, 0.26 mmol) and THF (1 mL). The crude product was purified by column chromatography (silica gel, petrol:EtOAc, 1:0 → 3:1) to yield the *title compound* as a clear oil (10 mg, 15%); R_f = 0.31 (petrol:EtOAc, 3:1); λ_{max} (EtOH)/nm 277.2; IR (neat) ν_{max}/cm^{-1} 1611, 1589, 1507, 1368, 1255, 1208, 1174, 1157, 1116, 1036; ^{1}H NMR (500 MHz, $CDCl_3$) δ 3.72 (6H, s, 2 × ArOCH_3), 3.79 (6H,

s, 2 × ArOCH_3), 4.45 (4H, s, 2 × ArCH_2), 4.68 (2H, s, CH_2CCl$_3$), 6.39 (2H, d, *J* = 2.4 Hz, H-3”), 6.42 (2H, dd, *J* = 8.3, 2.4 Hz, H-5”), 6.67 (1H, dd, *J* = 2.3, 2.3 Hz, H-2), 6.83 (1H, dd, *J* = 8.1, 1.5 Hz, H-6’), 6.88 (1H, ddd, *J* = 8.3, 2.4 and 0.9 Hz, H-4 or H-6), 6.97 (1H, ddd, *J* = 8.3, 2.3 and 0.9 Hz, H-4 or H-6), 7.09 (1H, ddd, *J* = 8.0, 7.6 and 1.7 Hz, H-4 or H-5), 7.14 (1H, ddd, *J* = 7.9, 1.5 and 1.5 Hz, H-4’ or H-5’), 7.24 (2H, d, *J* = 8.3 Hz, H-6”), 7.31 (1H, dd, *J* = 8.3, 8.3 Hz, H-5), 7.65 (1H, dd, *J* = 8.0, 1.6 Hz, H-3’); ^{13}C NMR (126 MHz, CDCl$_3$) δ 47.1 (Ar*C*H$_2$), 55.3 (ArO*C*H$_3$), 55.6 (ArO*C*H$_3$), 79.1 (*C*H$_2$CCl$_3$), 93.0 (CH$_2$*C*Cl$_3$), 98.3 (C-3”), 104.1 (C-5”), 113.3 (C-2), 116.6 (C-1”), 116.9 (C-4 or C-6), 117.6 (C-6’), 118.2 (C-4 or C-6), 121.2 (C-3’), 124.4 (C-4’ or C-5’), 126.1 (C-4’ or C-5’), 126.8 (C-2’), 130.6 (C-5), 131.1 (C-6”), 146.6 (C-1’), 151.6 (C-1), 156.1 (C-3), 158.6 (C-2” or C-4”), 160.7 (C-2” or C-4”); LRMS (ES$^-$) *m/z* 789.3 [M(^{35}Cl^{35}Cl^{35}Cl)-H]$^-$, 791.3 [M(^{37}Cl^{35}Cl^{35}Cl)-H]$^-$; HRMS (ESI) calcd for $C_{32}H_{32}Cl_3N_2O_{11}S_2$ [M(^{35}Cl^{35}Cl^{35}Cl)-H]$^-$ 789.0519, found 789.0494.

3-(3-(((2,2,2-Trichloroethoxy)sulfonyl)amino)phenoxy)phenyl bis(2,4-dimethoxybenzyl) sulfamate, (48)

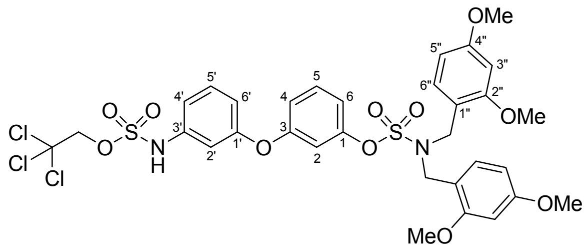

Compound **48** was synthesised according to general procedure F, using the following reagents: 3-(3-aminophenoxy)phenyl bis(2,4-dimethoxybenzyl)sulfamate (**23**) (450 mg, 0.77 mmol), 2,3-dimethyl-1-((2,2,2-trichloroethoxy)sulfonyl)-1*H*-imidazol-3-ium tetrafluoroborate (**44**) (919 mg, 2.32 mmol) and THF (7.7 mL). The crude product was purified by column chromatography (silica gel, petrol:EtOAc, 1:0 → 7:3) to yield the *title compound* as a yellow oil (480 mg, 78%); R_f = 0.31 (petrol:EtOAc, 7:3); λ_{max} (EtOH)/nm 277.4; IR (neat) ν_{max}/cm^{-1} 1611, 1590, 1507, 1478, 1367, 1266, 1208, 1175, 1149, 1112, 1037; ^{1}H NMR (500 MHz, CDCl$_3$) δ 3.71 (6H, s, 2 × ArOCH_3), 3.79 (6H, s, 2 × ArOCH_3), 4.44 (4H, s, 2 × ArCH_2), 4.62 (2H, s, CH_2CCl$_3$), 6.38 (2H, d, *J* = 2.4 Hz, H-3”), 6.42 (2H, dd, *J* = 8.3, 2.4 Hz, H-5”), 6.69 (1H, dd, *J* = 2.3, 2.3 Hz, H-2), 6.81 (1H, dd, *J* = 2.2, 2.2 Hz, H-2’), 6.85 – 6.82 (1H, m, H-6’), 6.90 – 6.86 (1H, m, H-4 or H-6), 6.91 (1H, ddd, *J* = 8.2, 2.3 and 0.9 Hz, H-4 or H-6), 7.05 (1H, ddd, *J* = 8.2, 2.3 and 1.0 Hz, H-4’), 7.17 (1H, s, ArN*H*SO$_3$), 7.24 (2H,

d, J = 8.4 Hz, H-6”), 7.27 (1H, dd, J = 8.2, 8.2 Hz, H-5 or H-5’), 7.32 (1H, dd, J = 8.1, 8.1 Hz, H-5 or H-5’); ^{13}C NMR (126 MHz, CDCl$_3$) δ 47.2 (Ar*C*H$_2$), 55.3 (ArO*C*H$_3$), 55.6 (ArO*C*H$_3$), 79.0 (*C*H$_2$CCl$_3$), 93.1 (CH$_2$*C*Cl$_3$), 98.4 (C-3”), 104.2 (C-5”), 111.2 (C-2’), 113.3 (C-2), 115.7 (C-4’), 116.0 (C-6’), 116.6 (C-1”), 117.3 (C-4 or C-6), 117.5 (C-4 or C-6), 130.5 (C-5 or C-5’), 131.0 (C-5 or C-5’), 131.1 (C-6”), 137.0 (C-3’), 151.4 (C-1), 157.1 (C-3 or C-1’), 157.9 (C-3 or C-1’), 158.5 (C-2” or C-4”), 160.7 (C-2” or C-4”); LRMS (ES$^-$) *m/z* 789.2 [M(^{35}Cl^{35}Cl^{35}Cl)-H]$^-$, 791.2 [M(^{37}Cl^{35}Cl^{35}Cl)-H]$^-$; HRMS (ESI) calcd for $C_{32}H_{32}Cl_3N_2O_{11}S_2$ [M(^{35}Cl^{35}Cl^{35}Cl)-H]$^-$ 789.0519, found 789.0493.

3-(4-(((2,2,2-Trichloroethoxy)sulfonyl)amino)phenoxy)phenyl bis(2,4-dimethoxybenzyl) sulfamate, (49)

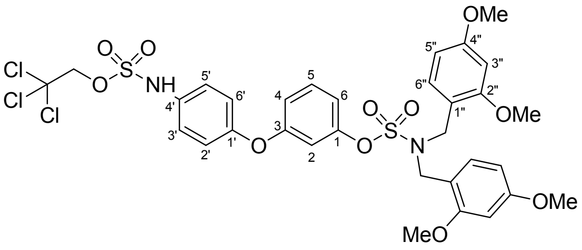

Compound **49** was synthesised according to general procedure F, using the following reagents: 3-(4-aminophenoxy)phenyl bis(2,4-dimethoxybenzyl)sulfamate (**24**) (450 mg, 0.77 mmol), 2,3-dimethyl-1-((2,2,2-trichloroethoxy)sulfonyl)-1*H*-imidazol-3-ium tetrafluoroborate (**44**) (919 mg, 2.32 mmol) and THF (7.7 mL). The crude product was purified by column chromatography (silica gel, petrol:EtOAc, 1:0 → 6:4) to yield the *title compound* as a yellow oil (553 mg, 90%); R_f = 0.32 (petrol:EtOAc, 65:35); ^{1}H NMR (500 MHz, CDCl$_3$) δ 3.71 (6H, s, 2 × ArOC*H*$_3$), 3.79 (6H, s, 2 × ArOC*H*$_3$), 4.44 (4H, s, 2 × ArC*H*$_2$), 4.65 (2H, s, C*H*$_2$CCl$_3$), 6.37 (2H, d, J = 2.3 Hz, H-3”), 6.41 (2H, dd, J = 8.4, 2.4 Hz, H-5”), 6.71 (1H, dd, J = 2.3, 2.3 Hz, H-2), 6.85 (1H, ddd, J = 8.3, 2.4 and 0.8 Hz, H-4 or H-6), 6.87 (1H, s, ArN*H*SO$_3$), 6.90 (1H, ddd, J = 8.2, 2.3 and 0.9 Hz, H-4 or H-6), 6.99 (2H, d, J = 8.8 Hz, H-2’, 6’), 7.30 – 7.20 (5H, m, H-5 and H-3’, 5’ and H-6”); ^{13}C NMR (126 MHz, CDCl$_3$) δ 47.1 (Ar*C*H$_2$), 55.2 (ArO*C*H$_3$), 55.5 (ArO*C*H$_3$), 79.0 (*C*H$_2$CCl$_3$), 93.2 (CH$_2$*C*Cl$_3$), 98.3 (C-3”), 104.1 (C-5”), 112.9 (C-2), 116.5 (C-1”), 116.9 (C-4 or C-6), 117.3 (C-4 or C-6), 119.9 (C-2’, 6’), 124.8 (C-3’, 5’), 130.4 (C-5), 130.6 (C-4’), 131.0 (C-5), 151.5 (C-1), 155.3 (C-1’), 157.6 (C-3), 158.6 (C-2” or C-4”), 160.7 (C-2” or C-4”); IR (neat) ν_{max}/cm^{-1} 1612, 1589, 1505, 1478, 1368, 1254, 1208, 1175, 1112, 1037; λ_{max} (EtOH)/nm 277.6; LRMS

(ES$^-$) *m/z* 789.3 [M(^{35}Cl^{35}Cl^{35}Cl)-H]$^-$, 791.3 [M(^{37}Cl^{35}Cl^{35}Cl)-H]$^-$; HRMS (ESI) calcd for $C_{32}H_{32}Cl_3N_2O_{11}S_2$ [M(^{35}Cl^{35}Cl^{35}Cl)-H]$^-$ 789.0519, found 789.0497.

2,2,2-Trichloroethyl (3'-(sulfamoyloxy)-[1,1'-biphenyl]-3-yl)sulfamate, (50)

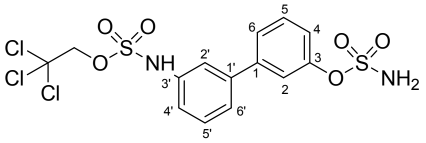

Compound **50** was synthesised according to general procedure B, using the following reagents: 3'-(((2,2,2-trichloroethoxy)sulfonyl)amino)-[1,1'-biphenyl]-3-yl bis(2,4-dimethoxy benzyl)sulfamate (**45**) (550 mg, 0.71 mmol), DCM (6.4 mL) and TFA (0.7 mL). The crude product was purified by column chromatography (silica gel, petrol:EtOAc, 1:0 → 7:3) to yield the *title compound* as a pale orange oil (290 mg, 86%); R_f = 0.29 (petrol:EtOAc, 7:3); λ_{max} (EtOH)/nm 248.0; IR (neat) ν_{max}/cm^{-1} 3281, 1606, 1576, 1476, 1370, 1175, 1146, 1087, 1014; ^{1}H NMR (500 MHz, $CDCl_3$) δ 4.68 (2H, s, C*H*$_2$CCl$_3$), 5.12 (2H, s, NH$_2$), 7.10 (1H, s, ArN*H*SO$_3$), 7.30 – 7.26 (1H, m, Ar*H*), 7.35 – 7.31 (1H, m, Ar*H*), 7.54 – 7.39 (6H, m, 6 × Ar*H*); ^{13}C NMR (126 MHz, $CDCl_3$) δ 79.1 (*C*H$_2$CCl$_3$), 93.1 (CH$_2$*C*Cl$_3$), 119.8 (CH Ar), 120.5 (CH Ar), 120.9 (CH Ar), 121.5 (CH Ar), 124.8 (CH Ar), 126.1 (CH Ar), 130.4 (CH Ar), 130.5 (CH Ar), 136.1 (C-3'), 141.2 (C-1 or C-1'), 142.2 (C-1 or C-1'), 150.6 (C-3); LRMS (ES$^-$) *m/z* 473.1 [M(^{35}Cl^{35}Cl^{35}Cl)-H]$^-$, 475.1 [M(^{37}Cl^{35}Cl^{35}Cl)-H]$^-$, 477.2 [M(^{37}Cl^{37}Cl^{35}Cl)-H]$^-$; HRMS (ESI) calcd for $C_{14}H_{12}Cl_3N_2O_6S_2$ [M(^{35}Cl^{35}Cl^{35}Cl)-H]$^-$ 472.9208, found 472.9202.

2,2,2-Trichloroethyl (3'-(sulfamoyloxy)-[1,1'-biphenyl]-4-yl)sulfamate, (51)

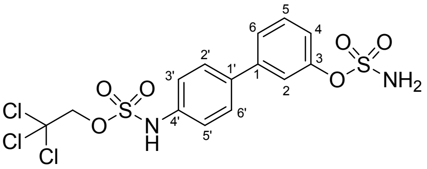

Compound **51** was synthesised according to general procedure B, using the following reagents: 4'-(((2,2,2-trichloroethoxy)sulfonyl)amino)-[1,1'-biphenyl]-3-yl bis(2,4-dimethoxy benzyl)sulfamate (**46**) (600 mg, 0.77 mmol), DCM (7.0 mL) and TFA (0.7 mL). The crude product was purified by column chromatography (silica gel, petrol:EtOAc, 1:0 → 65:35) to yield the *title compound* as a pale yellow oil (335 mg, 91%); R_f = 0.28 (petrol:EtOAc, 65:35); λ_{max} (EtOH)/nm 264.0; IR (neat) ν_{max}/cm^{-1} 3361, 3265, 1605, 1574, 1522, 1483, 1427, 1363, 1170, 1145, 1014; ^{1}H NMR

(500 MHz, DMSO-d_6) δ 4.88 (2H, s, CH_2CCl$_3$), 7.26 (1H, ddd, J = 7.9, 2.3 and 1.0 Hz, H-4 or H-6), 7.34 (2H, d, J = 8.6 Hz, H-3', 5'), 7.57 – 7.50 (2H, m, H-2 and H-5), 7.64 – 7.58 (1H, m, H-4 or H-6), 7.70 (2H, d, J = 8.7 Hz, H-2', 6'), 8.03 (2H, s, ArOSO$_2$NH_2), 11.15 (1H, s, ArNHSO$_3$); ^{13}C NMR (126 MHz, DMSO-d_6) δ 77.8 (CH$_2$CCl$_3$), 93.5 (CH$_2$$CCl_3$), 120.1 (C-2), 120.3 (C-3', 5'), 121.1 (C-4 or C-6), 124.6 (C-4 or C-6), 127.7 (C-2', 6'), 130.3 (C-5), 135.1 (C-1' or C-4'), 136.5 (C-1' or C-4'), 141.1 (C-1), 150.7 (C-3); LRMS (ES$^-$) *m/z* 473.1 [M(^{35}Cl^{35}Cl^{35}Cl)-H]$^-$, 475.1 [M(^{37}Cl^{35}Cl^{35}Cl)-H]$^-$, 477.1 [M(^{37}Cl^{37}Cl^{35}Cl)-H]$^-$; HRMS (ESI) calcd for $C_{14}H_{12}Cl_3N_2O_6S_2$ [M(^{35}Cl^{35}Cl^{35}Cl)-H]$^-$ 472.9208, found 472.9198.

2,2,2-Trichloroethyl (3-(3-(sulfamoyloxy)phenoxy)phenyl)sulfamate, (52)

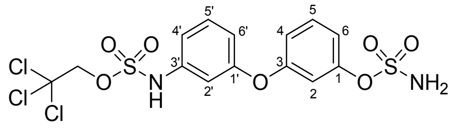

Compound **52** was synthesised according to general procedure B, using the following reagents: 3-(3-(((2,2,2-trichloroethoxy)sulfonyl)amino)phenoxy)phenyl bis(2,4-dimethoxybenzyl) sulfamate (**48**) (450 mg, 0.57 mmol), DCM (5.2 mL) and TFA (0.6 mL). The crude product was purified by column chromatography (silica gel, petrol:EtOAc, 1:0 → 7:3) to yield the *title compound* as a clear brown oil (240 mg, 86%); R_f = 0.33 (petrol:EtOAc, 7:3); λ_{max} (EtOH)/nm 277.4; IR (neat) ν_{max}/cm^{-1} 3285, 1593, 1478, 1363, 1257, 1181, 1146, 1112, 1087; ^{1}H NMR (500 MHz, DMSO-d_6) δ 4.85 (2H, s, CH_2CCl$_3$), 6.84 (1H, ddd, J = 8.2, 2.4 and 0.9 Hz, ArH), 6.94 – 6.91 (2H, m, H-2 and H-2'), 6.96 (1H, ddd, J = 8.2, 2.4 and 0.9 Hz, ArH), 7.09 – 7.02 (2H, m, 2 × ArH), 7.40 (1H, dd, J = 8.2, 8.2 Hz, H-5 or H-5'), 7.46 (1H, dd, J = 8.2, 8.2 Hz, H-5 or H-5'), 8.04 (2H, s, ArOSO$_2$NH_2), 11.16 (1H, s, ArNHSO$_3$); ^{13}C NMR (126 MHz, DMSO-d_6) δ 77.8 (CH$_2$CCl$_3$), 93.4 (CH$_2$$CCl_3$), 110.1 (C-2 or C-2'), 112.6 (C-2 or C-2'), 114.7 (CH Ar), 115.0 (CH Ar), 116.4 (CH Ar), 117.4 (CH Ar), 130.9 (C-5 or C-5'), 130.9 (C-5 or C-5'), 138.3 (C-3'), 151.1 (C-1), 156.5 (C-3 or C-1'), 157.0 (C-3 or C-1'); LRMS (ES$^-$) *m/z* 489.1 [M(^{35}Cl^{35}Cl^{35}Cl)-H]$^-$, 491.2 [M(^{37}Cl^{35}Cl^{35}Cl)-H]$^-$; HRMS (ESI) calcd for $C_{14}H_{12}Cl_3N_2O_7S_2$ [M(^{35}Cl^{35}Cl^{35}Cl)-H]$^-$ 488.9157, found 488.9152.

2,2,2-Trichloroethyl (4-(3-(sulfamoyloxy)phenoxy)phenyl)sulfamate, (53)

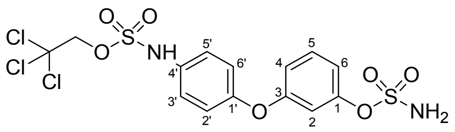

Compound **53** was synthesised according to general procedure B, using the following reagents: 3-(4-(((2,2,2-trichloroethoxy)sulfonyl)amino)phenoxy)phenyl bis(2,4-dimethoxybenzyl) sulfamate (**49**) (500 mg, 0.63 mmol), DCM (5.7 mL) and TFA (0.7 mL). The crude product was purified by column chromatography (silica gel, petrol:EtOAc, 1:0 → 65:35) to yield the *title compound* as a clear brown oil (276 mg, 89%); R_f = 0.30 (petrol:EtOAc, 6:4); λ_{max} (EtOH)/nm 234.6; IR (neat) ν_{max}/cm^{-1} 3278, 1596, 1504, 1478, 1364, 1252, 1171, 1111, 1012; ^{1}H NMR (500 MHz, DMSO-d_6) δ 4.86 (2H, s, CH_2CCl$_3$),6.89 (1H, dd, *J* = 2.3, 2.3 Hz, H-2), 6.93 (1H, ddd, *J* = 8.3, 2.4 and 1.0 Hz, H-4 or H-6), 7.04 (1H, ddd, *J* = 8.2, 2.3 and 0.9 Hz, H-4 or H-4), 7.11 (2H, d, *J* = 8.9 Hz, H-2', 6'), 7.29 (2H, d, *J* = 8.9 Hz, H-3', 5'), 7.45 (1H, dd, *J* = 8.2, 8.2 Hz, H-5), 8.03 (2H, s, ArOSO$_2$NH_2), 10.95 (1H, s, ArNHSO$_3$); ^{13}C NMR (126 MHz, DMSO-d_6) δ 77.7 (CH$_2$CCl$_3$), 93.6 (CH$_2$$CCl_3$), 112.0 (C-2), 115.9 (C-4 or C-6), 117.0 (C-4 or C-6), 120.2 (C-2', 6'), 122.7 (C-3', 5'), 130.8 (C-5), 132.5 (C-4'), 151.1 (C-1), 152.7 (C-1'), 157.7 (C-3); LRMS (ES$^-$) *m/z* 489.1 [M(^{35}Cl^{35}Cl^{35}Cl)-H]$^-$, 491.2 [M(^{37}Cl^{35}Cl^{35}Cl)-H]$^-$; HRMS (ESI) calcd for $C_{14}H_{12}Cl_3N_2O_7S_2$ [M(^{35}Cl^{35}Cl^{35}Cl)-H]$^-$ 488.9157, found 488.9152.

Sodium (3'-(sulfamoyloxy)-[1,1'-biphenyl]-3-yl)sulfamate, (54)

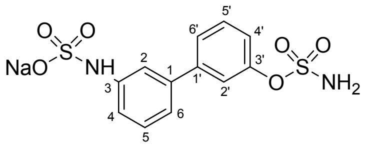

Compound **54** was synthesised according to general procedure G, using the following reagents: 2,2,2-trichloroethyl (3'-(sulfamoyloxy)-[1,1'-biphenyl]-3-yl)sulfamate (**50**) (200 mg, 0.42 mmol), MeOH (4.2 mL), acetate buffer pH 4.65 (4.2 mL) and zinc (275 mg, 4.20 mmol). The crude product was converted to the sodium salt by ion-exchange chromatography (DOWEX 50WX2 - Na$^+$ form). The crude product was purified by column chromatography (silica gel, EtOAc:MeOH, 1:0 → 93:7) to yield the *title compound* as a white solid (128 mg, 84%); R_f = 0.21 (EtOAc:MeOH, 93:7; KMnO$_4$); m.p. 185.5-187.5 °C; λ_{max} (EtOH)/nm 235.2; IR (neat) ν_{max}/cm^{-1} 3367, 3306, 3263, 1602, 1576, 1468, 1423, 1360, 1239, 1199, 1176, 1151, 1074, 1052; ^{1}H NMR (500 MHz, MeOD) δ 7.16 (2H, dd, *J* = 8.0, 2.1 Hz, 2 × ArH), 7.32 – 7.26 (2H, m, H-5

or H-5' and Ar*H*), 7.47 (1H, dd, *J* = 8.1, 8.1 Hz, H-5 or H-5'), 7.51 (1H, dd, *J* = 2.0 Hz, H-2 or H-2'), 7.60 – 7.55 (2H, m, 2 × Ar*H*); ^{13}C NMR (126 MHz, MeOD) δ 117.2 (C-2 or C-2'), 118.2 (CH Ar), 120.5 (CH Ar), 121.9 (CH Ar), 122.0 (CH Ar), 126.3 (CH Ar), 130.2 (C-5 or C-5'), 130.9 (C-5 or C-5'), 141.7 (C-3), 143.7 (C-1 or C-1'), 144.6 (C-1 or C-1'), 152.4 (C-3'); LRMS (ES$^-$) *m/z* 343.2 [M-Na]$^-$; HRMS (ESI) calcd for $C_{12}H_{11}N_2O_6S_2$ [M-Na]$^-$ 343.0064, found 343.0052.

Sodium (3'-(sulfamoyloxy)-[1,1'-biphenyl]-4-yl)sulfamate, (55)

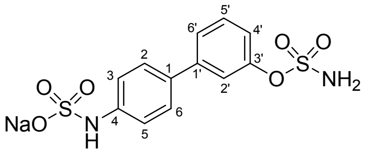

Compound **55** was synthesised according to general procedure G, using the following reagents: 2,2,2-trichloroethyl (3'-(sulfamoyloxy)-[1,1'-biphenyl]-4-yl)sulfamate (**51**) (100 mg, 0.21 mmol), MeOH (2.1 mL), acetate buffer pH 4.65 (2.1 mL) and zinc (137 mg, 2.10 mmol). The crude product was converted to the sodium salt by ion-exchange chromatography (DOWEX 50WX2 - Na$^+$ form). The crude product was purified by column chromatography (silica gel, EtOAc:MeOH, 1:0 → 93:7) to yield the *title compound* as a white solid (62 mg, 80%); R_f = 0.21 (EtOAc:MeOH, 93:7; $KMnO_4$); m.p. No clear m.p., decomposition range 185-195 °C; λ_{max} (EtOH)/nm 280.5; IR (neat) ν_{max}/cm^{-1} 3362, 3254, 1607, 1586, 1573, 1523, 1480, 1365, 1195, 1183, 1154, 1064; ^{1}H NMR (500 MHz, MeOD) δ 7.26 – 7.19 (1H, m, H-4' or H-6'), 7.26 (2H, d, *J* = 8.6 Hz, H-3, 5), 7.44 (1H, dd, *J* = 8.2, 8.2 Hz, H-5'), 7.57 – 7.48 (4H, m, 4 × Ar*H*); ^{13}C NMR (126 MHz, MeOD) δ 118.8 (C-3, 5), 121.2 (CH Ar), 121.2 (CH Ar), 125.6 (CH Ar), 128.2 (C-2, 6), 130.9 (C-5'), 133.4 (C-4), 143.2 (C-1 or C-1'), 144.3 (C-1 or C-1'), 152.5 (C-3'); LRMS (ES$^-$) *m/z* 343.2 [M-Na]$^-$; HRMS (ESI) calcd for $C_{12}H_{11}N_2O_6S_2$ [M-Na]$^-$ 343.0064, found 343.0052.

Sodium (3-(3-(sulfamoyloxy)phenoxy)phenyl)sulfamate, (56)

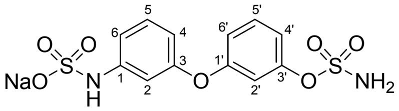

Compound **56** was synthesised according to general procedure G, using the following reagents: 2,2,2-trichloroethyl (3-(3-(sulfamoyloxy)phenoxy)phenyl)sulfamate (**52**) (150 mg, 0.31 mmol), MeOH (3.1 mL), acetate buffer pH 4.65 (3.1 mL) and zinc

(200 mg, 3.06 mmol). The crude product was converted to the sodium salt by ion-exchange chromatography (DOWEX 50WX2 - Na^+ form). The crude product was purified by column chromatography (silica gel, EtOAc:MeOH, 1:0 → 93:7) to yield the *title compound* as a white solid (87 mg, 75%); R_f = 0.23 (EtOAc:MeOH, 93:7; $KMnO_4$); m.p. 133.5-135.5 °C; λ_{max} (EtOH)/nm 279.2; IR (neat) ν_{max}/cm^{-1} 3472, 3346, 3093, 1592, 1478, 1368, 1185, 1150, 1045; ^{1}H NMR (500 MHz, MeOD) δ 6.55 (1H, dd, *J* = 8.4, 2.7 Hz, Ar*H*), 6.99 – 6.89 (4H, m, 4 × Ar*H*), 7.03 (1H, d, *J* = 7.7 Hz, Ar*H*), 7.20 (1H, dd, *J* = 8.0, 8.0 Hz, H-5 or H-5'), 7.35 (1H, dd, *J* = 8.1, 8.1 Hz, H-5 or H-5'); ^{13}C NMR (126 MHz, MeOD) δ 109.7 (CH Ar), 112.5 (CH Ar), 113.4 (CH Ar), 114.2 (CH Ar), 117.4 (CH Ar), 117.7 (CH Ar), 130.9 (C-5 or C-5'), 131.3 (C-5 or C-5'), 144.9 (C-1), 152.9 (C-3'), 158.1 (C-3 or C-1'), 160.0 (C-3 or C-1'); LRMS (ES$^-$) *m/z* 359.2 [M-Na]$^-$; HRMS (ESI) calcd for $C_{12}H_{11}N_2O_7S_2$ [M-Na]$^-$ 359.0013, found 359.0000.

Sodium (4-(3-(sulfamoyloxy)phenoxy)phenyl)sulfamate, (57)

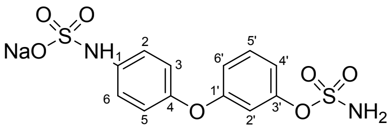

Compound **57** was synthesised according to general procedure G, using the following reagents: 2,2,2-trichloroethyl (4-(3-(sulfamoyloxy)phenoxy)phenyl)sulfamate (**53**) (150 mg, 0.31 mmol), MeOH (3.1 mL), acetate buffer pH 4.65 (3.1 mL) and zinc (200 mg, 3.06 mmol). The crude product was converted to the sodium salt by ion-exchange chromatography (DOWEX 50WX2 - Na^+ form). The crude product was purified by column chromatography (silica gel, EtOAc:MeOH, 1:0 → 93:7) to yield the *title compound* as a white solid (81 mg, 70%); R_f = 0.22 (EtOAc:MeOH, 93:7; $KMnO_4$); m.p. 180.0-182.0 °C; λ_{max} (EtOH)/nm 241.0; IR (neat) ν_{max}/cm^{-1} 3368, 3261, 3227, 3103, 1600, 1508, 1479, 1363, 1207, 1169, 1159, 1116, 1054; ^{1}H NMR (500 MHz, MeOD) δ 6.88 – 6.84 (2H, m, H-2' and H-4' or H-6'), 6.94 (2H, d, *J* = 8.9 Hz, H-3, 5), 6.99 (2H, ddd, *J* = 8.1, 2.0 and 1.0 Hz, H-4' or H-6'), 7.23 (2H, d, *J* = 8.9 Hz, H-2, 3), 7.33 (1H, dd, *J* = 8.6, 8.6 Hz, H-5'); ^{13}C NMR (126 MHz, MeOD) δ 112.6 (C-2'), 116.4 (C-4' or C-6'), 117.1 (C-4' or C-6'), 120.9 (C-2, 6), 121.4 (C-3, 5), 131.2 (C-5), 139.7 (C-1), 151.5 (C-4 or C-3'), 152.9 (C-4 or C-3'), 161.0 (C-1'); LRMS (ES$^-$) *m/z* 359.2 [M-Na]$^-$; HRMS (ESI) calcd for $C_{12}H_{11}N_2O_7S_2$ [M-Na]$^-$ 359.0013, found 358.9998.

2'-(bis((2,2,2-Trichloroethoxy)sulfonyl)amino)-[1,1'-biphenyl]-3-yl bis(2,4-dimethoxy benzyl)sulfamate, (58)

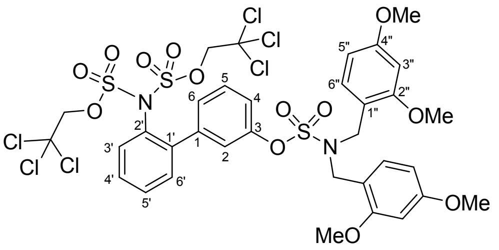

Compound (**58**) was synthesised according to general procedure H, using the following reagents: 2'-amino-[1,1'-biphenyl]-3-yl bis(2,4-dimethoxybenzyl)sulfamate (**5**) (550 mg, 0.97 mmol), 4-(dimethylamino)pyridine (262 mg, 2.14 mmol), triethylamine (543 µL, 394 mg, 3.90 mmol), 2,2,2-trichloroethyl chlorosulfate (**42**) (519 µL, 966 mg, 3.90 mmol) and THF (10 + 5 mL). The crude product was purified by column chromatography (silica gel, petrol:EtOAc, 1:0 → 3:1) to yield the *title compound* as a pale yellow oil (673 mg, 70%); R_f = 0.30 (petrol:EtOAC, 3:1); λ_{max} (EtOH)/nm 276.5; IR (neat) ν_{max}/cm^{-1} 1612, 1588, 1508, 1425, 1369, 1185, 1156, 1037, 999; ^{1}H NMR (500 MHz, $CDCl_3$) δ 3.71 (6H, s, 2 × ArOCH_3), 3.76 (6H, s, 2 × ArOCH_3), 4.45 (2H, d, *J* = 11.4 Hz, CH_2CCl$_3$), 4.49 (4H, s, 2 × ArCH_2), 4.66 (2H, d, *J* = 11.4 Hz, CH_2CCl$_3$), 6.37 (2H, d, *J* = 2.4 Hz, H-3”), 6.43 (2H, dd, *J* = 8.3, 2.4 Hz, H-5”), 7.18 (1H, dd, *J* = 1.4, 1.4 Hz, H-2), 7.24 (1H, ddd, *J* = 7.6, 2.6 and 2.6 Hz, H-4 or H-6), 7.29 (2H, d, *J* = 8.4 Hz, H-6”), 7.39 (1H, dd, *J* = 7.6, 1.7 Hz, H-3’), 7.45 – 7.40 (2H, m, H-5 and H-4 or H-6), 7.56 – 7.51 (1H, m, H-4’ or H-5’), 7.62 – 7.57 (1H, m, H-4’ or H-5’), 7.68 (1H, dd, *J* = 7.9, 1.3 Hz, H-6’); ^{13}C NMR (126 MHz, $CDCl_3$) δ 47.0 (Ar*C*H$_2$), 55.2 (ArO*C*H$_3$), 55.5 (ArO*C*H$_3$), 81.5 (*C*H$_2$CCl$_3$), 92.7 (CH$_2$*C*Cl$_3$), 98.3 (C-3”), 104.2 (C-5”), 116.6 (C-1”), 122.3 (C-4 or C-6), 123.4 (C-2), 127.4 (CH Ar), 129.6 (CH Ar), 129.7 (CH Ar), 131.1 (C-6”), 131.5 (C-4’ or C-5’), 132.5 (C-3’), 132.7 (C-2’), 139.5 (C-1 or C-1’), 141.9 (C-1 or C-1’), 150.7 (C-3), 158.6 (C-2” or C-4”), 160.7 (C-2” or C-4”); HRMS (ESI) calcd for $C_{34}H_{38}Cl_6N_3O_{13}S_3$ [M(6 × ^{35}Cl)+NH$_4$]$^+$ 1001.9692, found 1001.9675.

***O*,*O*-bis(2,2,2-Trichloroethyl) (2-(3-((*N*,*N*-bis(2,4-dimethoxybenzyl)sulfamoyl)oxy)phenoxy)phenyl)iminodisulfate, (59)**

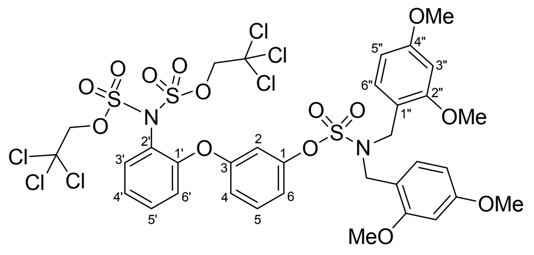

Compound (**59**) was synthesised according to general procedure H, using the following reagents: 2'-amino-[1,1'-biphenyl]-3-yl bis(2,4-dimethoxybenzyl)sulfamate (**22**) (450 mg, 0.77 mmol), 4-(dimethylamino)pyridine (208 mg, 1.70 mmol), triethylamine (432 µL, 314 mg, 3.01 mmol), 2,2,2-trichloroethyl chlorosulfate (**42**) (413 µL, 769 mg, 3.01 mmol) and THF (8 + 4 mL). The crude product was purified by column chromatography (silica gel, petrol:EtOAc, 1:0 → 8:2) to yield the *title compound* as a pale yellow oil (428 mg, 55%); R_f = 0.29 (petrol:EtOAC, 8:2); λ_{max} (EtOH)/nm 277.6; IR (neat) ν_{max}/cm^{-1} 1612, 1588, 1508, 1479, 1429, 1371, 1261, 1208, 1191, 1158, 1117, 1038, 1003; ^{1}H NMR (500 MHz, $CDCl_3$) δ 3.71 (6H, s, 2 × ArOCH_3), 3.78 (6H, s, 2 × ArOCH_3), 4.46 (4H, s, 2 × ArCH_2), 4.96 (2H, d, *J* = 11.3 Hz, CH_2CCl$_3$), 5.04 (2H, d, *J* = 11.3 Hz, CH_2CCl$_3$), 6.38 (2H, d, *J* = 2.4 Hz, H-3”), 6.42 (2H, dd, *J* = 8.4, 2.4 Hz, H-5”), 6.90 (1H, dd, *J* = 8.4, 1.3 Hz, H-6’), 6.94 (1H, dd, *J* = 2.3, 2.3 Hz, H-2), 7.01 (2H, dd, *J* = 8.3, 2.3 Hz, H-4 and H-6), 7.21 (1H, ddd, *J* = 7.8, 7.8 and 1.3 Hz, H-4’), 7.25 (2H, d, *J* = 8.4 Hz, H-6”), 7.33 (1H, dd, *J* = 8.2, 8.2 Hz, H-5), 7.43 (1H, ddd, *J* = 8.5, 7.6 and 1.7 Hz, H-5’), 7.58 (1H, dd, *J* = 8.0, 1.6 Hz, H-3’); ^{13}C NMR (126 MHz, $CDCl_3$) δ 47.1 (Ar*C*H$_2$), 55.2 (ArO*C*H$_3$), 55.5 (ArO*C*H$_3$), 81.6 (*C*H$_2$CCl$_3$), 92.7 (CH$_2$*C*Cl$_3$), 98.3 (C-3”), 104.1 (C-5”), 114.4 (C-2), 116.4 (C-1”), 118.1 (C-4 or C-6), 118.3 (C-4 or C-6), 118.9 (C-6’), 124.1 (C-4’), 124.6 (C-2’), 130.9 (C-5), 131.0 (C-6”), 131.8 (C-5’), 133.0 (C-3’), 151.7 (C-1), 155.1 (C-3 or C-1’), 155.7 (C-3 or C-1’), 158.5 (C-2” or C-4”), 160.8 (C-2” or C-4”); HRMS (ESI) calcd for $C_{34}H_{38}Cl_6N_3O_{14}S_3$ [M(6 × ^{35}Cl)+NH$_4$]$^+$ 1017.9642, found 1017.9658.

2,2,2-Trichloroethyl (3'-(sulfamoyloxy)-[1,1'-biphenyl]-2-yl)((2,2,2-trichloroethoxy) sulfonyl)sulfamate, (60)

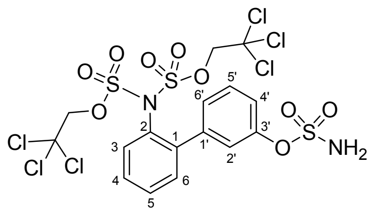

Compound **60** was synthesised according to general procedure B, using the following reagents: 2'-(bis((2,2,2-trichloroethoxy)sulfonyl)amino)-[1,1'-biphenyl]-3-yl bis(2,4-dimethoxybenzyl)sulfamate (**58**) (550 mg, 0.56 mmol), DCM (5.1 mL) and TFA (0.6 mL). The crude product was purified by column chromatography (silica gel, petrol:EtOAc, 1:0 → 8:2) to yield the *title compound* as a pale orange oil (344 mg, 90%); R_f = 0.29 (petrol:EtOAc, 8:2); λ_{max} (EtOH)/nm 232.0; IR (neat) ν_{max}/cm^{-1} 3456, 3346, 1545, 1473, 1424, 1388, 1189, 1152, 1093, 990; ^{1}H NMR (500 MHz, $CDCl_3$) δ 4.53 (2H, d, *J* = 11.4 Hz, C*H*$_2$CCl$_3$), 4.72 (2H, d, *J* = 11.4 Hz, C*H*$_2$CCl$_3$), 5.10 (2H, s, NH$_2$), 7.45 – 7.39 (2H, m, 2 × Ar*H*), 7.47 (1H, dd, *J* = 7.6, 1.7 Hz, H-3'), 7.59 – 7.52 (3H, m, 3 × Ar*H*), 7.65 – 7.60 (1H, m, H-4' or H-5'), 7.69 – 7.66 (1H, m, H-6'); ^{13}C NMR (126 MHz, $CDCl_3$) δ 81.6 (*C*H$_2$CCl$_3$), 92.6 (CH$_2$*C*Cl$_3$), 122.6 (CH Ar), 122.8 (CH Ar), 128.6 (CH Ar), 129.7 (C-6'), 130.0 (CH Ar), 130.5 (CH Ar), 131.8 (C-4' or C-5'), 132.3 (C-3'), 132.6 (C-2'), 139.7 (C-1 or C-1'), 141.6 (C-1 or C-1'), 150.1 (C-3); HRMS (ESI) calcd for $C_{16}H_{18}Cl_6N_3O_9S_3$ [M(6 × ^{35}Cl)+NH$_4$]$^+$ 701.8331, found 701.8334.

***O*,*O*-bis(2,2,2-Trichloroethyl) (2-(3-(sulfamoyloxy)phenoxy)phenyl) iminodisulfate, (61)**

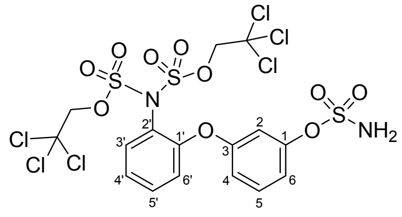

Compound **61** was synthesised according to general procedure B, using the following reagents: *O*,*O*-bis(2,2,2-trichloroethyl) (2-(3-((*N*,*N*-bis(2,4-dimethoxybenzyl) sulfamoyl)oxy)phenoxy)phenyl)iminodisulfate (**59**) (350 mg, 0.35 mmol), DCM (3.2 mL) and TFA (0.4 mL). The crude product was purified by column chromatography (silica gel, petrol:EtOAc, 1:0 → 3:1) to yield the *title compound* as a

clear brown oil (240 mg, 86%); $R_f = 0.29$ (petrol:EtOAc, 3:1); λ_{max} (EtOH)/nm 277.2; IR (neat) ν_{max}/cm^{-1} 3398, 3295, 1590, 1490, 1479, 1428, 1375, 1259, 1186, 1118, 1083, 1044, 1002; ^{1}H NMR (500 MHz, DMSO-d_6) δ 5.28 (2H, d, J = 11.3 Hz, CH_2CCl$_3$), 5.36 (2H, d, J = 11.3 Hz, CH_2CCl$_3$), 7.10 – 7.04 (3H, m, H-2 and H-4 or H-6 and H-6'), 7.17 (1H, ddd, J = 8.3, 2.1 and 0.9 Hz, H-4 or H-6), 7.32 (1H, ddd, J = 7.9, 7.9 and 1.3 Hz, H-4'), 7.61 – 7.51 (2H, m, H-5 and H-5'), 7.88 (1H, dd, J = 8.0, 1.6 Hz, H-3'), 8.10 (2H, s, ArOSO$_2$NH_2); ^{13}C NMR (126 MHz, DMSO-d_6) δ 80.7 (CH$_2$CCl$_3$), 92.8 (CH$_2$$CCl_3$), 114.1 (C-2), 117.8 (C-4 or C-6 or C-6'), 118.4 (C-4 or C-6 or C-6'), 118.9 (C-4 or C-6), 124.0 (C-4'), 124.4 (C-2'), 131.2 (C-5), 132.2 (C-3'), 133.4 (C-5'), 151.1 (C-1), 154.4 (C-3 or C-1'), 155.4 (C-3 or C-1'); HRMS (ESI) calcd for $C_{16}H_{13}Cl_6N_2O_{10}S_3$ [M(6 × ^{35}Cl)-H]$^-$ 698.7869, found 698.7868.

Sodium (3'-(sulfamoyloxy)-[1,1'-biphenyl]-2-yl)sulfamate, (62)

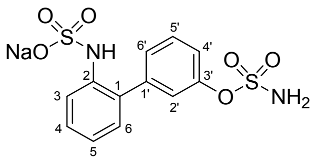

To 2,2,2-trichloroethyl (3'-(sulfamoyloxy)-[1,1'-biphenyl]-2-yl)((2,2,2-trichloroethoxy) sulfonyl)sulfamate (**60**) (110 mg, 0.16 mmol) in MeOH (3.5 mL) were added 3.5 mL of acetate buffer pH 4.65 and zinc (210 mg, 3.20 mmol). The resulting reaction mixture was stirred at RT for 24 h, until LCMS indicated completion of the deprotection. Upon completion, the heterogeneous mixture was filtered through Celite and washed with MeOH (10 mL). The filtrate was concentrated *in vacuo* to give a crude oil, which was dissolved in a mixture of MeOH (3.5 mL) and acetate buffer pH 4.65 (3.5 mL). Acetic acid was added to acidify the reaction mixture to pH 4.0. The resulting solution was heated at 40 °C for 12 h. Upon completion, the solvents were removed *in vacuo* and the crude product was converted to the sodium salt by ion-exchange chromatography (DOWEX 50WX2 - Na$^+$ form). The crude product was purified by column chromatography (silica gel, EtOAc:MeOH, 1:0 → 93:7) to yield the *title compound* as a white solid (30 mg, 50%); $R_f = 0.25$ (EtOAc:MeOH, 93:7; KMnO$_4$); m.p. 142.0-144.0 °C; λ_{max} (EtOH)/nm 275.6; IR (neat) ν_{max}/cm^{-1} 3501, 3305, 3205, 3071, 2922, 1670, 1609, 1580, 1475, 1370, 1183, 1149, 1039; ^{1}H NMR (500 MHz, MeOD) δ 7.03 (1H, ddd, J = 7.4, 7.4 and 1.2 Hz, H-4), 7.18 (1H, dd, J = 7.7, 1.7 Hz, H-3), 7.30 (1H, ddd, J = 8.5, 7.9 and 1.6 Hz, H-5), 7.35 (1H, ddd, J = 8.2, 2.3 and 1.1

Hz, H-4' or H-6'), 7.42 (1H, ddd, J = 7.4, 1.3 and 1.3 Hz, H-4' or H-6'), 7.46 (1H, dd, J = 1.9, 1.9 Hz, H-2'), 7.54 (1H, dd, J = 7.9, 7.9 Hz, H-5'), 7.72 (1H, dd, J = 8.4, 1.2 Hz, H-6); ^{13}C NMR (126 MHz, MeOD) δ 120.4 (C-6), 122.9 (C-4' or C-6'), 123.0 (C-4), 124.5 (C-2'), 128.8 (C-4' or C-6'), 129.6 (C-5), 131.0 (C-3), 131.3 (C-5'), 131.3 (C-1), 139.4 (C-2 or C-1'), 142.0 (C-2 or C-1'), 152.3 (C-3'); LRMS (ES$^-$) m/z 343.2 [M-Na]$^-$; HRMS (ESI) calcd for $C_{12}H_{11}N_2O_6S_2$ [M-Na]$^-$ 343.0064, found 343.0055.

Sodium (2-(3-(sulfamoyloxy)phenoxy)phenyl)sulfamate, (63)

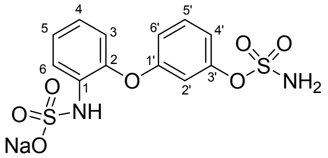

To O,O-bis(2,2,2-trichloroethyl) (2-(3-(sulfamoyloxy)phenoxy)phenyl)iminodisulfate (**63**) (140 mg, 0.20 mmol) in MeOH (3.5 mL) were added 3.5 mL of acetate buffer pH 4.65 and zinc (260 mg, 3.98 mmol). The resulting reaction mixture was stirred at RT for 24 h, until LCMS indicated completion of the deprotection. Upon completion, the heterogeneous mixture was filtered through Celite and washed with MeOH (10 mL). The filtrate was concentrated *in vacuo* to give a crude oil, which was dissolved in a mixture of MeOH (3.5 mL) and acetate buffer pH 4.65 (3.5 mL). Acetic acid was added to acidify the reaction mixture to pH 4.0. The resulting solution was heated at 40 °C for 12 h. Upon completion, the solvents were removed *in vacuo* and the crude product was converted to the sodium salt by ion-exchange chromatography (DOWEX 50WX2 - Na^+ form). The crude product was purified by column chromatography (silica gel, EtOAc:MeOH, 1:0 → 93:7) to yield the *title compound* as an off-white solid (25 mg, 33%); R_f = 0.24 (EtOAc:MeOH, 93:7; $KMnO_4$); m.p. No clear m.p., decomposition range 160-170 °C; λ_{max} (EtOH)/nm 270.6, 276.2; IR (neat) ν_{max}/cm^{-1} 3409, 3248, 2920, 1611, 1593, 1499, 1479, 1380, 1226, 1195, 1118, 1059; ^{1}H NMR (500 MHz, MeOD) δ 6.93 – 6.91 (2H, m, H-3 and H-4), 6.95 – 6.93 (1H, m, H-4' or H-6'), 6.96 (1H, dd, J = 2.3, 2.3 Hz, H-2'), 7.04 (1H, ddd, J = 8.2, 2.2 and 0.9 Hz, H-4' or H-6'), 7.13 (1H, ddd, J = 8.5, 6.2 and 2.6 Hz, H-5), 7.36 (1H, dd, J = 8.2, 8.2 Hz, H-5'), 7.73 – 7.69 (1H, m, H-6); ^{13}C NMR (126 MHz, MeOD) δ 112.9 (C-2'), 116.7 (C-4' or C-6'), 117.9 (C-4' or C-6'), 120.0 (C-6), 120.7 (C-3), 122.8 (C-4), 126.0 (C-5), 131.4 (C-5'), 134.9 (C-1), 145.2 (C-2), 152.9 (C-3'), 159.8 (C-1'); LRMS (ES$^-$) m/z 359.2 [M-Na]$^-$; HRMS (ESI) calcd for $C_{12}H_{11}N_2O_7S_2$ [M-Na]$^-$ 359.0013, found 358.9998.

3-(2-Cyanophenoxy)phenyl bis(2,4-dimethoxybenzyl)sulfamate, (67)

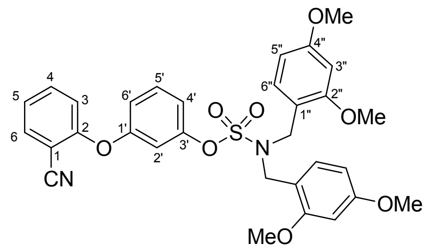

Compound **67** was synthesised according to general procedure C, using the following reagents: 3-hydroxyphenyl bis(2,4-dimethoxybenzyl)sulfamate (**18**) (1.0 g, 2.04 mmol), potassium carbonate (339 mg, 2.45 mmol), 2-fluorobenzonitrile (495 mg, 443 μL, 4.08 mmol) and *N*,*N*-dimethylformamide (20 mL). The crude orange oil was purified by column chromatography (silica gel, petrol:EtOAc, 1:0 → 7:3) to yield the *title compound* as a pale yellow oil (975 mg, 81%); $R_f = 0.31$ (petrol:EtOAc, 7:3); λ_{max} (EtOH)/nm 278.2, 283.0; IR (neat) ν_{max}/cm^{-1} 2230, 1611, 1589, 1507, 1478, 1449, 1371, 1253, 1208, 1173, 1157, 1034; ^{1}H NMR (500 MHz, $CDCl_3$) δ 3.73 (6H, s, 2 × ArOC*H*$_3$), 3.78 (6H, s, 2 × ArOC*H*$_3$), 4.44 (4H, s, 2 × ArC*H*$_2$), 6.39 (2H, d, *J* = 2.4 Hz, H-3”), 6.42 (2H, dd, *J* = 8.3, 2.4 Hz, H-5”), 6.64 (1H, dd, *J* = 2.3, 2.3 Hz, H-2), 6.84 (1H, dd, *J* = 8.5, 1.0 Hz, H-6’), 6.95 (1H, ddd, *J* = 8.2, 2.4 and 0.9 Hz, H-4 or H-6), 7.02 (1H, ddd, *J* = 8.3, 2.2 and 0.9 Hz, H-4 or H-6), 7.16 (1H, ddd, *J* = 7.6, 7.6 and 1.0 Hz, H-4’), 7.25 (2H, d, *J* = 8.5 Hz, H-6”), 7.33 (1H, dd, *J* = 8.3, 8.3 Hz, H-5), 7.48 (1H, ddd, *J* = 8.6, 7.6 and 1.7 Hz, H-5’), 7.68 (1H, dd, *J* = 7.7, 1.7 Hz, H-3’); ^{13}C NMR (126 MHz, $CDCl_3$) δ 47.2 (Ar*C*H$_2$), 55.3 (ArO*C*H$_3$), 55.5 (ArO*C*H$_3$), 98.3 (C-3”), 104.0 (C-5”), 104.1 (C-5”), 114.2 (C-2), 115.9 (Ar*C*N), 116.5 (C-1”), 117.1 (C-6’), 118.0 (C-4 or C-6), 118.8 (C-4 or C-6), 123.3 (C-4’), 130.7 (C-5), 131.1 (C-6”), 134.1 (C-3’), 134.4 (C-5’), 151.5 (C-1), 155.6 (C-3), 158.6 (C-2” or C-4”), 159.3 (C-1’), 160.8 (C-2” or C-4”); HRMS (ESI) calcd for $C_{31}H_{34}N_3O_8S$ $[M+NH_4]^+$ 608.2061, found 608.2056.

3-(3-Cyanophenoxy)phenyl bis(2,4-dimethoxybenzyl)sulfamate, (68)

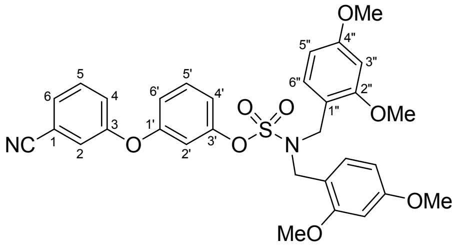

Compound **68** was synthesised according to general procedure C, using the following reagents: 3-hydroxyphenyl bis(2,4-dimethoxybenzyl)sulfamate (**18**) (1.0 g, 2.04 mmol), potassium carbonate (339 mg, 2.45 mmol), 3-fluorobenzonitrile (495 mg, 437 μL, 4.08 mmol) and *N*,*N*-dimethylformamide (20 mL). The crude orange oil was purified by column chromatography (silica gel, petrol:EtOAc, 1:0 → 7:3) to yield the *title compound* as a yellow oil (740 mg, 61%); R_f = 0.30 (petrol:EtOAc, 7:3); λ_{max} (EtOH)/nm 277.8; IR (neat) ν_{max}/cm^{-1} 2232, 1611, 1588, 1507, 1478, 1370, 1255, 1207, 1175, 1157, 1111, 1033; ^{1}H NMR (500 MHz, $CDCl_3$) δ 3.72 (6H, s, 2 × ArOC*H*$_3$), 3.78 (6H, s, 2 × ArOC*H*$_3$), 4.45 (4H, s, 2 × ArC*H*$_2$), 6.38 (2H, d, *J* = 2.4 Hz, H-3"), 6.42 (2H, dd, *J* = 8.3, 2.4 Hz, H-5"), 6.72 (1H, dd, *J* = 2.3, 2.3 Hz, H-2), 6.88 (1H, ddd, *J* = 8.3, 2.3 and 0.9 Hz, H-4 or H-6), 6.96 (1H, ddd, *J* = 8.2, 2.2 and 0.9 Hz, H-4 or H-6), 7.20 (1H, ddd, *J* = 8.0, 2.5 and 1.4 Hz, H-6'), 7.26 – 7.22 (3H, m, H-2' and H-6"), 7.31 (1H, dd, *J* = 8.2, 8.2 Hz, H-5), 7.41 – 7.38 (1H, m, H-4'), 7.45 – 7.41 (1H, m, H-5'); ^{13}C NMR (126 MHz, $CDCl_3$) δ 47.1 (Ar*C*H$_2$), 55.2 (ArO*C*H$_3$), 55.5 (ArO*C*H$_3$), 98.3 (C-3"), 104.1 (C-5"), 113.7 (C-2), 113.9 (C-3'), 116.5 (C-1"), 117.4 (C-4 or C-6), 118.1 (C-4 or C-6), 118.2 (Ar*C*N), 121.7 (C-2'), 123.1 (C-6'), 127.1 (C-4'), 130.7 (C-5 or C-5'), 130.9 (C-5 or C-5'), 131.1 (C-6"), 151.6 (C-1), 156.4 (C-3 or C-1'), 157.5 (C-3 or C-1'), 158.6 (C-2" or C-4"), 160.8 (C-2" or C-4"); HRMS (ESI) calcd for $C_{31}H_{34}N_3O_8S$ $[M+NH_4]^+$ 608.2061, found 608.2058.

3-(4-Cyanophenoxy)phenyl bis(2,4-dimethoxybenzyl)sulfamate, (69)

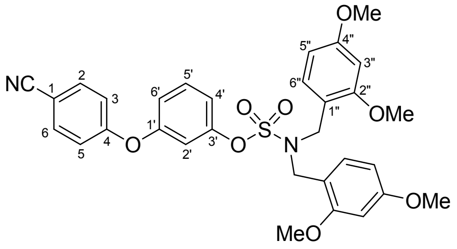

Compound **69** was synthesised according to general procedure C, using the following reagents: 3-hydroxyphenyl bis(2,4-dimethoxybenzyl)sulfamate (**18**) (1.0 g, 2.04 mmol), potassium carbonate (339 mg, 2.45 mmol), 4-fluorobenzonitrile (495 mg, 4.08 mmol) and *N*,*N*-dimethylformamide (20 mL). The crude orange oil was purified by column chromatography (silica gel, petrol:EtOAc, 1:0 → 7:3) to yield the *title compound* as a pale yellow oil (870 mg, 72%); R_f = 0.31 (petrol:EtOAc, 7:3); λ_{max} (EtOH)/nm 272.0; IR (neat) ν_{max}/cm^{-1} 2226, 1611, 1589, 1504, 1478, 1370, 1250, 1208, 1157, 1112, 1033; ^{1}H NMR (500 MHz, $CDCl_3$) δ 3.72 (6H, s, 2 × ArOCH_3), 3.78 (6H, s, 2 × ArOCH_3), 4.44 (4H, s, 2 × ArCH_2), 6.37 (2H, d, *J* = 2.4 Hz, H-3”), 6.42 (2H, dd, *J* = 8.4, 2.4 Hz, H-5”), 6.72 (1H, dd, *J* = 2.3, 2.3 Hz, H-2), 6.93 (1H, ddd, *J* = 8.2, 2.3 and 0.9 Hz, H-4 or H-6), 7.02 – 6.97 (3H, m, H-4 or H-6 and H-2’, 6’), 7.24 (2H, d, *J* = 8.4 Hz, H-6”), 7.34 (1H, dd, *J* = 8.2, 8.2 Hz, H-5), 7.61 (2H, d, *J* = 8.8 Hz, H-3’, 5’); ^{13}C NMR (126 MHz, $CDCl_3$) δ 47.1 (Ar*C*H_2), 55.2 (ArO*C*H_3), 55.5 (ArO*C*H_3), 98.3 (C-3”), 104.1 (C-5”), 106.5 (C-4’), 114.4 (C-2), 116.4 (C-1”), 118.2 (C-4 or C-6), 118.3 (C-2’, 6’), 118.8 (C-4 or C-6), 118.8 (Ar*C*N), 130.8 (C-5), 131.1 (C-6”), 134.3 (C-3’, 5’), 151.6 (C-1), 155.6 (C-3), 158.6 (C-2” or C-4”), 160.8 (C-2” or C-4”), 161.0 (C-1’); HRMS (ESI) calcd for $C_{31}H_{31}N_2O_8S$ $[M+H]^+$ 591.1796, found 591.1794.

2-(3-((*N*,*N*-bis(2,4-Dimethoxybenzyl)sulfamoyl)oxy)phenoxy)benzoic acid, (70)

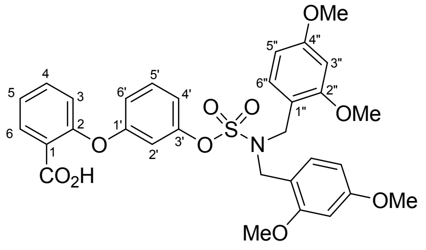

To 3-(2-cyanophenoxy)phenyl bis(2,4-dimethoxybenzyl)sulfamate (**67**) (600 mg, 1.02 mmol) in dioxane (5.1 mL) was added 2 M aq. solution of NaOH (5.1 mL, 10.2 mmol). The resulting mixture was heated at 130 °C for 2 h under microwave irradiation. After cooling, the mixture was acidified to pH 1-2 with using a 4 M aq. solution of HCl and stirred at RT for 30 min. The reaction was then diluted with water (20 mL) and extracted with EtOAc (3 × 25 mL). The pooled organic extracts were washed with water (30 mL) and brine (30 mL), dried over $MgSO_4$ and concentrated *in vacuo*. The crude product was purified by column chromatography (silica gel,

petrol:EtOAc:AcOH, 1:0:0 → 50:49.7:0.3) to yield the *title compound* as a yellow oil (279 mg, 45%); R_f = 0.30 (petrol:EtOAc:AcOH, 50:49.7:0.3); λ_{max} (EtOH)/nm 278.0; IR (neat) ν_{max}/cm^{-1} 1697, 1605, 1589, 1507, 1477, 1455, 1370, 1249, 1207, 1157, 1112, 1034; ^{1}H NMR (500 MHz, $CDCl_3$) δ 3.72 (6H, s, 2 × ArOC*H*$_3$), 3.77 (6H, s, 2 × ArOC*H*$_3$), 4.43 (4H, s, 2 × ArC*H*$_2$), 6.37 (2H, d, *J* = 2.4 Hz, H-3"), 6.41 (2H, dd, *J* = 8.4, 2.4 Hz, H-5"), 6.67 (1H, dd, *J* = 2.3, 2.3 Hz, H-2'), 6.92 – 6.86 (2H, m, H-3 and H-4' or H-6'), 7.00 (1H, ddd, *J* = 8.3, 2.3 and 0.9 Hz, H-4' or H-6'), 7.26 – 7.22 (m, 3H, H-5 and H-6"), 7.31 (dd, *J* = 8.2, 8.2 Hz, 1H, H-5'), 7.50 (1H, ddd, *J* = 8.3, 7.3 and 1.8 Hz, H-4), 8.15 (1H, dd, *J* = 7.9, 1.8 Hz, H-6); ^{13}C NMR (126 MHz, $CDCl_3$) δ 47.2 (Ar*C*H$_2$), 55.3 (ArO*C*H$_3$), 55.5 (ArO*C*H$_3$), 98.3 (C-3"), 104.2 (C-5"), 113.7 (C-2'), 116.5 (C-1"), 117.3 (C-4' or C-6'), 118.4 (C-4' or C-6'), 119.1 (C-3), 120.6 (C-1), 124.2 (C-5), 130.6 (C-5'), 131.1 (C-6"), 133.5 (C-6), 135.0 (C-4), 151.6 (C-1), 156.2 (C-2 or C-1'), 156.6 (C-2 or C-1'), 158.5 (C-2" or C-4"), 160.8 (C-2" or C-4"), 167.1 (Ar*C*O$_2$H); LRMS (ES$^-$) *m/z* 608.5 [M-H]$^-$; HRMS (ESI) calcd for $C_{31}H_{30}NO_{10}S$ [M-H]$^-$ 608.1596, found 608.1578.

3-(3-((*N*,*N*-bis(2,4-Dimethoxybenzyl)sulfamoyl)oxy)phenoxy)benzoic acid, (71)

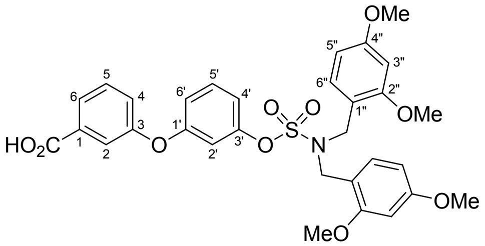

To 3-(3-cyanophenoxy)phenyl bis(2,4-dimethoxybenzyl)sulfamate (**68**) (600 mg, 1.02 mmol) in dioxane (5.1 mL) was added 2 M aq. solution of NaOH (5.1 mL, 10.2 mmol). The resulting mixture was heated at 130 °C for 2 h under microwave irradiation. After cooling, the mixture was acidified to pH 1-2 with using a 4 M aq. solution of HCl and stirred at RT for 30 min. The reaction was then diluted with water (20 mL) and extracted with EtOAc (3 × 25 mL). The pooled organic extracts were washed with water (30 mL) and brine (30 mL), dried over $MgSO_4$ and concentrated *in vacuo*. The crude product was purified by column chromatography (silica gel, petrol:EtOAc:AcOH, 1:0:0 → 50:49.7:0.3) to yield the *title compound* as a clear oil (495 mg, 80%); R_f = 0.31 (petrol:EtOAc:AcOH, 50:49.7:0.3); λ_{max} (EtOH)/nm 277.8; IR (neat) ν_{max}/cm^{-1} 1694, 1612, 1585, 1507, 1479, 1451, 1371, 1249, 1207, 1157, 1114,

1035; ^{1}H NMR (500 MHz, $CDCl_3$) δ 3.71 (6H, s, 2 × ArOCH_3), 3.77 (6H, s, 2 × ArOCH_3), 4.43 (4H, s, 2 × ArCH_2), 6.36 (2H, d, *J* = 2.4 Hz, H-3”), 6.40 (2H, dd, *J* = 8.4, 2.4 Hz, H-5”), 6.69 (1H, dd, *J* = 2.3, 2.3 Hz, H-2’), 6.88 (1H, ddd, *J* = 8.2, 2.4 and 1.0 Hz, H-4’ or H-6’), 6.93 (1H, ddd, *J* = 8.3, 2.2 and 0.9 Hz, H-4’ or H-6’), 7.25 – 7.20 (3H, m, H-4 and H-6”), 7.29 (1H, dd, *J* = 8.3, 8.3 Hz, H-5), 7.45 (1H, dd, *J* = 8.0, 8.0 Hz, H-5), 7.70 (1H, dd, *J* = 2.5, 1.5 Hz, H-2), 7.86 (1H, ddd, *J* = 7.8, 1.3 and 1.3 Hz, H-6); ^{13}C NMR (126 MHz, $CDCl_3$) δ 47.1 (Ar*C*H_2), 55.2 (ArO*C*H_3), 55.5 (ArO*C*H_3), 98.3 (C-3”), 104.1 (C-5”), 113.2 (C-2’), 116.6 (C-1”), 117.0 (C-4’ or C-6’), 117.5 (C-4’ or C-6’), 120.3 (C-2), 124.3 (C-4), 125.4 (C-6), 130.2 (C-5), 130.5 (C-5’), 131.1 (C-6”), 131.3 (C-1), 151.6 (C-3’), 157.1 (C-3 or C-1’), 157.4 (C-3 or C-1’), 158.6 (C-2” or C-4”), 160.7 (C-2” or C-4”), 170.9 (Ar*C*O_2H); LRMS (ES^-) *m/z* 608.5 $[M-H]^-$; HRMS (ESI) calcd for $C_{31}H_{30}NO_{10}S$ $[M-H]^-$ 608.1596, found 608.1577.

4-(3-((*N*,*N*-bis(2,4-Dimethoxybenzyl)sulfamoyl)oxy)phenoxy)benzoic acid, (72)

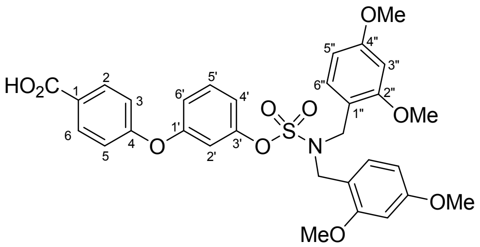

To 3-(4-cyanophenoxy)phenyl bis(2,4-dimethoxybenzyl)sulfamate (**69**) (600 mg, 1.02 mmol) in dioxane (5.1 mL) was added 2 M aq. solution of NaOH (5.1 mL, 10.2 mmol). The resulting mixture was heated at 130 °C for 2 h under microwave irradiation. After cooling, the mixture was acidified to pH 1-2 with using a 4 M aq. solution of HCl and stirred at RT for 30 min. The reaction was then diluted with water (20 mL) and extracted with EtOAc (3 × 25 mL). The pooled organic extracts were washed with water (30 mL) and brine (30 mL), dried over $MgSO_4$ and concentrated *in vacuo*. The crude product was purified by column chromatography (silica gel, petrol:EtOAc:AcOH, 1:0:0 → 50:49.7:0.3) to yield the *title compound* as a clear oil (403 mg, 65%); R_f = 0.32 (petrol:EtOAc:AcOH, 50:49.7:0.3); λ_{max} (EtOH)/nm 253.0; IR (neat) ν_{max}/cm^{-1} 1686, 1611, 1589, 1507, 1371, 1249, 1208, 1158, 1111, 1035; ^{1}H NMR (500 MHz, $CDCl_3$) δ (500 MHz, $CDCl_3$) 3.71 (6H, s, 2 × ArOCH_3), 3.77 (6H, s, 2 × ArOCH_3), 4.44 (4H, s, 2 × ArCH_2), 6.36 (2H, d, *J* = 2.4 Hz, H-3”), 6.41 (2H, dd, *J* = 8.4, 2.4 Hz, H-5”), 6.70 (1H, dd, *J* = 2.3, 2.3 Hz, H-2’), 6.95 (1H, ddd, *J* = 8.2, 2.3

and 0.9 Hz, H-4' or H-6'), 7.03 – 6.98 (3H, m, H-3, 5 and H-4' or H-6'), 7.25 (2H, d, *J* = 8.3 Hz, H-6"), 7.33 (1H, dd, *J* = 8.2, 8.2 Hz, H-5'), 8.09 (2H, d, *J* = 8.8 Hz, H-2, 6); ^{13}C NMR (126 MHz, $CDCl_3$) δ 47.1 (Ar*C*H_2), 55.2 (ArO*C*H_3), 55.5 (ArO*C*H_3), 98.3 (C-3"), 104.1 (C-5"), 114.3 (C-2'), 116.5 (C-1"), 117.5 (C-3, 5), 118.2 (C-4' or C-6'), 118.5 (C-4' or C-6'), 124.0 (C-1), 130.7 (C-5'), 131.1 (C-6"), 132.6 (C-2, 6), 151.6 (C-3'), 156.1 (C-1'), 158.6 (C-2" or C-4"), 160.8 (C-2" or C-4"), 162.1 (C-4), 171.2 (Ar*C*O_2H); LRMS (ES^-) *m/z* 608.4 $[M-H]^-$; HRMS (ESI) calcd for $C_{31}H_{30}NO_{10}S$ $[M-H]^-$ 608.1596, found 608.1575.

2-(3-(Sulfamoyloxy)phenoxy)benzoic acid, (73)

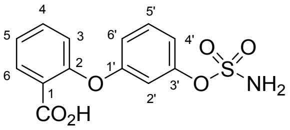

Compound **73** was synthesised according to general procedure B, using the following reagents: 2-(3-((*N*,*N*-bis(2,4-dimethoxybenzyl)sulfamoyl)oxy)phenoxy)benzoic acid (**70**) (200 mg, 0.33 mmol), DCM (3.0 mL) and TFA (0.3 mL). The crude product was purified by column chromatography (silica gel, petrol:EtOAc:AcOH, 1:0:0 → 50:49.7:0.3) to yield the *title compound* as an off-white solid (91 mg, 90%); R_f = 0.30 (petrol:EtOAc:AcOH, 50:49.7:0.3); m.p. 101.5-103.5 °C; λ_{max} (EtOH)/nm 278.6; IR (neat) ν_{max}/cm^{-1} 3405, 3298, 1681, 1602, 1573, 1478, 1448, 1385, 1263, 1252, 1190, 1112; ^{1}H NMR (500 MHz, DMSO-d_6) δ 6.82 (1H, dd, *J* = 2.4, 2.4 Hz, H-2'), 6.84 (1H, ddd, *J* = 8.3, 2.4 and 0.8 Hz, H-4' or H-6'), 7.00 (1H, ddd, *J* = 8.3, 2.2 and 0.9 Hz, H-4' or H-6'), 7.11 (1H, dd, *J* = 8.3, 1.1 Hz, H-3), 7.32 (1H, ddd, *J* = 7.6, 7.6 and 1.1 Hz, H-5), 7.41 (dd, *J* = 8.2, 8.2 Hz, 1H, H-5'), 7.60 (1H, ddd, *J* = 8.2, 7.3 and 1.8 Hz, H-4), 7.86 (1H, dd, *J* = 7.8, 1.8 Hz, H-6), 8.04 (2H, s, ArOSO$_2$N*H*$_2$), 12.85 (1H, s, ArCO$_2$*H*); ^{13}C NMR (126 MHz, DMSO-d_6) δ 111.4 (C-2'), 115.3 (C-4' or C-6'), 116.3 (C-4' or C-6'), 121.5 (C-3), 124.6 (C-1), 124.7 (C-5), 130.6 (C-5'), 131.5 (C-6), 133.7 (C-4), 151.0 (C-3'), 154.1 (C-2), 158.4 (C-1'), 166.3 (Ar*C*O_2H); LRMS (ES^-) *m/z* 308.2 $[M-H]^-$; HRMS (ESI) calcd for $C_{13}H_{10}NO_6S$ $[M-H]^-$ 308.0234, found 308.0222.

3-(3-(Sulfamoyloxy)phenoxy)benzoic acid, (74)

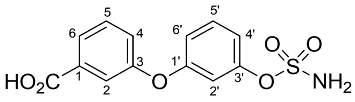

Compound **74** was synthesised according to general procedure B, using the following reagents: 3-(3-((*N*,*N*-bis(2,4-dimethoxybenzyl)sulfamoyl)oxy)phenoxy)benzoic acid (**71**) (200 mg, 0.33 mmol), DCM (3.0 mL) and TFA (0.3 mL). The crude product was purified by column chromatography (silica gel, petrol:EtOAc:AcOH, 1:0:0 → 50:49.7:0.3) to yield the *title compound* as an off-white solid (95 mg, 94%); R_f = 0.32 (petrol:EtOAc:AcOH, 50:49.7:0.3); m.p. 132.0-134.0 °C; λ_{max} (EtOH)/nm 275.4; IR (neat) ν_{max}/cm^{-1} 3369, 3265, 1689, 1586, 1480, 1447, 1350, 1304, 1250, 1177, 1116; ^{1}H NMR (500 MHz, DMSO-d_6) δ 6.97 (1H, dd, *J* = 2.3, 2.3 Hz, H-2'), 7.01 (1H, ddd, *J* = 8.3, 2.4 and 0.9 Hz, H-4' or H-6'), 7.10 (1H, ddd, *J* = 8.2, 2.3 and 0.9 Hz, H-4' or H-6'), 7.35 (1H, ddd, *J* = 8.2, 2.7 and 1.1 Hz, H-4), 7.49 (1H, dd, *J* = 8.2, 8.2 Hz, H-5 or H-5'), 7.52 (1H, dd, *J* = 2.6, 1.5 Hz, H-2), 7.55 (1H, dd, *J* = 7.9, 7.9 Hz, H-5 or H-5'), 7.76 (1H, ddd, *J* = 7.7, 1.3 and 1.3 Hz, H-6), 8.06 (2H, s, ArOSO$_2$N*H*$_2$), 13.09 (1H, s, ArCO$_2$*H*); ^{13}C NMR (126 MHz, DMSO-d_6) δ 112.8 (C-2'), 116.8 (C-4' or C-6'), 117.6 (C-4' or C-6'), 119.0 (C-2), 123.4 (C-4), 124.8 (C-6), 130.6 (C-5 or C-5'), 131.0 (C-5 or C-5'), 132.9 (C-1), 151.2 (C-3'), 156.2 (C-3 or C-1'), 157.1 (C-3 or C-1'), 166.6 (Ar*C*O$_2$H); LRMS (ES$^-$) *m/z* 308.2 [M-H]$^-$; HRMS (ESI) calcd for $C_{13}H_{10}NO_6S$ [M-H]$^-$ 308.0234, found 308.0225.

4-(3-(Sulfamoyloxy)phenoxy)benzoic acid, (75)

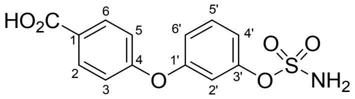

Compound **75** was synthesised according to general procedure B, using the following reagents: 4-(3-((*N*,*N*-bis(2,4-dimethoxybenzyl)sulfamoyl)oxy)phenoxy)benzoic acid (**72**) (200 mg, 0.33 mmol), DCM (3.0 mL) and TFA (0.3 mL). The crude product was purified by column chromatography (silica gel, petrol:EtOAc:AcOH, 1:0:0 → 50:49.7:0.3) to yield the *title compound* as an off-white solid (93 mg, 92%); R_f = 0.32 (petrol:EtOAc:AcOH, 50:49.7:0.3); m.p. 173.5-175.5 °C; λ_{max} (EtOH)/nm 252.2; IR (neat) ν_{max}/cm^{-1} 3368, 3286, 1669, 1593, 1485, 1429, 1366, 1248, 1181, 1161, 1111; ^{1}H NMR (500 MHz, DMSO-d_6) δ 7.02 (1H, dd, *J* = 2.3, 2.3 Hz, H-2'), 7.09 (1H, ddd, *J* = 8.2, 2.4 and 0.9 Hz, H-4' or H-6'), 7.16 – 7.10 (3H, m, H-3, 5 and H-4' or H-6'),

7.52 (1H, dd, J = 8.2, 8.2 Hz, H-5’), 7.97 (2H, d, J = 8.8 Hz, H-2, 6), 8.06 (2H, s, $ArOSO_2NH_2$), 12.79 (1H, s, $ArCO_2H$); ^{13}C NMR (126 MHz, DMSO-d_6) δ 113.7 (C-2’), 117.7 (C-4’ or C-6’), 117.8 (C-3, 5), 118.3 (C-4’ or C-6’), 125.9 (C-1), 131.1 (C-5’), 131.7 (C-2, 6), 151.2 (C-3’), 156.1 (C-1’), 160.2 (C-4), 166.7 ($ArCO_2H$); LRMS (ES$^-$) *m/z* 308.2 [M-H]$^-$; HRMS (ESI) calcd for $C_{13}H_{10}NO_6S$ [M-H]$^-$ 308.0234, found 308.0226.

3-Phenylphenyl sulfamate (76)

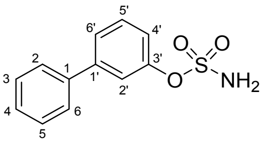

To 3-phenylphenol (204 mg, 1.20 mmol) in DMA (2 mL), cooled at 0 °C, was added a solution of sulfamoyl chloride in acetonitrile (≈ 1.8 M, 1.33 mL, 2.4 mmol). The resulting mixture was stirred overnight at RT, quenched with water (10 mL), and then extracted with EtOAc (2 × 20 mL). The pooled organic extracts were washed with water and brine (20 mL, respectively), dried over $MgSO_4$ and concentrated *in vacuo*. The resulting crude solid was purified by column chromatography (silica gel, petrol:EtOAc, 9:1 → 6:4) to yield the *title compound* as a white solid (128 mg, 43%); R_f = 0.2 (petrol:EtOAc, 8:2); m.p. 91-93 °C; λ_{max} (EtOH)/nm 248.9; IR (neat) ν_{max}/cm^{-1} 3359, 3263, 1353, 1147; ^{1}H NMR (500 MHz; $CDCl_3$) δ 4.95 (2H, s, $ArOSO_2NH_2$), 7.30-7.70 (9H, m, 9 × ArH); ^{13}C NMR (125 MHz; DMSO-d^6) δ 120.4 (C-Ar), 121.2 (C-Ar), 124.9 (C-Ar), 126.8 (C-Ar), 128.0 (C-Ar), 129.0 (C-Ar), 130.3 (C-Ar), 139.0 (C-Ar), 142.0 (C-Ar), 150.7 (C-O); HRMS calcd for $C_{12}H_{10}NO_3S$ [M-H]$^-$ 248.0387, found 248.0391.

[1,1’-Biphenyl]-3-yl dimethylsulfamate (77)

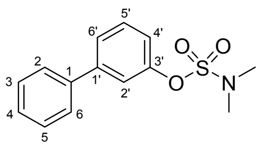

To 3-phenylphenol (274 mg, 1.61 mmol) in acetonitrile (5 mL) was added caesium carbonate (579 mg, 1.77 mmol) and the resulting solution was stirred at RT for 1 h before dropwise addition of *N,N*-dimethylsulfamoyl chloride (154 mg, 0.12 mL, 1.07 mmol) in acetonitrile (1.2 mL). After 20 h, the reaction mixture was filtered through Celite and the solvent removed *in vacuo*. The resulting residue was dissolved in Et_2O

(10 mL), washed with water (10 mL), and the aqueous layer was extracted twice with Et_2O (15 mL). The combined organic layers were dried over $MgSO_4$, filtered and evaporated. The crude product was purified by column chromatography (C18 silica gel, 20-80% acetonitrile/water, formic acid 0.1%) to yield the *title compound* as a white solid (193 mg, 65%); R_f = 0.14 (50% MeCN (0.1% formic acid)/H_2O); m.p. 51.1-54.6 °C; λ_{max} (EtOH)/nm 249; IR (neat) ν_{max}/cm^{-1} 1369, 1178, 1140; ^{1}H NMR (500 MHz; CDCl3) δ 3.01 (6H, s, $ArOSO_2N(CH_3)_2$), 7.26-7.29 (1H, m, Ar*H*), 7.34-7.63 (8H, m, 8 × Ar*H*); ^{13}C NMR (125 MHz; CDCl3) δ 38.8 (2 × *C*H3), 120.4 (C-Ar), 120.5 (C-Ar), 125.5 (C-Ar), 127.2 (2 × C-Ar), 127.6 (C-Ar), 128.9 (2 × C-Ar), 130.0 (C-Ar), 139.8 (C-Ar), 143.3 (C-Ar), 150.7 (C-Ar); LRMS (ES$^+$) *m/z* 278.2 $[M+H]^+$; HRMS calcd for $C_{14}H_{16}NO_3S$ $[M+H]^+$ 278.0845, found 278.0846.

3-Phenoxyphenyl sulfamate, (78)

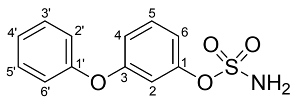

To 3-phenoxyphenol (200 mg, 1.07 mmol) in DMA (2.2 mL), cooled at 0 °C, was added sulfamoyl chloride (1.19 mL, 2.15 mmol). The resulting mixture was stirred overnight at RT, quenched with water (10 mL), and then extracted with EtOAc (2 × 20 mL). The pooled organic extracts were washed with water and brine (20 mL, respectively), dried over $MgSO_4$ and concentrated *in vacuo*. The crude product was purified by column chromatography (silica gel, petrol:EtOAc, 1:0 → 85:15) to yield the *title compound* as a white solid (265 mg, 93%); R_f = 0.31 (petrol:EtOAc, 85:15); m.p. 48.5-50.5 °C; λ_{max} (EtOH)/nm 270.8, 277.6; IR (neat) ν_{max}/cm^{-1} 3367, 3263, 3102, 3070, 3039, 1585, 1490, 1473, 1457, 1362, 1157; ^{1}H NMR (500 MHz, DMSO-d_6) δ 6.91 (1H, dd, *J* = 2.3, 2.3 Hz, H-2), 6.96 (1H, dd, *J* = 8.2, 1.8 Hz, H-4 or H-6), 7.05 (1H, dd, *J* = 8.1, 1.6 Hz, H-4 or H-6), 7.08 (2H, d, *J* = 7.7 Hz, 2 × Ar*H*), 7.20 (1H, dd, *J* = 7.4, 7.4 Hz, H-4'), 7.40 – 7.48 (3H, m, 3 × Ar*H*), 8.04 (2H, s, $ArOSO_2NH_2$); ^{13}C NMR (126 MHz, DMSO-d_6) δ 112.2 (C-2), 116.2 (C-4 or C-6), 116.9 (C-4 or C-6), 119.2 (C-2', 6'), 124.1 (C-4'), 130.2 (C-3', 5'), 130.8 (C-5), 151.1 (C-1), 155.8 (C-3 or C-1'), 157.7 (C-3 or C-1'); LRMS (ES$^-$) *m/z* 264.1 $[M-H]^-$; HRMS (NSI) calcd for $C_{12}H_{11}NO_4SNa$ $[M+Na]^+$ 288.0301, found 288.0299.

2,2,2-Trichloroethyl [1,1'-biphenyl]-3-ylsulfamate, (79)

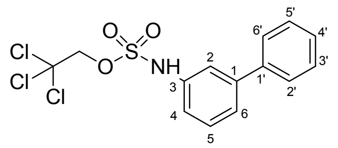

Compound **79** was synthesised according to general procedure F, using the following reagents: 3-aminobiphenyl (200 mg, 1.18 mmol), 2,3-dimethyl-1-((2,2,2-trichloroethoxy)sulfonyl)-1*H*-imidazol-3-ium tetrafluoroborate (**44**) (1.40 g, 3.55 mmol) and THF (11.8 mL). The crude product was purified by column chromatography (silica gel, petrol:EtOAc, 1:0 → 85:15) to yield the *title compound* as a yellow solid (383 mg, 85%); R_f = 0.29 (petrol:EtOAc, 85:15; $KMnO_4$); m.p. 103.5-105.5 °C; λ_{max} (EtOH)/nm 245.5; IR (neat) ν_{max}/cm^{-1} 3273, 1610, 1592, 1572, 1485, 1411, 1366, 1181, 1090, 1013, 946; ^{1}H NMR (500 MHz, $CDCl_3$) δ 4.68 (2H, s, C$H_2$$CCl_3$), 6.75 (1H, s, ArN*H*$SO_3$), 7.26 – 7.23 (1H, m, Ar*H*), 7.41 – 7.36 (1H, m, Ar*H*), 7.49 – 7.42 (5H, m, 5 × Ar*H*), 7.58 – 7.54 (2H, m, 2 × Ar*H*); ^{13}C NMR (126 MHz, $CDCl_3$) δ 79.2 (*C*H_2CCl_3), 93.1 (CH_2*C*Cl3), 120.2 (CH Ar), 120.3 (CH Ar), 125.1 (CH Ar), 127.3 (CH Ar), 128.1 (CH Ar), 129.1 (CH Ar), 130.1 (CH Ar), 135.7 (C_q Ar), 140.0 (C_q Ar), 143.1 (C_q Ar); LRMS (ES$^-$) *m/z* 378.1 [M(^{35}Cl^{35}Cl^{35}Cl)-H]$^-$, 380.1 [M(^{37}Cl^{35}Cl^{35}Cl)-H]$^-$, 382.1 [M(^{37}Cl^{37}Cl^{35}Cl)-H]$^-$; HRMS (ESI) calcd for $C_{14}H_{13}Cl_3NO_3S$ [M(^{35}Cl^{35}Cl^{35}Cl)+H]$^+$ 377.9531, found 377.9520.

2,2,2-Trichloroethyl (3-phenoxyphenyl)sulfamate (80)

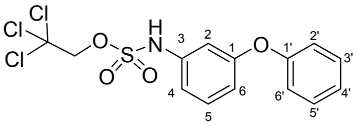

Compound **80** was synthesised according to general procedure F, using the following reagents: 3-phenoxyaniline (100 mg, 0.54 mmol, 1 equiv.), 2,3-dimethyl-1-((2,2,2-trichloroethoxy)sulfonyl)-1*H*-imidazol-3-ium tetrafluoroborate (**44**) (640 mg, 1.61 mmol, 3 equiv.) and THF (10 mL). The crude product was purified by column chromatography (silica gel, petrol:EtOAc, 99:1 → 80:20) to give a brown oil which was re-purified by column chromatography (silica gel, petrol:DCM, 60:40 → 0:1) to give the title compound as a white solid; R_f 0.25 (50% DCM/petrol); ^{1}H NMR (500 MHz; $CDCl_3$) δ_H 4.66 (OCH_2), 6.59 (NH), 6.84-6.89 (2H, m, H-Ar), 6.96 (1H, ddd, *J* = 0.8, 2.2 and 8.0 Hz), 7.00-7.05 (2H, m, H-Ar), 7.12-7.18 (1H, m, H-Ar), 7.31 (1H, td,

J = 0.5 and 8.0 Hz, H-Ar), 7.33-7.39 (2H, m, H-Ar); ^{13}C NMR (125 MHz; $CDCl_3$) δ_C 79.0 (CCl_3), 92.9 (OCH_2), 111.1, 115.3, 115.9, 119.5, 124.1, 130.0, 130.7, 136.5, 156.2, 158.7; MS ES+ 396.2, 398.1, 400.2.

[1,1'-biphenyl]-3-yl (2,2,2-trichloroethyl) sulfate, (81)

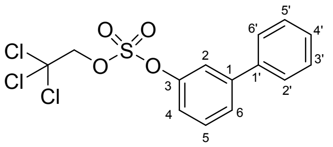

To 3-phenylphenol (300 mg, 1.76 mmol) in THF (17 mL), cooled in an ice bath, were added 4-(dimethylamino)pyridine (323 mg, 2.64 mmol) and triethylamine (491 µL, 356 mg, 3.52 mmol). The resulting solution was stirred at 0 °C for 10 min, and then a solution 2,2,2-trichloroethyl chlorosulfate (42) (469 µL, 852 mg, 3.52 mmol) in THF (8.5 mL) was added dropwise over 15 min. The resulting solution was stirred in an ice bath for 1 h and allowed to warm to RT. The reaction mixture was left stirring for an additional 24 h. Upon completion, the mixture was diluted with EtOAc (20 mL), quenched by the cautious addition of saturated aq. $NaHCO_3$ (20 mL) and extracted with EtOAc (3 × 20 mL). The pooled organic extracts were washed with water and brine (40 mL, respectively), dried over $MgSO_4$ and concentrated *in vacuo*. The crude product was purified by column chromatography (silica gel, petrol:EtOAc, 1:0 → 95:5) to yield the *title compound* as a white solid (732 mg, 96%); R_f = 0.42 (petrol:EtOAc, 95:5; $KMnO_4$); m.p. 45.5-47.5 °C; λ_{max} (EtOH)/nm 248.5; IR (neat) ν_{max}/cm^{-1} 1607, 1570, 1477, 1441, 1406, 1200, 1137; ^{1}H NMR (500 MHz, CDCl3) δ 4.86 (s, 2H, OC$H_2$$CCl_3$), 7.67 – 7.31 (m, 9H, 9 × Ar*H*); ^{13}C NMR (126 MHz, CDCl3) δ 80.6, 92.5, 119.7, 119.9, 121.5, 126.7, 127.3, 129.2, 130.5, 139.4, 143.9, 150.7; HRMS (NSI) calcd for $C_{14}H_{11}Cl_3O_4SNa$ $[M+Na]^+$ 402.9336, found 402.9339.

2,2,2-Trifluoroethyl [1,1'-biphenyl]-3-ylsulfamate (82)

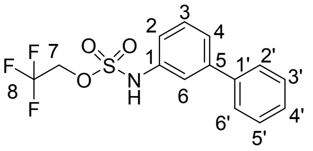

To a microwave vial was added 3-aminobiphenyl (98 mg, 0.58 mmol) and **S8** (600 mg, 1.73 mmol) in ACN (10 mL). The vial was sealed and heated conventionally to 50°C for 48 h. TLC monitoring indicated the reaction had gone to completion. The reaction

mixture was dried *in vacuo*. This was purified by column chromatography (0 – 40% EtOAc in PE) to afford **82** as a brown solid (12 mg, 4%): R_f= 0.35 (1:2 EtOAc in PE); mp: 75-80°C; λ_{max} 205 nm, 223 nm, 248 nm; IR (cm^{-1}) 3300, 2323, 1607, 1472; ^1H NMR (500 MHz, $CDCl_3$) δ 4.47 (2H, q, J = 7.9 Hz, CH_2), 7.20 (1H, ddd, J = 7.4, 2.4, 1.5 Hz, H-2), 7.38 (1H, tt, J = 7.3, 1.3 Hz, H-4') 7.42 (1H, dd, J = 2.4, 1.5 Hz, H-6) 7.46 (4H, m, Ar-H), 7.55 (1H, m, Ar-H), 7.57 (1H, dd, J = 1.5, 1.4 Hz, H-3); ^{19}F NMR (500 MHz, $CDCl_3$) δ -73.65 (s); ^{13}C NMR (500 MHz, $CDCl_3$) δ 66.1 (q, J = 37.5 Hz, C-7), 112.2 (s, C-4), 120.3 (s, C-2 or 6), 120.3 (s, Ar-C), 120.9 (s, C-2 or 6), 123.1 (s, C-3'), 125.4 (s, Ar-C), 127.3 (s, Ar-C), 128.11 (s, Ar-C), 129.1 (s, Ar-C), 130.2 (s, Ar-C), 135.5 (s, C-1, 5 or 1'), 140.0 (s, C-1', 1 or 5), 143.2 (s, C-5, 1 or 1').

2,2,2-Trichloroethyl phenylsulfamate (83)

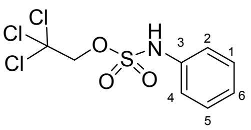

Compound **83** was synthesised according to general procedure F, using the following reagents: aniline (100 mg, 1.08 mmol, 1 equiv.), 2,3-dimethyl-1-((2,2,2-trichloroethoxy)sulfonyl)-1*H*-imidazol-3-ium tetrafluoroborate (**44**) (1.28 g, 3.22 mmol, 3 equiv.) and THF (10 mL). The crude product was purified by column chromatography (silica gel, petrol:EtOAc, 98:2 → 80:20) to give a the title compound as a white solid (228 mg, 70%); R_f 0.3 (10% EtOAc/petrol); ^1H NMR (500 MHz; $CDCl_3$) δ_H 4.64 (2H, s, OCH_2), 6.63 (1H, s, NH), 7.21-7.28 (3H, m, H-Ar), 7.35-7.41 (2H, m, H-Ar); ^{13}C NMR (125 MHz; $CDCl_3$) δ_C 79.0 (CCl_3), 93.0 (OCH_2), 121.5, 126.3, 129.7, 135.2; MS ES- 302.0, 304.0, 306.0.

1,1'-sulfonylbis(2-methyl-1*H*-imidazole), (S2)

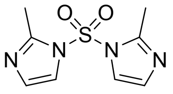

To 2-methylimidazole (20.3 g, 247 mmol) in DCM (100 mL), cooled at 0 °C, was added dropwise over 30 min sulfuryl chloride (**S1**) (5 mL, 8.32 g, 61.7 mmol) in DCM (40 mL). The resulting solution was stirred at 0 °C for 1 h and allowed to warm to RT. After 24 h, the reaction was quenched by the cautious addition of water (100 mL) and extracted with DCM (2 × 50 mL). The pooled organic extracts were washed with brine

(100 mL), dried over $MgSO_4$ and concentrated *in vacuo*. The crude yellow solid was purified by column chromatography (silica gel, DCM:MeOH, 1:0 → 93:7) to yield the *title compound* as an off-white solid (10.2 g, 73%); R_f = 0.32 (DCM:MeOH, 93:7; $KMnO_4$); m.p. 86.0-88.0 °C (lit. 90.0-91.0 °C)[4]; No λ_{max} (EtOH)/nm; IR (neat) ν_{max}/cm^{-1} 3116, 1553, 1419, 1147, 1052; ^{1}H NMR (500 MHz, $CDCl_3$) δ 2.52 (6H, s, 2 × CH_3), 6.95 (2H, d, *J* = 1.7 Hz, H-4 or H-5), 7.37 (2H, d, *J* = 1.8 Hz, H-4 or H-5); ^{13}C NMR (126 MHz, $CDCl_3$) δ 15.1 (CH_3), 120.2 (C-4 or C-5), 128.6 (C-4 or C-5), 146.1 (C-2); LRMS (ES$^+$) *m/z* 227.2 [M+H]$^+$; HRMS (ESI) calcd for $C_8H_{11}N_4O_2S$ [M+H]$^+$ 227.0597, found 227.0597; ^{1}H NMR and IR data were identical to literature data.[4]

3-Bromophenyl 2-methyl-1*H*-imidazole-1-sulfonate, (S4)

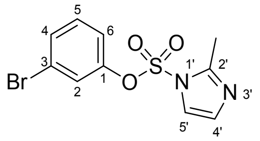

To 1,1'-sulfonylbis(2-methyl-1*H*-imidazole) (**S2**) (2.61 g, 11.6 mmol) and caesium carbonate (2.07 g, 6.36 mmol) in acetonitrile (20 mL) was added 3-bromophenol (**S3**) (1.0 g, 5.78 mmol). The resulting mixture was heated at 120 °C for 15 min under microwave irradiation. After cooling, the mixture was concentrated *in vacuo*. The resulting residue was dissolved in a saturated aq. NH_4Cl (15 mL) and the mixture extracted with EtOAc (3 × 15 mL). The combined organic extracts were washed with water (20 mL) and brine (20 mL), dried over $MgSO_4$ and concentrated *in vacuo*. The crude yellow oil was purified by column chromatography (silica gel, petrol:EtOAc, 1:0 → 8:2) to yield the *title compound* as a pale yellow oil (1.39 g, 76%); R_f = 0.32 (petrol:EtOAc, 8:2; $KMnO_4$); λ_{max} (EtOH)/nm 270.5; IR (neat) ν_{max}/cm^{-1} 1581, 1554, 1467, 1424, 1208, 1152, 890; ^{1}H NMR (500 MHz, $CDCl_3$) δ 2.51 (3H, s, CH_3), 6.83 (1H, ddd, *J* = 8.3, 2.4 and 0.9 Hz, H-6), 6.91 (1H, d, *J* = 1.8 Hz, H-4' or H-5'), 7.13 (1H, d, *J* = 1.8 Hz, H-4' or H-5'), 7.18 (1H, dd, *J* = 2.1, 2.1 Hz, H-2), 7.23 (1H, dd, *J* = 8.2, 8.2 Hz, H-5), 7.50 (1H, ddd, *J* = 8.0, 1.7 and 0.9 Hz, H-4); ^{13}C NMR (126 MHz, $CDCl_3$) δ 15.1 (CH_3), 120.3 (C-4' or C-5' or C-6), 120.5 (C-4' or C-5' or C-6), 123.2 (C-3), 125.4 (C-2), 128.2 (C-4' or C-5'), 131.4 (C-5), 132.0 (C-4), 146.8 (C-1 or C-2'), 149.2 (C-1 or C-2'); LRMS (ES$^+$) *m/z* 316.9 [M(^{79}Br)+H]$^+$, 319.1 [M(^{81}Br)+H]$^+$; HRMS (ESI) calcd for $C_{10}H_{10}BrN_2O_3S$ [M(^{79}Br)+H]$^+$ 316.9590, found 316.9597.

1-((3-Bromophenoxy)sulfonyl)-2,3-dimethyl-1*H*-imidazol-3-ium tetrafluoroborate, (S5)

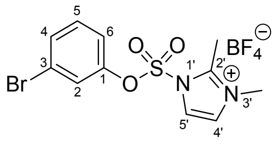

To 3-bromophenyl 2-methyl-1*H*-imidazole-1-sulfonate (**S4**) (5.5 g, 17.4 mmol) in DCM (70 mL), cooled at 0 °C, was added trimethyloxonium tetrafluoroborate (2.57 g, 17.4 mmol). The resulting solution was stirred at 0 °C for 1 h and allowed to warm to RT. Upon completion, the reaction was cooled in an ice bath. Cold petrol (120 mL) was added to the mixture, leading to the precipitation of **S5**. The white precipitate was filtered off, washed with cold petrol (60 mL) and dry under high vacuum. The white fluffy solid (6.20 g, 85%) was used in the next step without further purification; m.p. 149.5-151.5 °C; λ_{max} (EtOH)/nm 265.5; IR (neat) ν_{max}/cm^{-1} 3159, 3103, 1594, 1580, 1447, 1229, 1214, 1149, 1031, 906; ^{1}H NMR (500 MHz, MeOD) δ 2.91 (3H, s, CH_3), 4.86 (3H, s, NCH_3), 7.29 (1H, ddd, *J* = 8.5, 2.5 and 0.9 Hz, H-6), 7.45 (1H, dd, *J* = 8.2, 8.2 Hz, H-5), 7.66 (1H, dd, *J* = 2.1, 2.1 Hz, H-2), 7.72 – 7.68 (1H, m, H-4), 7.73 (1H, d, *J* = 2.4 Hz, H-4’ or H-5’), 7.87 (1H, d, *J* = 2.5 Hz, H-4’ or H-5’); ^{13}C NMR (126 MHz, MeOD) δ 11.9 (CH_3), 36.8 (NCH_3), 121.5 (C-6), 122.4 (C-4’ or C-5’), 124.3 (C-3), 125.0 (C-4’ or C-5’), 126.2 (C-2), 133.4 (C-5), 134.0 (C-4), 150.7 (C-1); ^{19}F NMR (471 MHz, MeOD) δ -154.4 (BF_4^-); LRMS (ES$^+$) *m/z* 331.1 [M(^{79}Br)-BF_4]$^+$, 333.2 [M(^{81}Br)-BF_4]$^+$; HRMS (ESI) calcd for $C_{11}H_{12}BrN_2O_3S$ [M(^{79}Br)-BF_4]$^+$ 330.9747, found 330.9751.

2,2,2-Trifluoroethyl chlorosulfate (S6)

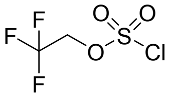

To sulfuryl chloride (0.54 mL, 6.68 mmol) in Et_2O (5 mL), at -78°C, was added dropwise over 35 mins a solution of 2,2,2-trifluoroethanol (0.49 mL, 6.68 mmol) and pyridine (0.54 mL, 6.68 mmol) in Et_2O (5 mL). The resulting mixture was stirred at -78°C for 1 h. The reaction mixture was the allowed to warm to room temperature and stirred overnight. The salt by-product was filtered off and washed with Et_2O. The filtrate was dried *in vacuo* to afford **S6** as a clear oil (618 mg, 47%): ^{1}H NMR (500 MHz, $CDCl_3$) δ 4.72 (2H, q, *J* = 7.5 Hz, CH_2).

2,2,2-Trifluoroethyl 2-methyl-1*H*-imidazole-1-sulfonate (S7)

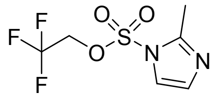

To a suspension of 2-methylimidazole (372 mg, 4.53 mmol) in THF (3 mL), at 0°C, was added dropwise a solution of 2,2,2-trifluoroethyl chlorosulfate (**S6**) (300 mg, 1.51 mmol) in THF (3 mL). The resulting mixture was stirred for 1 hr at 0°C then allowed to warm to room temperature and stirred overnight. The remaining 2-methylimidazole was then filtered off, washed with THF and the filtrate was concentrated *in vacuo*. The crude residue was partitioned between EtOAc (50 mL) and water (50 mL). The aqueous layer was washed with further EtOAc (50 mL) and the organics were combined, washed with brine (50 mL), dried over $MgSO_4$ and concentrated *in vacuo* to afford a clear oil (300 mg). This procedure was repeated and the two batches combined to afford crude product (706 mg). This was purified by column chromatography (silica gel, PE:EtOAc 3:2) to afford **S7** as an off-white solid (587 mg, 77%): *Rf* = 0.36 (PE:EtOAc 3:2); mp: 52-56°C; λ_{max} = 218 nm; ^{1}H NMR (500 MHz, $CDCl_3$) δ 2.63 (3H, s, CH_3), 4.46 (2H, q, J = 7.7 Hz, CH_2), 6.96 (1H, d, J = 1.9 Hz, H-4), 7.29 (1H, d, J = 1.9 Hz, H-5).

2,3-Dimethyl-1-((2,2,2-trifluoroethoxy)sulfonyl)-1*H*-imidazol-3-ium tetrafluoroborate (S8)

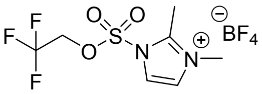

To a solution of 2,2,2-trifluoroethyl 2-methyl-1*H*-imidazole-1-sulfonate (**S7**) (558 mg, 2.28 mmol) in DCM (8 mL), at 0°C, was added trimethyloxonium tetrafluoroborate (337 mg, 2.28 mmol) portionwise over 15 min. The reaction mixture was stirred at 0°C for 1 h then allowed to warm to room temperature and stirred overnight. After approx. 20 h, the reaction mixture was dried *in vacuo* to afford **S8** as a pale gum (600 mg, 76%); mp: 95-102°C; λ_{max} 220 nm; IR (cm^{-1}) 3160, 2123, 1611, 1430; ^{1}H NMR (500 MHz, DMSO) δ 2.82 (3H, s, C-CH_3), 3.87 (3H, s, N-CH_3), 5.46 (2H, q, CH_2), 7.97 (1H, d, J = 2.5 Hz, H-5), 8.21 (1H, d, J = 2.5 Hz, H-4).

Sulfamoyl chloride, (S9)

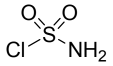

Formic acid (1.07 mL, 1.3 g, 28.2 mmol) was added dropwise to chlorosulfonyl isocyanate (2.46 mL, 4.0 g, 28.2 mmol) in anhydrous acetonitrile (12 mL) at 0 °C. Gentle gas evolution was observed. The mixture was allowed to warm to RT and stirred at RT for 3 h to give a solution of the *title compound* in acetonitrile (≈ 1.8 M).

References

(1) Morimoto-Tomita, M.; Uchimura, K.; Werb, Z.; Hemmerich, S.; Rosen, S. D. *J. Biol. Chem.* **2002**, *277*, 49175.

(2) Furstner, A.; Domostoj, M. M.; Scheiper, B. *J. Am. Chem. Soc.* **2006**, *128*, 8087.

(3) Ingram, L. J.; Desoky, A.; Ali, A. M.; Taylor, S. D. *J. Org. Chem.* **2009**, *74*, 6479.

(4) Beaudoin, S.; Kinsey, K. E.; Burns, J. F. *J. Org. Chem.* **2003**, *68*, 115.
